# *N*^1^-methyladenosine methylation in tRNA drives liver tumourigenesis by regulating cholesterol metabolism

**DOI:** 10.1038/s41467-021-26718-6

**Published:** 2021-11-02

**Authors:** Yanying Wang, Jing Wang, Xiaoyu Li, Xushen Xiong, Jianyi Wang, Ziheng Zhou, Xiaoxiao Zhu, Yang Gu, Dan Dominissini, Lei He, Yong Tian, Chengqi Yi, Zusen Fan

**Affiliations:** 1 CAS Key Laboratory of Infection and Immunity, CAS Center for Excellence in Biomacromolecules, Institute of Biophysics, Chinese Academy of Sciences, 100101 Beijing, China; 2 State Key Laboratory of Protein and Plant Gene Research, School of Life Sciences, Peking-Tsinghua Center for Life Sciences, Peking University, Beijing, China; 3 CAS Key Laboratory of RNA Biology; Institute of Biophysics, Chinese Academy of Sciences, 100101 Beijing, China; 4 University of Chinese Academy of Sciences, 100049 Beijing, China; 5 Cancer Research Center and Wohl Institute for Translational Medicine, Chaim Sheba Medical Center, Sackler Faculty of Medicine, Tel Aviv University, 6997801 Tel Aviv, Israel; 6 Department of Hepatobiliary Surgery, PLA General Hospital, 100853 Beijing, China

**Keywords:** Hepatocellular carcinoma, Cancer metabolism, Cancer stem cells

## Abstract

Hepatocellular carcinoma (HCC) accounts for the majority of primary liver cancers and is characterized by high recurrence and heterogeneity, yet its mechanism is not well understood. Here we show that *N*^1^-methyladenosine methylation (m^1^A) in tRNA is remarkably elevated in hepatocellular carcinoma (HCC) patient tumour tissues. Moreover, m^1^A methylation signals are increased in liver cancer stem cells (CSCs) and are negatively correlated with HCC patient survival. TRMT6 and TRMT61A, forming m^1^A methyltransferase complex, are highly expressed in advanced HCC tumours and are negatively correlated with HCC survival. TRMT6/TRMT61A-mediated m^1^A methylation is required for liver tumourigenesis. Mechanistically, TRMT6/TRMT61A elevates the m^1^A methylation in a subset of tRNA to increase PPARδ translation, which in turn triggers cholesterol synthesis to activate Hedgehog signaling, eventually driving self-renewal of liver CSCs and tumourigenesis. Finally, we identify a potent inhibitor against TRMT6/TRMT61A complex that exerts effective therapeutic effect on liver cancer.

## Abstract (web summary)

Metabolic adaptation has been reported to promote cancer, yet the underlying mechanisms are not clear. Here, the authors show that m^1^A methylation in tRNA regulates cholesterol metabolism in liver cancer stem cells and m^1^A inhibition decreases tumourigenesis in preclinical models of hepatocellular carcinoma.

## Introduction

Liver cancer is the fourth most common cancer-related death and is the sixth in terms of cancer morbidity worldwide^1,2^. Hepatocellular carcinoma (HCC) accounts for the majority of primary liver cancers, with a 5-year survival less than 20%. HCC occurs mostly as a cause of chronic infection of hepatitis B or C virus (HBV or HCV) along with alcohol abuse. Nonalcoholic fatty liver disease (NAFLD) with metabolic syndrome also increases the risk of liver cancer. Chronic liver disease can cause cirrhosis that accelerates a series of genetic and epigenetic changes in the formation of HCC^3^. Liver cancer remains difficult to treat because of lacking drugs for critical targets^4^. Sorafenib and lenvatinib, as inhibitors of multiple kinases, remain the effective option for frontline therapy. However, they provide only a modest benefit to patients with HCC^5^ and the median survival was 13.6 months for lenvatinib and 12.3 months for sorafenib^3^, which highlight the urgent need to develop new treatment measures. New therapies are likely from deep understanding of the mechanisms of HCC tumourigenesis. HCC is characterized by high recurrence and heterogeneity^6^. Heterogeneity is mainly caused by the hierarchical organization of tumor cells with a subset of cells known as cancer stem cells (CSCs)^2^. These CSCs within tumor bulk display capacity to self-renew, differentiate, and give rise to a new tumor^7^, accounting for a hierarchical organization of heterogeneous cancer cells and a high rate of recurrence. However, how liver CSCs maintain their self-renewal remains largely unknown.

RNA modifications have recently become critical posttranscriptional regulators of gene expression. *N*^1^-methyladenosine (m^1^A) methylation at position 58 (m^1^A58) in tRNA is an essential and highly conserved modification^8,9^. The deposition of m^1^A in tRNA is largely dependent on secondary structure^10^, occurring in all processes of life^8^. m^1^A58 in tRNA can be catalyzed by a methyltransferase complex, composed of an RNA binding component TRMT6 and a catalytic component TRMT61A^11^. Recently, m^1^A58 in tRNA is shown to be reversible and can be demethylated by ALKBH1^12^. In initiator tRNA^Met^, m^1^A58 is important for maintaining its stability and influencing translation initiation^13^. And in other tRNA species, m^1^A methylation promotes translation through association with translation-active polysomes, as it is preferentially bound to eEF1α, a known elongation factor protein that delivers aminoacyl tRNA to polysomes^12^. m^1^A58 methylation in tRNA has profound effects on various biological processes, including HIV replication^14^ and cell viability^13^. However, roles of m^1^Amethylation in cancers remain largely unknown.

Most tumors have an aberrantly activated lipid metabolism^15,16^, which enables them to promote tumor proliferation. However, only particular subsets of cancer cells are sensitive toward targeting lipid metabolism. This suggests that many cancer cells harbor unexplored mechanisms in their lipid metabolism. Cholesterol is an essential lipid for maintaining cellular homeostasis and is primarily synthesized in the liver and transported to cells around the body^17^. Cellular cholesterol homeostasis is regulated by the synthesis, influx, efflux, and metabolism^18^. In cancer, homeostatic processes can be disturbed to help cell survival and aberrant growth. Although cholesterol metabolism is dysregulated in numerous malignancies^19,20^, intestinal stem cells^21^, and hematopoietic stem cells^22^, how it affects liver CSCs and cancer progression remains largely unknown. Cholesterol biosynthesis is mainly regulated by sterol regulatory element- binding protein 2 (SREBP2 or SREBF2), 3-hydroxy-3-methylglutaryl coenzyme A reductase (HMGCR) and squalene monooxygenase (SQLE)^17^. In addition to cardiovascular disease, cholesterol lowering strategy is a promising treatment of cancers^23^. More and more recent studies support that cholesterol or other related sterols is sufficient for Smoothened (SMO) activation both in cells and in vitro with purified component^24,25^, leading to Hedgehog signaling activation. However, the underlying mechanism remains elusive in liver cancer and liver CSCs. Here, we reveal that m^1^A methylation signals are significantly increased in higher grade HCC, especially in patients with microvascular invasion (MVI). m^1^A signals in RNA are also hypermethylated in liver CSCs. TRMT6/TRMT61A mediated m^1^A methylation participates in liver tumourigenesis through promotion of PPARδ protein translation, leading to cholesterol biogenesis for activation of Hh signaling.

## Results

### m^1^A methylation in RNA is aberrantly elevated in HCC patients and liver CSCs

**Fig. 1:**
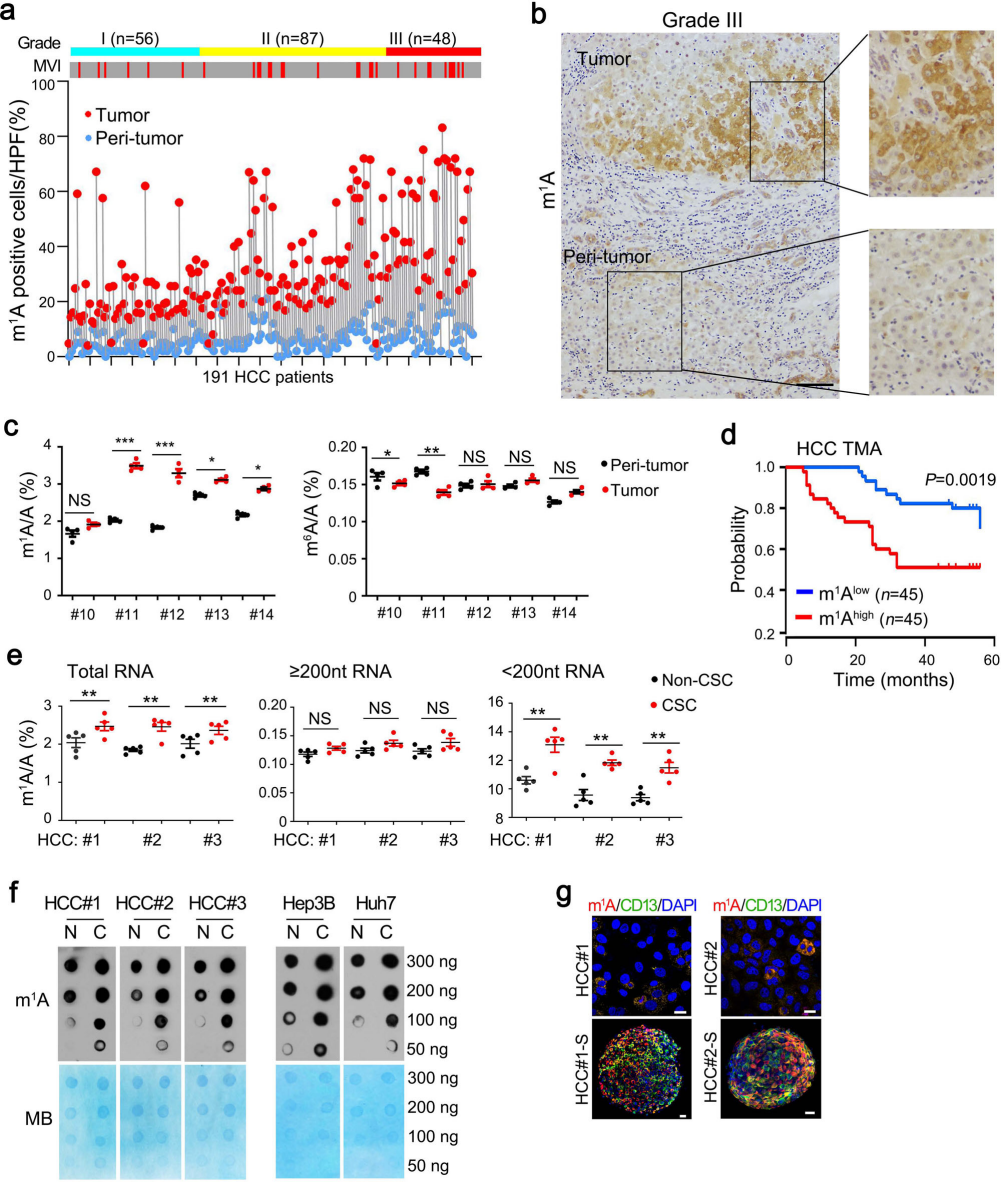
m^1^A methylation in RNA are aberrantly elevated in HCC tumor tissues and liver CSCs. **a** Overview of immunohistochemistry analysis of endogenous m^1^A levels in a panel of 191 clinically defined human HCC samples. Samples are arranged by grades and then sorted by microvascular invasion (MVI) (gray: “no”; red: “yes”). Paired samples are connected with gray lines. **b** Representative immunohistochemistry images of endogenous m^1^A levels (brown) in grade III HCC tumor tissue. Scale bar, 100 μm. *n* = 4 independent experiments. **c** LC-MS/MS quantification of m^1^A/A and m^6^A/A ratios in total RNA purified from HCC samples. Data are means ± SD. *n* = 4 biologically independent experiments. Exact *P* values from left to right: 0.052, 0.00082, 0.00043, 0.026, 0.011, 0.043, 0.0075, 0.13, 0.084, 0.055. **d** Kaplan–Meier plots of overall survival based on m^1^A levels in the tissue microarray assay (TMA) cohort. The median expression value is used as a cut-off. *P* values for Kaplan–Meier curves were calculated using a log-rank test. **e** LC-MS/MS quantification of m^1^A levels in total RNA, large RNA (≥200 nucleotide, nt, mostly composed of rRNA) and small RNA (<200 nt, mostly composed of tRNA) purified from HCC primary CSCs and non-CSCs, presented as percentage of unmodified A. Data are means ± SD. *n* = 5 biologically independent experiments. Exact *P* values from left to right: 0.0052, 0.0063, 0.0065, 0.067, 0.11, 0.075, 0.0017, 0.0089, and 0.0084. **f** Global m^1^A levels were detected in liver CSCs (C) and non-CSCs (N) from HCC primary tumors using dot blot assay. MB (methyl blue) was used as loading controls. *n* = 4 biologically independent samples. **g** Representative immunofluorescence staining of m^1^A and CD13 in HCC primary tumor cells and respective oncosphere cells (S). DAPI, 4,6-diamidino-2-phenylindole. Scale bar, 10 μm. *n* = 4 biologically independent experiments. *P < 0.05; **P < 0.01; ***P < 0.001, and NS, not significant (*P* > 0.05) by two-tailed Student’s *t*-test.

To investigate the role of m^1^A methylation in liver cancer, we selected surgically resected primary HCC tumor tissues and paired peri-tumor tissues from a cohort of 191 HCC patients who had not received previous chemotherapy or radiotherapy (Supplementary Table 1). Of these cases, 187 had an HBV-infection background, and the other four patients had HCV infection. We found that m^1^A levels were aberrantly elevated in 35–65% of HCC tumor tissues using immunohistochemical staining (Fig. 1a and Supplementary Fig. 1a). m^1^A signals were much higher in poorly differentiated (Grade III) than in well differentiated (Grade I) HCC samples (Fig. 1a, b), especially most elevated in samples with microscopic vascular invasion (MVI)(Fig. 1a). However, there was no significant correlation among levels of m^1^A,α-fetoprotein (AFP) (*P* = 0.125), and tumor sizes (*P* = 0.083). Using quantitative mass spectrometry (LC-MS/MS), we further showed that in total RNA and small RNA (<200 nt RNA, mostly tRNA^9^) m^1^A signals were much more elevated in tumor cells than in peri-tumor tissues, whereas *N*^6^-methyladenosine (m^6^A) signals displayed no significant changes (Fig. 1c). In addition, samples with highest levels of m^1^A in tumors exhibited advanced stage (Supplementary Fig. 1b) and worst survival (Fig. 1d) in a tissue microarray (TMA) cohort containing 90 HCC samples. Moreover, m^1^A levels were upregulated in diethylnitrosamine (DEN) induced liver cancer or in *albumin* (*Alb*)-Cre; H11-LSL-*Myc* (specific overexpression of *Myc* in hepatocytes, hereafter called *Myc*^OE^) spontaneous mouse liver cancer tissues (Supplementary Fig. 1c).

Cancer stem cells (CSCs) within tumor bulk harbor a capacity to self-renew and give rise to a new tumor^7^. We then sorted liver CSCs (CD13^+^CD133^+^) and non-CSCs (CD13^−^CD133^−^)^26^ from HCC samples and HCC cell lines. We found that liver CSCs displayed much higher m^1^A levels in total RNA and small RNA fraction by quantitative LC-MS/MS (Fig. 1e). These observations were further verified by dot-blot (Fig. 1f) and immunofluorescence staining assays (Fig. 1g and Supplementary Fig. 1d). As a comparison, levels of m^6^A, *N*^1^-methylguanosine (m^1^G), and pseudouridine (Ψ) showed no significant changes between liver CSCs versus non-CSCs (Supplementary Fig. 1e). Collectively, m^1^A level is aberrantly upregulated in liver CSCs and advanced HCC tumors, especially in samples with MVI.

### TRMT6/TRMT61A complex is required for liver tumourigenesis

**Fig. 2:**
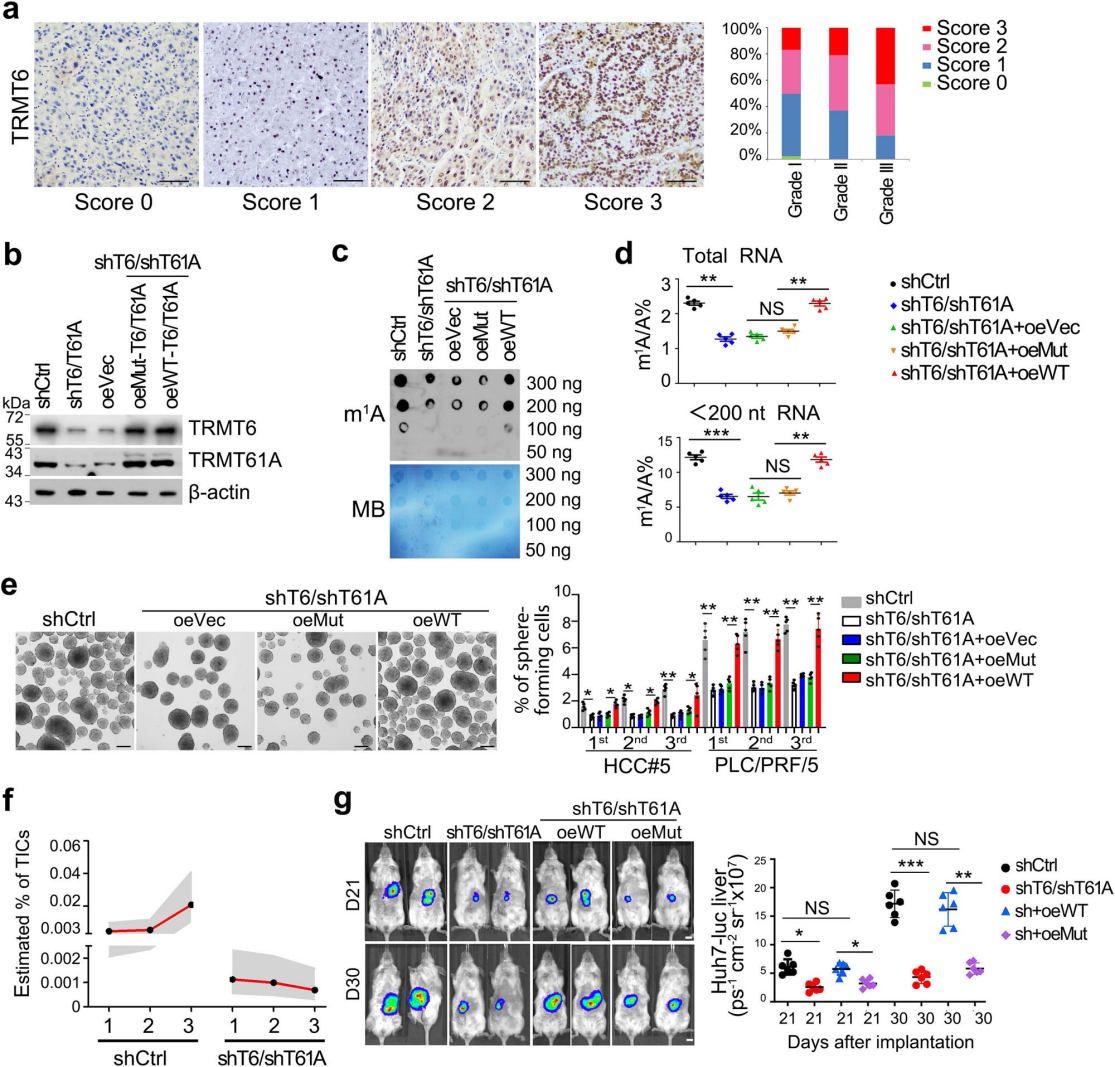
TRMT6/TRMT61A-mediated m^1^A modification drives self-renewal of liver CSCs and tumourigenesis. **a** Representative immunohistochemistry images of TRMT6 protein levels (brown) in the cohort of 191 HCC samples. *n* = 3 independent experiments. Score 0 (0–5% positive cells/tissue section) represents TRMT6 staining that is considered negative; score 1 (5–15% positive cells/section) represents TRMT6 staining that is considered week positive, whereas scores 2 (15–35% positive cells/section) and 3 (>35% positive cells/section) represent TRMT6 staining that is considered positive. Grade I (*n* = 56), Grade II (*n* = 87), Grade III (*n* = 48). Scale bar, 100 μm. **b** Western blotting confirmation of *TRMT6/TRMT61A* (*T6/T61A*) depletion, and co-infection of WT or *TRMT6*^*R377L*^/*TRMT61A*^*D181A*^ (Mut) constructs in TRMT6/TRMT61A depleted cells. These experiments were repeated three times. oe overexpression, Vec vector. *n* = 3 biologically independent samples. **c** Global m^1^A levels were detected in shCtrl, *TRMT6/TRMT61A* depletion, and co-infection of WT or Mut-*TRMT6/TRMT61A* in TRMT6/TRMT61A depleted liver CSCs using dot blot assay. Data were repeated five times. *n* = 5 biologically independent samples. **d** LC-MS/MS quantification of m^1^A levels in total RNA and tRNA (<200 nt) purified from indicated liver CSCs, presented as percentage of unmodified A. Data are means ± SD. *n* = 5 biologically independent samples. Exact *P* values from left to right (upper): 0.0033, 0.052, 0.0087; left to right (lower): 0.00012, 0.22, and 0.0084. **e ***TRMT6/TRMT61A* depletion suppressed serial oncosphere-forming capacity in HCC primary cells and HCC cell lines. Right panel represents statistical results as means ± SD. *n* = 5 biologically independent cells. Scale bar, 100 μm. Exact *P* values from left to right: 0.036, 0.029, 0.031, 0.042, 0.0031, 0.022, 0.0022, 0.0054, 0.0063, 0.0072, 0.0050, 0.0064. **f** Estimated frequency of tumor initiating cells (TICs) in TRMT6/TRMT61A depleted and control liver CSCs during serial transplantations in NSG mice. Data are presented as mean values (black spot) and upper/lower values (gray area). *n* = 10 mice per group. **g** Four kinds of knockdown and rescued Huh7 CSCs with green fluorescent protein (GFP)–luciferase tags (Huh7-Luc) were orthotopically implanted into NSG mice. HCC tumor growth rates of with orthotopic engraftment were monitored in vivo by whole NSG mouse imaging after 21 and 30 days post-transplantation, respectively. Scale bar, 1 cm. Right panel represents statistical results as means ± SD. *n* = 6 mice per group. Exact *P* values from left to right: 0.023, 0.064, 0.040, 0.00071, 0.053, 0.0085. *P < 0.05; **P < 0.01; ***P < 0.001, and NS not significant (*P* > 0.05) by two-tailed Student’s *t*-test.

m^1^A at position 58 (m^1^A58) in tRNA can be catalyzed by a methyltransferase complex, containing an RNA binding component TRMT6 and a catalytic component TRMT61A^27^. Of note, *TRMT6* mRNA levels were dramatically elevated in HCC tumor tissues compared with adjacent peri-tumor tissues (Supplementary Fig. 2a and Supplementary Table 2), which was further confirmed at their protein levels by immunoblotting (Supplementary Fig. 2b). TRMT6 was significantly upregulated in 50–80% of HCC tumor tissues in our cohort of 191 HCC patients (Fig. 2a). Consistent with our cohort, *TRMT6* and *TRMT61A* levels were also remarkably increased in tumor tissues of Wang’s cohort (Supplementary Fig. 2c) and The Cancer Genome Atlas (TCGA) dataset (Supplementary Fig. 2d). In TCGA dataset, samples with highest levels of *TRMT6* and *TRMT61A *in tumors exhibited advanced stages (Supplementary Fig. 2e) and worse survivals (Supplementary Fig. 2f, g). Moreover, TRMT6 was also highly expressed in CSCs of human liver cancer tissues by immunofluorescence staining (Supplementary Fig. 2h) and qRT-PCR analysis (Supplementary Fig. 2i). These results suggest that TRMT6 and TRMT61A are negatively correlated with a poor prognosis of HCC.

To explore whether TRMT6/TRMT61A complex plays a critical role in tumourigenesis, we screened and employed two different lentivirus-mediated short hairpins RNAs (shRNAs) through their depletion efficiencies to silence *TRMT6/TRMT61A *expression in liver CSCs (Supplementary Table 3). TRMT6/TRMT61A depletion markedly reduced their protein levels (Fig. 2b). To further determine whether TRMT6/TRMT61A exerted function through its enzymatic activity, we generated *TRMT6*^*R377L*^/*TRMT61A*^*D181A*^ mutant construct. Since R377 of TRMT6 is essential for its binding with tRNA backbone and D181 of TRMT61A is responsible for SAM catalysis, mutation of these two sites abolished the enzymatic activity^28^. Co-transfection of wild type (WT) or mutant-*TRMT6/TRMT61A *in TRMT6/TRMT61A depleted CSCs restored protein levels of TRMT6 and TRMT61A (Fig. 2b). However, WT-TRMT6/TRMT61A but not *TRMT6*^*R377L*^/*TRMT61A*^*D181A*^ mutant (Mut) overexpression in TRMT6/TRMT61A depleted CSCs could restore signals of m1A (Fig. 2c, d), indicating that TRMT6/TRMT61A directly regulate RNA m^1^A modification in HCC CSCs. In addition, TRMT6/TRMT61A depletion did not affect several other tRNA modifications in liver CSCs (Supplementary Fig. 2j). Consistently, WT-TRMT6/TRMT61A overexpression could rescue oncosphere formation ability, whereas *TRMT6*^*R377L*^/*TRMT61A*^*D181A*^ mutant overexpression had no such effect (Fig. 2e). In addition, to test the potential in vivo correlation of these observations, we established xenograft models of TRMT6/TRMT61A depletion and/or introducing WT-TRMT6/TRMT61A or Mut-TRMT6/TRMT61A HCC cells. Notably, transplantation of TRMT6/TRMT61A depleted CSCs led to reduced CSC ratios (Supplementary Fig. 2k) and ability to form secondary tumors and serial xenograft tumors (Fig. 2f). *TRMT6/TRMT61A* depletion reduced tumor growth of orthotopic xenografts (Fig. 2g). Notably, Mut-*TRMT6/TRMT61A* overexpression could not rescue TRMT6/TRMT61A enhanced tumor growth (Fig. 2g). Consistently, although forced expression of Mut-TRMT6/TRMT61A could increase the protein levels, it could not enhance liver CSC self-renewal in vitro (Supplementary Fig. 2l, m). Together, these data indicate that m^1^A positive liver CSCs are dependent on the methylation activity of TRMT6/TRMT61A complex. TRMT6/TRMT61A-mediated m^1^A methylation is sufficient and necessary for liver CSC self-renewal and hepato-oncogenesis.

### m^1^A methylation of tRNA promotes PPARδ translation

Having demonstrated that global m^1^A levels were elevated in liver CSC cells, we then sought to interrogate differentially methylated tRNAs. To this end, we established a single-nucleotide resolution quantitative detection method. Previous methods coupling m^1^A antibody-mediated pre-enrichment step with m^1^A-induced misincorporation can detect m^1^A at single-nucleotide resolution in a transcriptome-wide manner^9^. However, in these methods, the stoichiometry information is lost due to the pre-enrichment step. Hence, we modified the existing methods to preserve the stoichiometry information of m^1^A58 in tRNA. To avoid over-representation of 18S-rRNA and 28S-rRNA reads in sequencing library, we firstly isolated small RNA fraction (<200 nt) from total RNA using a size selection step to enrich tRNA. Since both the abundance and m^1^A58 methylation levels of tRNAs are high, we performed a ligation-based strand-specific library construction directly without m^1^A immunoprecipitation. In addition, to distinguish signals caused by m^1^A from other false signals such as SNP and other modifications, we employed an in vitro demethylation step to provide a cleaner background. Such immunoprecipitation-free strategy hence enabled us to evaluate the m^1^A stoichiometry directly from the mismatch rate observed from sequencing data.

**Fig. 3:**
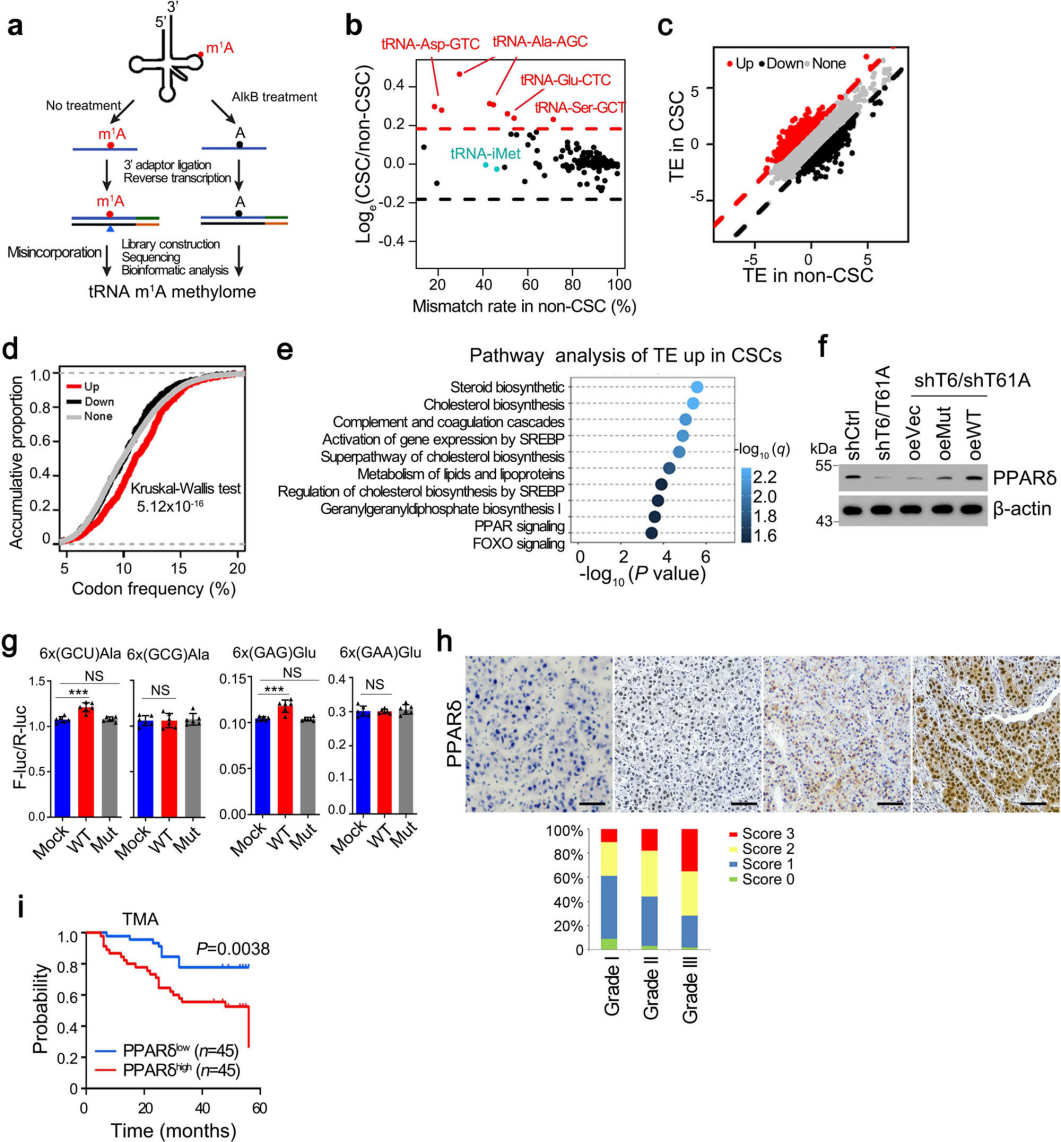
m^1^A58 methylation in tRNA regulates PPARδ translation. **a** Scheme of tRNA m^1^A-seq. **b** Comparison of misincorporation signals caused by m^1^A58 in tRNAs between liver CSC and non-CSC cells. Red dots represent tRNAs with elevated m^1^A58 methylation levels in CSCs. **c** Comparison of translation efficiency (TE) between liver CSC and non-CSC cells. Red dots represent genes with increased TE, while black dots represent genes with decreased TE in liver CSCs. **d** Frequency of codons corresponding to m^1^A58 elevated tRNAs was higher in the genes with increased translation efficiency (TE) in CSCs. **e** Unbiased pathway analysis of TE upregulated genes in liver CSCs compared with non-CSCs. **f** Western blotting analysis of PPARδ expression in indicated CSCs. *n* = 4 biologically independent samples. **g** Effect of elevated m^1^A58 methylation levels in tRNA^Ala(AGC)^ and tRNA^Glu(CTC)^ on protein synthesis. Reporter vectors were transfected into Mock overexpressed PLC/PRF/5 cells, WT-TRMT6/TRMT61A or Mut-TRMT6/TRMT61A overexpressed PLC/PRF/5 cells, respectively. Control vectors were also transfected into these three cell lines to normalize translation differences among these cell lines. The expression level of two synonymous codon control Ala-(GCG) and Glu-(GAA) remained unchanged upon TRMT6/TRMT61A expression level manipulation. *n* = 6 biologically independent samples. Exact *P* values from left to right: 0.00021, 0.27, 0.45, 0.00086, 0.21, 0.15. **h** Representative immunohistochemistry images of PPARδ (brown) in our cohort of 191 HCC samples. *n* = 3 independent samples. Scores 0 represents PPARδ staining that was considered negative, Scores 1 represents PPARδ staining that was considered negative, whereas scores 2 and 3 represent PPARδ staining that was considered positive. Grade I (*n* = 56), Grade II (*n* = 87), Grade III (*n* = 48). Scale bar, 100 μm. **i** Kaplan–Meier plots of overall survival for PPARδ in TMA cohort. Median expression value was used as a cut-off. *P* values for Kaplan–Meier curves were determined using a two-sided log-rank test. ***P < 0.001, and NS not significant (*P* > 0.05) by two-tailed Student’s *t*-test.

We then employed tRNA-m^1^A-seq to examine alteration of m^1^A levels between CSCs and non-CSCs in terms of their tRNA methylome (Fig. 3a). As expected, misincorporation signals of m^1^A58 in tRNAs responded quantitatively to demethylation treatment^29^ (Supplementary Fig. 3a). The misincorporation signal of this site basically disappeared upon AlkB demethylation treatment, demonstrating that such misincorporation signals are caused by m^1^A and hence can be used to evaluate methylation levels. Interestingly, we found that tRNAs with high m^1^A58 methylation levels did not show apparent modification difference between CSCs and non-CSCs. And for several tRNAs harboring medium levels of methylation, they tended to have higher m^1^A levels in CSCs. Using this strategy, we identified four differentially methylated tRNAs, including tRNA-Asp-GTC, tRNA-Ala-AGC, tRNA-Glu-CTC, and tRNA-Ser-GCT (Fig. 3b). In addition, it is worth mentioning that other tRNA modifications including m^1^G and m^3^C remained unchanged, indicating the specificity of m^1^A methylation in this process (Supplementary Fig. 3b). We also showed that global tRNA levels in CSCs and non-CSCs had comparable levels (~99.5% vs. ~99.6%). Moreover, expression level of individual tRNA between CSCs and non-CSCs was similar (Supplementary Fig. 3c), including the tRNA species with elevated m^1^A58 level. These results indicate that the regulatory process may take place on the level of tRNA m^1^A modification but not tRNA expression level.

To further identify the transcripts whose translation were regulated by these m^1^A58 elevated tRNAs, we performed ribosome profiling sequencing (Ribo-seq)^30,31^ analysis in CSCs and non-CSCs, as well as in TRMT6/TRMT61A depleted and control CSCs. We first checked the quality of our Ribo-seq data. Sequencing reads that were aligned to coding regions of mRNA were mostly 28–32 nucleotides, in line with ribosome protected fragment sizes (Supplementary Fig. 3d). The offsets between the 5′ end of the footprint and the P-site codons at translation initiation and termination, as well as the 3-nucleotide periodicity, were also readily observed in our data (Supplementary Fig. 3e, f). With Ribo-seq data, we found a subset of genes demonstrating higher TE in liver CSCs (Fig. 3c). Intriguingly, this set of genes with elevated TE were composed of a relatively enriched codons corresponding to m^1^A-elevated tRNAs (Fig. 3d), further confirming that m^1^A58 promotes translation elongation. To identify candidate targets of the TRMT6/TRMT61A-m^1^A axis, we focused on genes whose TE were upregulated in CSCs, but downregulated with *TRMT6/TRMT61A* depletion through Ribo-seq analysis. We found that 569 co-regulated genes contained 161 transcription factors (TFs) (Supplementary Fig. 3g). We then selected top ten TFs with substantial changes in translation efficiency (TE). Among top ten TFs, only PPARδ showed significantly upregulated protein but not mRNA level in liver CSCs (Supplementary Fig. 3h, i). In addition, we performed pathway analyses of these TE upregulated genes in CSCs and found they were enriched in pathways including steroid biosynthesis, cholesterol biosynthesis, lipid metabolism, and PPAR signaling pathway (Fig. 3e). We further performed quantitative proteomic analysis after tandem mass tag (TMT) labeling using control and TRMT6/TRMT61A depleted CSCs. Gene ontology (GO) analysis of these proteins downregulated in TRMT6/TRMT61A depleted CSCs enriched in metabolic, PPAR, and lipid metabolic pathways (Supplementary Fig. 3j).

Peroxisome proliferator-activated receptors (PPARs) are ligand-activated transcription factors. Three PPAR isoforms, PPARα, PPARδ, and PPARγ, have been identified in all mammalianspecies^32^. PPARδ regulates glucose and lipid homeostasis^33,34^. However, functions of PPARδ in tumourigenesis still remain elusive. Of note, *PPARD* mRNA expression levels showed no significant change in liver CSCs compared with *PPARA* and *PPARG* (Supplementary Fig. 4a), whereas its protein levels increased in liver CSCs (Supplementary Fig. 4b). Moreover, we found that *TRMT6/TRMT61A* depletion reduced PPARδ protein expression (Fig. 3f), but not *PPARD* mRNA (Supplementary Fig. 4c). WT-TRMT6/TRMT61A overexpression in TRMT6/TRMT61A depleted CSCs could restore PPARδ expression levels (Fig. 3f), whereas Mut-TRMT6/TRMT61A overexpression had no such effect (Fig. 3f). To define how m^1^A methylation regulated PPARδ expression level, we first analyzed codon frequency of *PPARD* mRNA. Consistently, we found that *PPARD* mRNA was particularly enriched in AGC and GAG codons (top 3.5%, AGC; top 7%, GAG), whose corresponding tRNAs displayed higher m^1^A methylation levels in liver CSCs (Supplementary Fig. 4d), suggesting that PPARδ translation may be regulated by elevated tRNA m^1^A methylation in liver CSCs. Since m^1^A58 in tRNA was reported to promote translation^12^, we envisioned that tRNAs with elevated m^1^A levels in CSCs might have higher translation efficiency (TE). We first employed a dual-luciferase reporter system (Supplementary Fig. 4e) to confirm that those tRNAs with elevated m^1^A levels also promoted translation in liver cancer cell lines. Specifically, we inserted a six-codon repeated sequence immediately before firefly luciferase (*F-luc*) and the second renilla luciferase (*R-luc*) and used this vector as a transfection control. Additionally, we utilized a control vector without codon insertion to normalize the inherent translation difference (Supplementary Fig. 4e). We used TRMT6/TRMT61A overexpressed cells to mimic CSCs. And we found that TRMT6/TRMT61A overexpression enhanced translation levels, but not their mutant counterpart (Fig. 3g), indicating that the translation-promoting regulation is dependent on m^1^A58 modification rather than other functions of the complex.

In addition, PPARδ was highly expressed in 40–72% HCC tumor tissues among our 191 HCC samples (Fig. 3h). High levels of PPARδ were correlated with poor survival rates in the TMA cohort (Fig. 3i). Finally, PPARδ depletion reduced the ability of liver CSCs self-renewal in vivo (Supplementary Fig. 4f). Consistently, PPARδ antagonist GSK3787 also reduced liver CSC self-renewal in vitro (Supplementary Fig. 4g). Together, these data indicate that TRMT6/TRMT61A-mediated m^1^A modification enhances PPARδ protein translation, and PPARδ promotes liver CSC self-renewal and tumor growth.

### PPARδ augments cholesterol biosynthesis

**Fig. 4:**
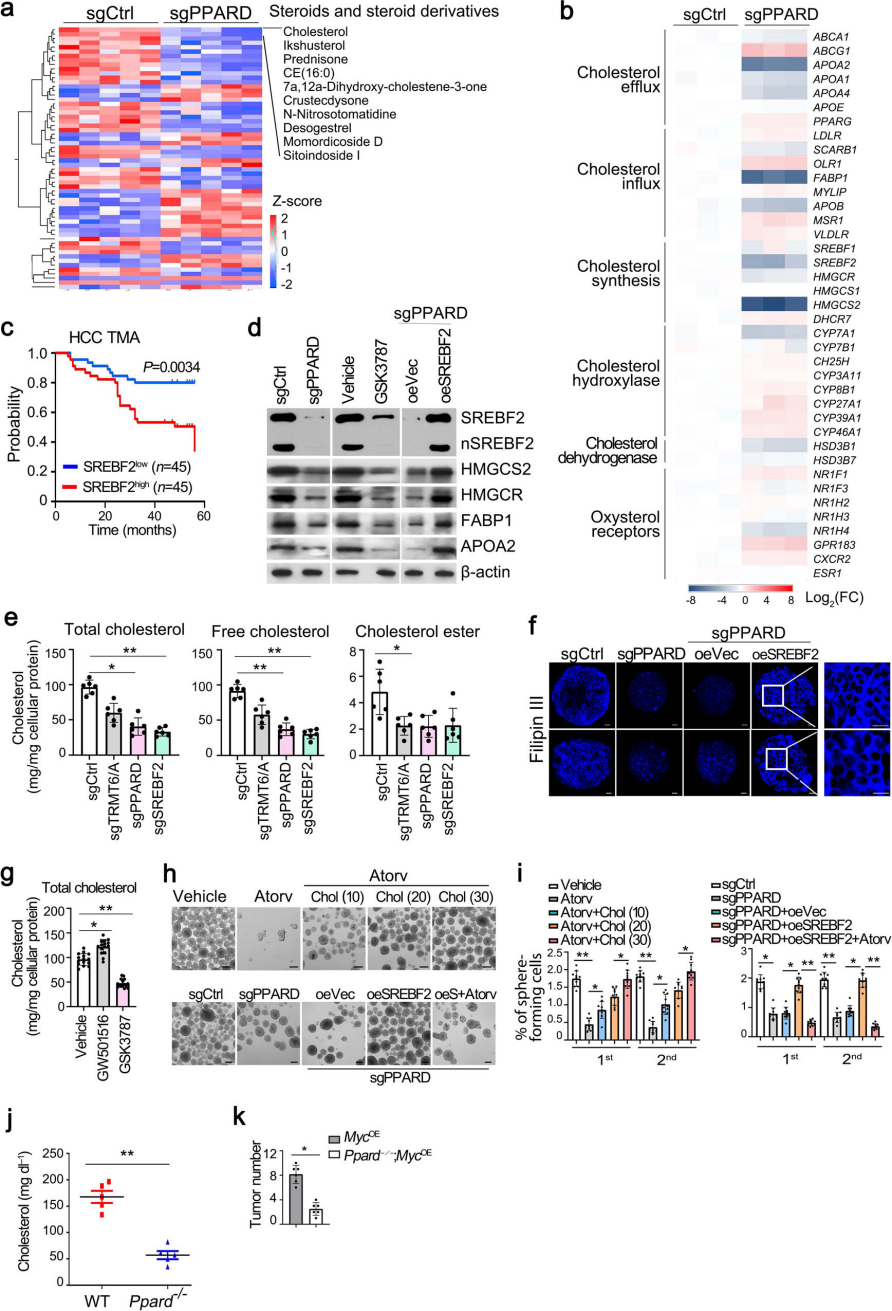
PPARδ promotes liver CSC self-renewal via enhancing cholesterol synthesis. **a** Heatmap analysis of signature molecules of steroid metabolites under positive ion mode in PPARδ depleted versus sgCtrl treated liver CSC cells. **b** Heatmap analysis of cholesterol metabolism related genes in PPARδ-depleted and control CSCs. Data were normalized to endogenous 18S rRNA expression and sgCtrl were assigned with a value of 1. **c** Kaplan–Meier plots of overall survival for SREBF2 in TMA cohort. Median expression value was used as a cut-off. *P* values for Kaplan–Meier curve were determined using a two-sided log-rank test. **d** Western blotting analysis of cholesterol metabolism related gene expression in PPARδ depleted, GSK3787 (20 μM) treated and SREBF2 overexpressed liver CSCs compared with control CSCs. *n* = 3 biologically independent samples. **e** Levels of total cholesterol, free cholesterol and cholesterol esterin TRMT6/TRMT61A, PPARδ, and SREBF2 depleted liver CSCs. Data are means ± SD. *n* = 6. Exact *P* values from left to right: 0.037, 0.0061, 0.0070, 0.0032, 0.046. **f** Filipin III staining of cellular cholesterol distributions in PPARδ depleted, and co-transfection of SREBF2 in PPARδ depleted liver CSCs from two HCC patients compared with control CSCs (*n* = 5). Scale bar, 10 μm. *n* = 3 biologically independent samples. **g** Total cholesterol levels in liver CSCs treated with vehicle (DMSO), PPARδ antagonist (GSK3787, 20 μM) or agonist (GW501516, 20 μM) for 72 h. Data are means ± SD. Exact *P* values from left to right: 0.041, 0.0073. CSCs were sorted from HCC patients, *n* = 15. **h**, **i** Representative images of oncosphere formation analysis from liver CSCs treated with vehicle, cholesterol synthesis inhibitor atorvastatin (atorv, HMGCR inhibitor, 5 μM), cholesterol (20, 30, and 40 μM) (upper panel) (**h**). Oncosphere formation analysis of PPARδ-depleted spheres treated with overexpression of SREBF2, coupling with atorvastatin (atorv) (lower panel) (**i**). Scale bar, 100 μm. Liver CSCs were sorted from HCC primary samples, *n* = 10. Data are means ± SD. Exact *P* values from left to right: 0.0032, 0.047, 0.046, 0.0019, 0.026, 0.038; 0.026, 0.037, 0.0065, 0.0091, 0.043, 0.0029. **j** Total serum cholesterol levels in 10-week-old normal-fed male *Myc*^OE^ mice transduced with sgPpard using CRISPR/Cas9 strategy for 10 days (*Ppard*^−/−^; *Myc*^OE^). WT:*Ppard*^+/+^; *Myc*^OE^. Data are means ± SD. *n* = 5 mice per group. Exact *P* value: 0.0073. These experiments were repeated three times. **k** Tumor numbers from *Ppard*^+/+^; *Myc*^OE^ and *Ppard*^-/-^; *Myc*^OE^ mice. Data are shown as means ± SD (*n* = 6 mice per group). Exact *P* value: 0.033. *P < 0.05; **P < 0.01 by two-tailed Student’s *t*-test.

To explore the mechanism by which PPARδ promoted liver tumourigenesis, we performed genome-wide transcriptional profiling between PPARδ depleted and sgCtrl CSCs. We found that genes downregulated in PPARδ-depleted liver CSCs were strongly enriched in lipid metabolic and steroid metabolic processes (Supplementary Fig. 5a). Many tumors harbor aberrantly activated lipid metabolism to aid their growth^15,16^. Phenotypic and metabolic heterogeneity within tumors is a major barrier to effective cancer therapy^35,36^. We then performed untargeted lipidomic chromatography-mass spectrometry (LC-MS)-based metabolite profiling analysis to identify metabolites involved in this process (Supplementary Fig. 5b, c). Metabolite set enrichment analysis (MSEA) ranked steroid biosynthesis as a top pathway in PPARδ-depleted liver CSCs (Supplementary Fig. 5d). Of note, the most robustly downregulated molecule was cholesterol, whose intensity dropped by 95% compared with control cells (Fig. 4a). As expected, cholesterol synthetic genes *SREBF2*, *HMGCR*, and *HMGCS2* were remarkably downregulated in PPARδ-depleted liver CSCs (Fig. 4b and Supplementary Fig. 5e). Notably, they were highly expressed in liver tumors in the TCGA dataset (Supplementary Fig. 5f) and our cohort (Supplementary Fig. 5g). In addition, SREBF2 expression was negatively correlated with overall survival of HCC patients in the TMA cohort (Fig. 4c). We observed that mature SREBF2 markedly decreased in PPARδ-depleted liver CSCs and PPARδ antagonist GSK3787 treated liver CSCs (Fig. 4d). In addition, PPARδ deficiency promoted degradation of SREBP2 protein (Supplementary Fig. 5h).

Besides SREBF2, four other key proteins related to cholesterol homeostasis—including HMGCS2 (synthesis), FABP1 (uptake), HMGCR (synthesis), and APOA2 (efflux), were also downregulated in PPARδ-depleted liver CSCs (Fig. 4d), suggesting that PPARδ may regulate cholesterol homeostasis in liver CSCs.

In agreement with the reduced cholesterol biosynthetic gene expression, free cholesterol content was decreased by 60% in PPARδ deficient CSCs compared to controls (Fig. 4e). TRMT6/TRMT61A or SREBF2 silencing also reduced cholesterol levels (Fig. 4e). Cholesterol is the most abundant sterol in the plasma membrane, and more than 80% cellular cholesterol is distributed on the plasma membrane, and membrane cholesterol exerts important functions in signaling transduction^17^. Expectedly, PPARδ depletion remarkably reduced levels of membrane cholesterol of liver CSCs (Fig. 4f). Consistently, PPARδ agonist GW501516 increased cholesterol levels, whereas its antagonist GSK3787 decreased cholesterol levels in liver CSCs from HCC samples (Fig. 4g). To determine whether there might be a functional relationship between cholesterol biosynthesis and liver CSCs self-renewal, we first tested whether increasing cholesterol content could rescue the phenotype in PPARδ deficient CSCs. Indeed, suppression of cholesterol biosynthesis by low dose atorvastatin (Atorv, an HMGCR inhibitor; 10 μM) treatment inhibited ability of oncosphere formation (Fig. 4h, i). Importantly, cholesterol was able to promote CSC self-renewal ability in a dose-dependent manner (Fig. 4h, i), confirming that cholesterol is involved in liver CSC self-renewal. In parallel, SREBF2 overexpression in PPARδ-depleted CSCs could restore oncosphere formation ability, whereas atorvastatin treatment further decreased oncosphere formation capacity (Fig. 4h, i). To further determine the role of PPARδ in cholesterol biogenesis and tumourigenesis in vivo, we next deleted *Ppard* in *Myc*^OE^ mice using CRISPR/Cas9-mediated genome editing in vivo^37^ (Supplementary Fig. 5i). We found that *Ppard*^*−/−*^ mice dramatically reduced serum cholesterol levels (Fig. 4j), cholesterol biogenesis and uptake-related gene expression (Supplementary Fig. 5i). Consequently, *Ppard* deficiency remarkably inhibited tumor development in *Myc*^OE^ mice (Fig. 4k). Collectively, PPARδ induced cholesterol biogenesis promotes liver CSC self-renewal.

### PPARδ-mediated cholesterol biogenesis initiates Hedgehog signaling

**Fig. 5:**
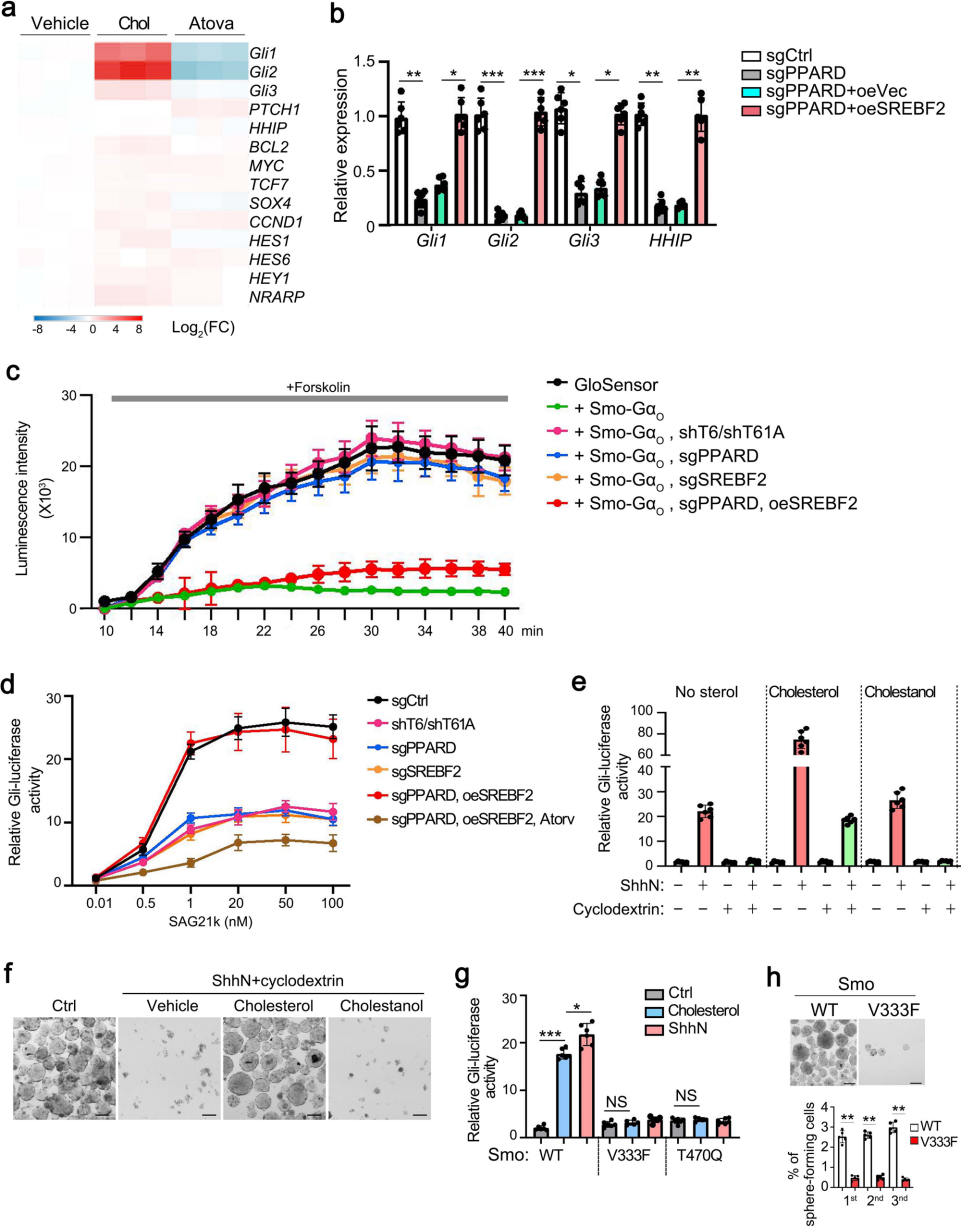
Cholesterol activates Hh signaling in liver CSCs. **a** Indicated genes of stemness related signaling pathways were analyzed by qRT-PCR in vehicle (DMSO), cholesterol (Chol), and cholesterol biosynthesis inhibitor atorvastatin (Atorv, an HMGCR inhibitor) treated liver CSCs. **b** Hh signaling genes were analyzed in PPARδ depleted and in forced expression of SREBF2 CSCs. Data were normalized to endogenous 18S rRNA expression and sgCtrl were assigned with a value of 1. Data are means ± SD. *n* = 5. Exact *P* values from left to right: 0.0059, 0.026, 0.00018, 0.00035, 0.021, 0.032, 0.0037, 0.0045. **c** Live-cell luminescence traces from liver CSC cells introduced with indicated plasmids. Baseline luminescence was recorded for 10 min, followed by forskolin treatment and continued detecting at 2 min intervals. *n* = 5. **d** Liver cancer cells were transfected with Gli-luciferase (Gli-Luc) reporter plasmids, followed by introduced by indicated plasmids. Luciferase activity was measured following treatment with increasing concentrations of Smo agonist SAG21k. *n* = 5. **e** Gli-luciferase activity liver cancer cells stimulated with ShhN-conditioned medium in combination with exogenous cholesterol (50 μM) or cholestanol (50 μM) following endogenous sterol depletion by cyclodextrin. *n* = 6. **f** Sphere formation assay of cells from **e**. Scale bar, 100 μm. *n* = 4 biologically independent samples. **g** Gli-Luc signaling assay of WT *Smo* or *Smo* mutations (V333F or T470Q) at deep 7TM in liver cancer cells stimulated with ShhN-conditioned medium in combination with exogenous cholesterol (50 μM). Data are means ± SD. *n* = 5. Exact *P* values from left to right: 0.00092, 0.041, 0.058, 0.14. **h** Sphere formation assay of cells transfected with WT-*Smo* or Mut-*Smo*. Graph data are means ± SD. *n* = 5 biologically independent samples. Exact *P* values from left to right: 0.0039, 0.0037, 0.0020. *P < 0.05; **P < 0.01; ***P < 0.001, and NS not significant (*P* > 0.05) by two-tailed Student’s *t*-test.

Aberrant Hedgehog (Hh) signaling is implicated in several malignancies^38,39^. Smo is a seven-transmembrane (7TM) oncoprotein and plays a major role in Hh signaling transduction. Once activated, Smo subsequently activates glioma-associated (Gli) transcription factors^40^. Cholesterol plays a critical role in Smo activation^41^. We found that cholesterol dramatically promoted Hh signaling activation (Fig. 5a). In parallel, *PPARD* depletion remarkably downregulated *Gli* expression, whereas SREBF2 overexpression was able to restore *Gli* expression levels (Fig. 5b). G protein coupling used as a robust detection of Smo activity accurately provides status of the active and inactive Smo that are likely physiologically coupled to *Gli* transcription factors. Of note, TRMT6/TRMT61A, PPARδ, or SREBF2 deletion decreased Smo activity, whereas SREBF2 overexpression restored the activity of Smo through the GloSensor cAMP assay^42^ (Fig. 5c). In addition, we observed that Hh pathway activation by Smo agonist SAG21k was impaired in TRMT6/TRMT61A, PPARδ, or SREBF2 depleted liver CSCs, while atorvastatin could block the function of SREBF2 (Fig. 5d). Intriguingly, exogenous cholesterol but not cholestanol (an isomer of cholesterol) enhanced Hh signaling activation in overexpression of N-terminal signaling domain of Sonic Hedgehog (ShhN) in liver CSCs (Fig. 5e). When we removed sterol by cyclodextrin in liver CSCs, the function of ShhN on Hh signaling activation was blocked (Fig. 5e). Consequently, exogenous cholesterol coupling with ShhN could promote survival of sterol-depleted liver CSCs (Fig. 5f). A recent structural study showed that Smo harbors a cholesterol molecule binding within a deep 7TM site^43^. We therefore examined whether cholesterol effects were altered on mutation of the 7TM site (SMO V333F or T470Q). Cholesterol could induce *Gli* activation similar to ShhN (Fig. 5g). Mutations against the 7TM sites (Smo V333F and T470Q) caused a significant loss of cholesterol-induced *Gli* activation (Fig. 5g). Consequently, mutation of the cholesterol binding site in Smo inhibited liver CSC self-renewal in vitro (Fig. 5h). Collectively, these data suggest that cholesterol is required to activate Hh signaling pathway in liver CSCs.

### *Trmt6/Trmt61a* knockout abrogates cholesterol synthesis and inhibits liver tumourigenesis

**Fig. 6:**
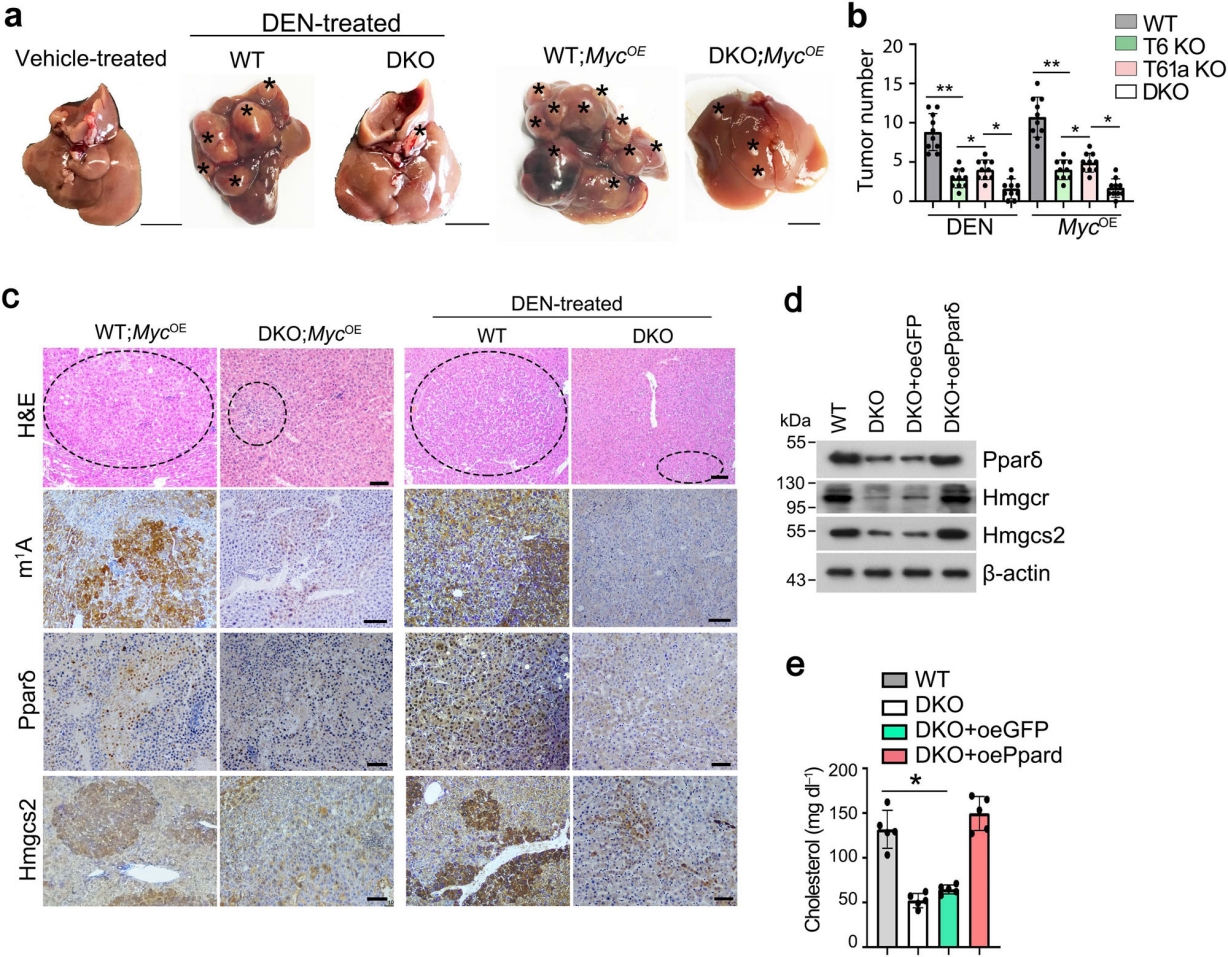
Trmt6/Trmt61a knockout inhibits mouse cholesterol synthesis through Pparδ pathway. **a** Macroscopic appearance of livers from *Trmt6*^f/+^*/Trmt61a*^f/+^; Alb-Cre (WT) and *Trmt6/Trmt61a *DKO (*Trmt6*^f/f^*/Trmt61a*^f/f^; Alb-Cre) mice under DEN-induced cancer or in *Myc*^OE^ mouse model of HCC. Black asterisks indicate liver tumors. Scale bar, 1 cm. **b** Quantitation of liver tumor numbers of *Trmt6* KO, *Trmt61a* KO, and *Trmt6/Trmt61a* DKO in DEN-treated and in *Myc*^OE^ mice. Results are shown as means ± SD (*n* = 10 biologically independent mice). Exact *P* values from left to right: 0.0075, 0.041, 0.032, 0.0037, 0.045, 0.014. **c** Representative liver H&E staining and immunohistochemistry images of m^1^A, Pparδ, and Hmgcs2 (brown) from *Trmt6*^*f/*+^*/Trmt61a*^*f/+*^ (WT) and *Trmt6*^−/−^*/Trmt61a*^−/−^ (DKO) in *Myc*^OE^ and DEN-treated mice. Black circle indicates tumor region. Scale bar, 100 μm. *n* = 3 independent samples. **d** Levels of cholesterol biogenesis related proteins in 10-week-old male *Trmt6/Trmt61a *deleted (DKO) in *Myc*^OE^ mice transduced with AAV vectors encoding GFP control (oeGFP) or Pparδ (oePparδ) for 8 days. WT: *Trmt6*^*f/+*^*/Trmt61a*^*f/+*^; *Myc*^OE^ mice. *n* = 5 biologically independent mice. **e** Total serum cholesterol levels in 10-week-old normal-fed male *Trmt6/Trmt61a* deleted *Myc*^OE^ (DKO) mice transduced with AAV vectors encoding GFP control (oeGFP) or Pparδ (oePparδ) for 8 days. WT: *Trmt6*^*f/+*^*/Trmt61a*^*f/+*^; *Myc*^OE^ mice. Data are means ± SD. *n* = 5 mice per group. Exact *P* values: 0.021. These experiments were repeated three times. *P < 0.05; **P < 0.01 by two-tailed Student’s *t*-test.

To further determine the physiological role of TRMT6/TRMT61A complex in tumourigenesis in vivo, we then generated *Trmt6*^flox/flox^ and *Trmt61a*^flox/flox^ mice by insertion of loxP sequences flanking exon 2 of *Trmt6* locus and flanking exon 1 of *Trmt61a* locus through CRISPR/Cas9 technology, respectively (Supplementary Fig. 6a, b). Next, we deleted *Trmt6* and/or *Trmt61a* in hepatocytes by crossing *Trmt6*^f/f^ and/or *Trmt61a*^f/f^ mice with Alb*-*Cre mice (Supplementary Fig. 6a, b). Trmt6/Trmt61a knockout significantly decreased m^1^A levels in liver tissues (Supplementary Fig. 6c), whereas did not affect several other tRNA modifications (Supplementary Fig. 6d). We found that *Trmt6*^−/−^, *Trmt61a*^−/−^, or double knockout (DKO) mice remarkably suppressed development of mouse liver cancers with DEN treatment compared to littermate control mice (Fig. 6a, b). Parallelly, DKO in *Myc*^OE^ mice also displayed suppression of liver cancer development compared to littermate control mice (Fig. 6a, b).*Trmt6/Trmt61a* DKO reduced levels of m^1^A, Pparδ, and Hmgcs2 in *Myc*^OE^ or DEN induced liver tumor tissues (Fig. 6c).

To further verify the function of Trmt6/Trmt61a-Pparδ axis in vivo, we transduced *Trmt6/Trmt61a *DKO in *Myc*^OE^ mice with adeno-associated viral vectors (AAV) encoding green fluorescent protein (GFP) control or Pparδ (Fig. 6d). Finally, Pparδ overexpression in *Trmt6/Trmt61a* deleted *Myc*^OE^ mice dramatically increased levels of cholesterol synthesis-related genes (Fig. 6d) and serum cholesterol levels in *Myc*^OE^ mice (Fig. 6e). Altogether, the Trmt6/Trmt61a*-*m^1^A promotes mouse liver cancer development.

### m^1^A methylation inhibitor suppresses self-renewal of liver CSCs and liver oncogenesis

**Fig. 7:**
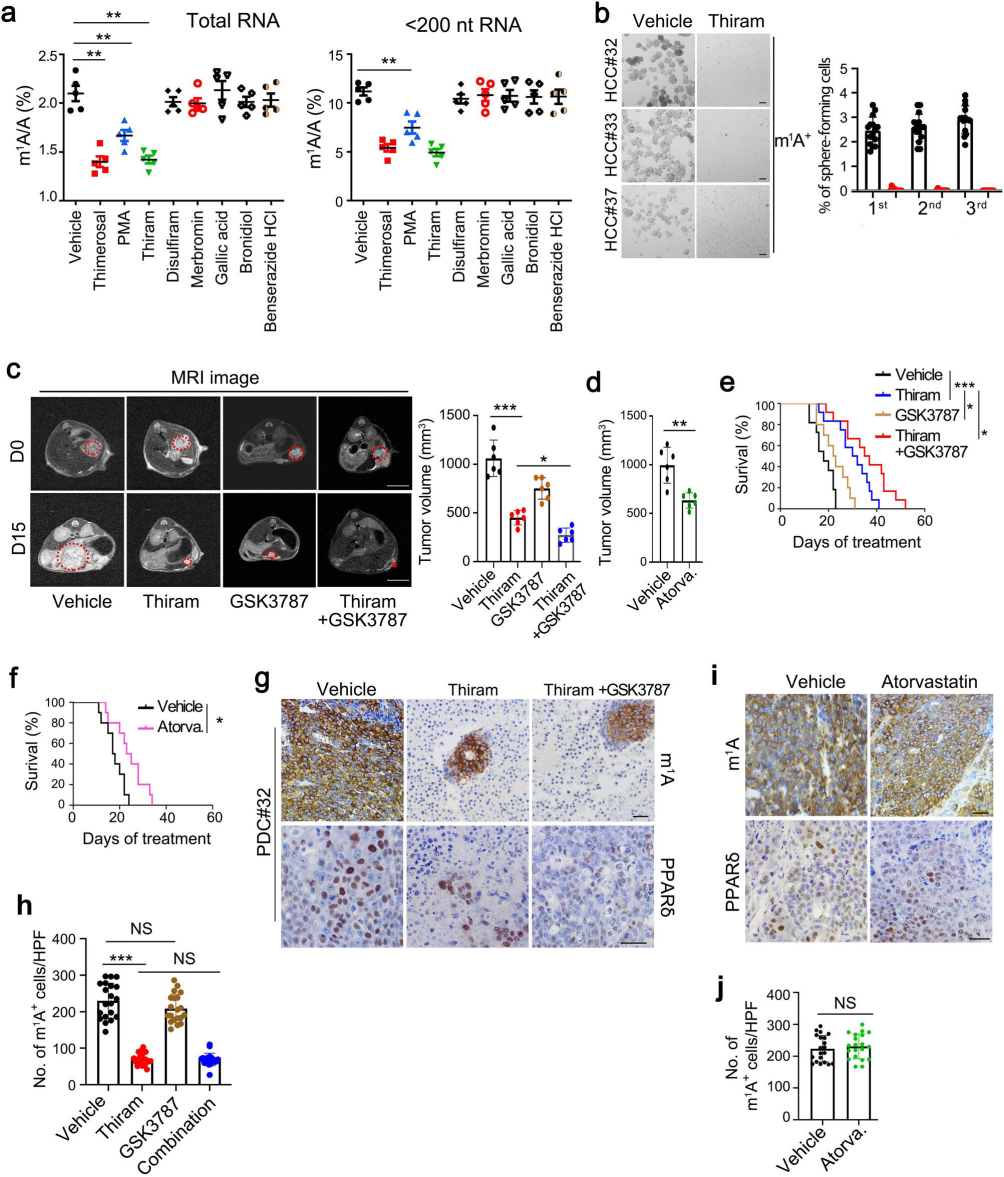
TRMT6/TRMT61A complex inhibitor thiram suppresses self-renewal of liver CSCs and tumor growth. **a** Quantitative LC-MS/MS analysis of m^1^A/A in total RNA and tRNA purified from PLC/PRF/5 cells treated with vehicle or drugs at IC50. Statistical results as means ± SD, *n* = 5 biologically independent samples. Exact *P* values from left to right: 0.0048, 0.0086, 0.0037, 0.0090. **b** Oncosphere forming assays of HCC primary cells following thiram or vehicle treatment. Scale bar, 100 μm. Right panel represents statistical results as means ± SD. *n* = 15 independent experiments. **c** Representative magnetic resonance imaging (MRI) images of independent experimental cohorts, of day 0 and day 15 of mice enrolled in treatment with vehicle (DMSO), thiram (1.6 mg kg^−1^), GSK3787 (5 mg kg^−1^) or thiram combined with GSK3787. Red circles and asterisk indicate visible tumor area that was used to calculate tumor volumes. Scale bar, 1 cm. Tumor volumes were calculated on the basis of MRI images from mice bearing liver tumor with a matched initial tumor volume (Right panel). Graph shows means ± SD. *n* = 6 mice per group. Exact *P* values from left to right: 0.00065, 0.028. **d** Tumor volumes were calculated from mice treated with vehicle or atorvastatin (Atorva., 10 mg kg^−1^). Graph shows means ± SD. *n* = 6 mice per group. Exact *P* value: 0.0073. **e** Survival curve derived from mice bearing HCC PDC tumors, treated with vehicle (*n* = 11), thiram (*n* = 12), GSK3787 (*n* = 10), or thiram combined with GSK3787 (*n* = 12). *P* values for Kaplan–Meier curves were determined using a two-sided log-rank test. Exact *P* values from up to down: 0.00011, 0.025, 0.018. **f** Survival curve derived from mice bearing HCC PDC tumors, treated with vehicle (*n* = 10) and atorvastatin (*n* = 10). *P* value for Kaplan–Meier curves were determined using a two-sided log-rank test. Exact *P* value: 0.011. **g**, **i** Representative immunohistochemical images of m^1^A and PPARδ levels in PDC orthotopic engraft mouse models treated with vehicle, thiram, GSK3787, thiram combined with GSK3787 (combination) or atorvastatin (Atorva.). Scale bar, 100 μm. **h**, **j** Graphs of numbers of m^1^A positive (m^1^A^+^) cells per tumor nodule after treatment. Data are means ± SD. *n* = 20. Exact *P* values from left to right: <0.0001, 0.13, 0.85 (**h**); and 0.58 (**j**). *P < 0.05; **P < 0.01; ***P < 0.001; NS not significant (*P* > 0.05) by two-tailed Student’s *t*-test.

Liver cancer still remains hard to treat because of lack of drugs against effective targets^4^, addressing an urgent need to develop new therapeutic measures. Based on our data above, we proposed that the TRMT6/TRMT61A complex may be a potential therapeutic target for HCC. Since m^1^A modification on RNA is mediated by the TRMT6/TRMT61A complex, preventing the interaction of TRMT6 with TRMT61A indicates blocking the formation of TRMT6/TRMT61A complex to abrogate m^1^A modification in tRNA. We then screened potential inhibitors blocking TRMT6/TRMT61A interaction from a FDA-approved drug bank containing 1600 molecules. We found 8 candidates could potentially block the interaction of TRMT6 and TRMT61A in vitro. Among them, thimerosal, phenylmercuric acetate (PMA), thiram, and disulfiram dramatically suppressed proliferation of HCC cell lines compared with other candidates (Supplementary Fig. 7a–e). Consistently, the anti-proliferative effects of thimerosal, phenylmercuric acetate (PMA) and thiram were associated with the reduction of m^1^A levels in HCC cells (Fig. 7a). Thiram treatment could dramatically decrease m^1^A levels compared with vehicle treatment. Moreover, thiram treatment did not affect other RNA modification levels including m^1^G, and Ψ (Supplementary Fig. 7f), which were similarly with TRMT6/TRMT61A depletion results. In addition, thiram could remarkably suppressed oncosphere formation in HCC primary cells and HCC cell lines (Fig. 7b). Anti-tumourigenic abilities of thiram were further validated by orthotopic implantation with patient-derived tumor cells (PDC) into NSG mice. Thiram was injected intraperitoneally into 12 PDC orthotopic xenograft models of HCC at a dose of 1.6 mg kg^−1^ day^−1^. Thiram markedly inhibited xenograft tumor growth of PDC cells compared with GSK3787 alone. Importantly, combination of thiram with PPARδ antagonist GSK3787 had synergic effect on tumor suppression (Fig. 7c). Atorvastatin treatment also decreased xenograft tumor growth (Fig. 7d). Thiram and GSK3787 combination significantly increased mouse survival rates than thiram, GSK3787, or atorvastatin treatment (Fig. 7e, f). In addition, thiram treatment could decrease numbers of m^1^A positive cells (Fig. 7g, h), suggesting that HCC cells are efficiently eliminated by treatment with thiram. Consistently, thiram treatment reduced expression levels of PPARδ (Fig. 7g) and subsequent cholesterol synthesis (Supplementary Fig. 7g). However, atorvastatin treatment could not reduce the numbers of m^1^A positive cells or levels of PPARδ (Fig. 7i, j). Altogether, thiram acts as an efficient inhibitor against TRMT6/TRMT61A complex that effectively inhibits tumor growth of liver cancer.

## Discussion

HCC accounts for the majority of primary liver cancers and is characterized by high recurrence and heterogeneity. Heterogeneity is mainly caused by a hierarchical organization of CSCs^5^. m^1^A at position 58 (m^1^A58) in tRNA is an essential and highly conserved modification for several physiological processes^44^. However, the functions of m^1^A-methylations in RNA of cancers remain largely unknown. Here we reveal that m^1^A methylation levels are correlated with HCC progression and survival. We demonstrate that endogenous m^1^A methylation exerts a critical role in HCC development and liver CSC self-renewal. We show m^1^A methyltransferase complexTRMT6/TRMT61A are elevated in HCC tumor tissues and liver CSCs. TRMT6/TRMT61A-mediated m^1^A methylation is sufficient and necessary for liver tumourigenesis and self-renewal of liver CSCs. Mechanistically, TRMT6/TRMT61A enhance PPARδ translation, which initiates cholesterol synthesis, leading to Hh signaling activation. Finally, we identify an effective inhibitor thiram against TRMT6/TRMT61A complex, which remarkably suppresses self-renewal of liver CSCs and liver tumor growth.

Liver CSCs can drive hepatobiliary carcinogenesis and be a source of tumor initiation. Cellular reprogramming of differentiated cells into a more pluripotent state plays an essential role in the tumourigenesis of HCC^45,46^. Early HCC were characterized by activation of genes^47^ or proteins^19^ involved in metabolic functions. However, little is known about metabolic reprograming in liver CSC self-renewal and tumourigenesis. Herein, we reveal that genes with high TE in liver CSCs are enriched in steroid biosynthesis and lipid metabolism pathways. We show that TRMT6 positive cells are surprisingly more than m^1^A positive cells by histochemical staining. TRMT6 is elevated in 20% in well differentiated (Grade I), 20–40% in advanced HCC samples. However, in most cases of 191 HCC samples, m^1^A signal levels are less than 20% in HCC tumor tissues. Notably, m^1^A signal levels are highly elevated in liver CSCs. We conclude that tumor cells with extremely high m^1^A signals are liver CSCs. TRMT6 needs to be complex with its partner component TRMT61A, forming the TRMT6/TRMT61A complex to catalyze m^1^A methylation. TRMT6 alone or binding other TRMT member components generates other complexes to exert different functions. Given that liver CSCs harbor great heterogeneity, a bunch of surface markers have been identified up to date, thus CD13^+^CD133^+^ liver CSC populations we used could not encompass whole CSCs. Another reason could be intermediate states of liver CSCs exist; implying non-CSCs are mixed with the intermediate state populations of liver CSCs. In fact, pure CSCs have not been isolated by surface markers up to date. Thus TRMT6 positive cells could contain different subpopulations in HCC tumors and could harbor different features, which needs to be further investigation.

Translational regulation plays important roles in tumor development and progression. Several tRNA modifications have been reported to contribute to mRNA translation process, including translation efficiency and accuracy, and are further involved in the regulation of cancer development^48,49^. m^1^A58 modification is both linked to structural stability and/or correct folding of tRNA. m^1^A methylation occurs on a unique base since it prevents Watson-Crick base paring and introduces a positive charge^50^. In mRNAs, m^1^A mainly exists in highly structured 5′ UTRs, suggesting that it may alter predicted secondary structure^8^. Within loop structures, this charge may serve to stabilize interactions with the phosphate backbone of RNA^50^. mRNA translation is often regulated by activity of translation initiation factors and availability of ribosomes. Translational initiation step is the rate-limiting step. m^1^A58 modification is crucial for stability of initiator tRNA^Met^ in both yeast^51^ and human^12^, which thus promotes translation initiation. m^1^A58 modification in human tRNA^Lys^_3_ was reported to be an important for reverse transcription fidelity and efficiency of HIV^52^. Hyper modification at m^1^A58 site can alter association of certain tRNAs with polysomes^53^. m^1^A methylation results in increased translation of the transcript, perhaps due to access or direct recruitment of initiation and elongation factors^12^. The positive charge of this modification makes it amenable to specific protein–RNA interactions and unique RNA-RNA interactions. Here we reveal that m^1^A methylation levels are correlated with the grade of HCC and MVI status, suggesting that m^1^A levels may be used as a potential prognostic indicator. We show that other RNA modifications such as m^6^A, m^1^G and Ψ are not overtly changed in liver CSCs versus non-CSCs. In line with high m^1^A levels, TRMT6/TRMT61A complex are markedly upregulated in liver CSCs and tumors. However, ALKBH1, as the first tRNA m^1^A demethylase identified in human cells^12^, is not significantly changed between liver CSCs and non-CSCs. Our results indicate that the translation-promoting regulation is dependent on m^1^A58 modification rather than other functions of the TRMT6/TRMT61A complex. Of note, steroid biosynthesis, lipid metabolism, and PPAR signaling pathways are preferentially enriched in liver CSCs. Recent studies reported that TRMT6, TRMT61A, TRMT10C, and YTHDF1 might predict HCC patient survival using TCGA-LIHC dataset analysis. Based on bioinformatic analysis, they showed that the PI3K/Akt signaling pathway may be involved in upstream regulation of m^1^A in HCC^54^ or gastrointestinal cancer^55^.

PPARs are ligand-activated transcription factors^32^. Three PPAR isoforms, PPARα, PPARβ/δ (also known as PPARβ or PPARδ), and PPARγ, have been defined in all mammalian species up to date. PPARs influence many important biological processes, including inflammation^56^, cell survival^57^, and differentiation^58^. The role of PPARδ in carcinogenesis is controversial^59^. Several reports showed that PPARδ is upregulated in cancer cells by an adenomatous polyposis coli (APC)–β-catenin–TCF4 pathway that plays a pro-tumourigenic effect on many cancer types^60^. However, other studies showed that PPARδ agonists can induce terminal differentiation and inhibit innate inflammation, suggesting antitumour effects^61^. The PPARδ pathway is implicated in regulating ISC proliferation during high-fat diet feeding^34^. However, the role and mechanism of PPARδ in the regulation of liver CSC self-renewal and liver cancer development remain unclear. Herein we show that PPARδ is a pro-tumor factor in liver cancer development. PPARδis activated by endogenous ligands that are derived from the metabolism of fatty acids and other compounds in diet, which is consistent with the fact that PPARδ regulates expression of many genes involved in phosphatidylcholine metabolism^33^.

Phenotypic and metabolic heterogeneity within tumors is a major barrier to effective cancer therapy^24,36^. Herein we demonstrate that PPARδ depletion dramatically blocks cholesterol biosynthesis, leading to reduced levels of plasma membrane cholesterol. Cholesterol can trigger activation of Hedgehog signaling pathway, which initiates self-renewal of liver CSCs. Aberrant Hedgehog signaling causes several malignancies, such as basal cell carcinoma and pediatric medulloblastoma^62^. Cholesterol can bind to SMO and is critical for activation of SMO and subsequently Hedgehog signaling^25^. Cholesterol can interact with the extracellular SMO N-terminal cysteine-rich domain (CRD)^42,63,64^. However, we show that CRD-deleted SMO is also dependent on cholesterol for Hedgehog signaling activation in liver CSCs. A recent structural study reported that cholesterol binds a deep seven-transmembrane (7TM) domain of SMO^43^. We show that the 7TM site mutations of SMO (V333F or T470Q) cause a significant reduction of cholesterol-induced Hedgehog signaling activation, leading to impairment of liver CSC self-renewal. These data suggest PPARδ enhances liver CSC self-renewal and liver cancer development through regulation of cholesterol metabolism. Tetramethyl thiuram disulfide (thiram) is one of the important antimicrobial drugs, which is used in agriculture. Here we found that low doses (1.2 mg/kg/day) of thiram could reduce orthotopic implantation with patient-derived tumor cells (PDC) into NSG mice. Previous studies showed that thiram (30 μg/day) administration caused significant inhibition of glioma tumor development and remarkable reduction in metastatic growth of Lewis lung carcinoma, suggesting thiram as a potential inhibitor of angiogenesis raises the possibility for its therapeutical use in pathologies^65^. However, high doses of thiram can cause liver damage in chickens (at doses more than 100 mg/kg)^66^. Thiram could induce chronic liver failure in dogs in middle (1.4 mg (30 ppm)/kg/day) and high-dose (13.8 mg (300 ppm)/kg/day) for 104 weeks by blood biochemistry and/or histopathology, whereas the rats of the high-dose group have retarded growth with a slightly decreased food intake^67^. It still needs further pre-clinical investigation for therapeutical use of thiram to treat HCC patients.

We conclude that the TRMT6/TRMT61A complex may be a potential therapeutic target for HCC. Based on screening of 1600 known drugs, thimerosal, phenylmercuric acetate (PMA), and thiram are identified to be candidate inhibitors against the interaction of TRMT6 and TRMT61A. Thiram displays effective suppression tumor growth in vivo, suggesting that thiram exerts its inhibitory antitumour effect through blockade of enzymatic activity of the TRMT6/TRMT61A complex. Importantly, combination of thiram with GSK3787 has a synergetic effect on inhibition of liver cancer development and tumor growth with high m^1^A methylations. Our data indicate that m^1^A inhibitors combined with PPARδ antagonists may be an effective therapy for liver cancer. In summary, our work provides insights into the dynamic and regulatory role of m1A modification in tRNA underlying hepato-oncogenesis and the potential of TRMT6/TRMT61A as a drug target, which will pave a way to develop more effective therapeutic strategies for HCC patients.

## Methods

### Antibodies and reagents

The following commercial antibodies were used: Anti-1-methyladenosine (m^1^A) (clone no. AMA-2) and mouse IgG2a (isotype control, clone no. 6H3) antibodies were purchased from MBL (Japan). Anti-m^1^A antibody is specific for m^1^A and was prepared by Dr. Kunihiko Itoh^68^. It was used in immunohistochemistry and immunofluorescence for esophagus cancer detection and used in dot blots and m1A-seq assays by several groups^8,9,12^, and so on. The specificity of m1A antibody was tested by Liu et al.^12^. We also used isotype IgG2 for controls in our work for validation.

Anti-6-methyladenosine antibody (202003) was from Synaptic Systems (Germany). Phycoerythrin (PE)-anti-human CD133 (clone no. REA820, 130-112-195) was purchased from Miltenyi Biotec (Germany). PE-anti-human IgG (12-4998-82), fluorescein isothiocyanate (FITC)-anti-human CD13 (clone no. WM-15, 11-0138-42), FITC-anti-human IgG (31529) were purchased from eBioscience (San Diego, USA). Anti-TRMT6 (PA5-61409) for human IHC and immunoblotting detection, anti-PPARδ (PA1-823A), anti-SREBF2 (PA1-338) antibodies were from Thermo Fisher Scientific (Waltham, USA). Anti-TRMT6 (16727-1-AP) for mouse IHC and immunoblotting detection, anti-FABP1 (13626-1-AP), anti-APOA2 (16845-1-AP), anti-PPARα (15540-1-AP), anti-PPARγ (16643-1-AP) antibodies were from Proteintech (USA). Anti-ZNF780A (NBP1-79357), anti-ZNF821 (NBP2-82055), anti-HMGCR (NBP2-66888) were from Novus Biologicals (USA). Anti-HMGCS2 antibody (#20940S) was from Cell Signaling Technology, Inc. (USA). Anti-TRMT61A (SAB2700607) and anti-β-actin antibodies were from Sigma-Aldrich (USA). HRP-conjugated secondary antibodies were from ZSGB-BIO (Beijing, China). Secondary antibodies conjugated with Alexa-594, Alexa-488, or Alexa-649 were purchased from Molecular probes Inc (Eugene, USA).

SuperReal premix plus qPCR buffer was from TIANGEN Biotech (Beijing, China). Human HCC tissue microarray (TMA) chips were from Shanghai Outdo Biotech Co., LTD (Shanghai, China). Diethylnitrosamine (DEN,N0756), cholesterol (PHR1533), cholestanol (700064P), atorvastatin (PZ0001), methyl-β-cyclodextrin (M7439), GW501516 (SML1491), GSK3787 (516567), Thimerosal (PHR1587), Thiram (43966),Merbromin (M7011), Bronidiol (32053), Benserazide HCl (BP685), Disulfiram (PHR1690), Phenylmercuric acetate (P27127), Gallic acid (G7384) were purchased from Sigma-Aldrich (St. Louis, USA). Collagenase IV was from Invitrogen (USA). Total cholesterol assay kit (STA-390) was from Cell Biolabs (USA). Cholesterol cell-based detection assay kit (10009779) was from Cayman (USA).

### Cell lines, shRNA-mediated interference, overexpression and oncosphere formation assay

Human hepatocellular carcinoma (HCC) cell lines Hep3B, Huh7, and PLC/PRF/5 were obtained from ATCC and maintained in DMEM (GIBCO) supplemented with 10% fetal bovine serum (FBS), 100 μg/ml penicillin G, and 100 U/ml streptomycin (Invitrogen, NY, USA). Human 293T cells (ATCC, CRL-3216) were cultured with DMEM supplemented with 10% FBS and 100 U/ml penicillin and 100 mg/ml streptomycin. Oncosphere cells were seeded on ultra-low attachment culture dishes (Corning) in serum-free medium. DMEM/F12 serum-free medium (Invitrogen) contained 2 mM l-glutamine, 1% sodium pyruvate (Invitrogen), 100 μg/ml penicillin G, and 100 U/ml streptomycin supplemented with 20 ng/ml epithelial growth factor (Invitrogen), 10 ng/ml fibroblast growth factor-2 (Invitrogen), N2 (Invitrogen), and B27 (Invitrogen). Mycoplasma contamination was excluded using PCR detection.

For shRNA-mediated interference, shRNAs against TRMT6 and TRMT61A were designed with MIT’s siRNA designer (http://sirna.wi.mit.edu/home.php). At least eight shRNAs were designed for each gene, and to prevent off-target effects, the most two effective shRNAs were used for subsequent studies. The sequences of the effective shRNAs were provided (Supplementary Table 3). shRNAs against TRMT6, TRMT61A, and control hairpins were cloned into pSICO R vector. Production of lentiviral particles and transduction of CSCs was performed according to protocols from the RNAi consortium (http://www.broadinstitute.org/rnai/trc). Cells were transfected with lentiviral constructs expressing shRNA or shCtrl as described above for 24 h. Positive cells were selected with puromycin for 7 days. Then, cells were collected for protein and RNA analysis.

For CRISPR/Cas9 mediated gene editing, sgRNA sequences were cloned into lentiCRISPRv2-puro plasmid, sgRNA sequences were listed in Supplementary Table 3. LentiCRISPRv2, pVSVg (8454), and psPAX2 (12260) were transfected into 293T cells to generate CRISPR/Cas9 lentivirus.

For overexpression experiments, full-length of *TRMT6*, *TRMT61A*,mut-*TRMT6*, mut-*TRMT61A*, *PPARD*, mut-*PPARD*, *SREBF2*, WT-*Smo*, Mut-*Smo* were constructed into pSIN plasmid. Lentivirus was produced in 293T cells using the standard protocols. Transfection was performed using lipofectamine 3000 Reagent (Invitrogen). Then, cells were collected for PCR, DNA sequencing and immunoblotting analysis. At least three independent experiments were performed as biological replicates.

### Flow cytometry and cell sorting

Cells were stained using different antibodies according to the manufacturer’s instructions. Labeled cells were detected using a FACS Calibur (BD Immunocytometry Systems, San Jose, CA). For cell sorting, cocktail PE-conjugated anti-human CD133 and FITC-conjugated anti-human CD13 antibodies were incubated with HCC primary cells or HCC cell lines, followed by sorting with FACS Aria III (BD Immunocytometry Systems, San Jose, CA, USA). FACS data were analyzed by FlowJo software (BD, San Jose, CA).

### Sample selection and preparation

The paired HCC samples used in this study were obtained from the PLA General Hospital in China, with the approval of the Research Ethics Committee at the hospital. There are no biases in the selection of patients. All samples are renamed with codes (such as LC (tumor) #1, #2, LN (peri-tumor) #1, #2, and so on) instead of the patient’s name. Written informed consent was provided by all patients. Surgically resected primary tumor tissues and paired peri-tumor liver tissues were selected from 191 HCC patients who had not undergone prior chemotherapy or radiotherapy. Of these cases, 187 had an HBV-infection background. 138 patients were MVI−, and the diameters of the tumors were less than 5 cm. Clinical information—including gender, age, etiology, status of liver cirrhosis, clinical grade, tumor number, size, status of microscopic vascular invasion, serum AFP (Supplementary Table 1). After surgical resection, the tissue samples were digestion with collagenase IV followed by CSC FACS sorting. The tissue samples for protein analysis were frozen in liquid nitrogen for storage before use. The tissue samples for RNA analysis were placed in TRIzol (Invitrogen) and stored at −80 °C. All tissue samples were obtained from consenting patients and approved by the Institutional Review Board of the Institute of Biophysics, Chinese Academy of Sciences.

### Mice

*Trmt6*^flox/flox^ and *Trmt61a*^flox/flox^ mice on a C57BL6/J background were generated using CRISPR/Cas9 approaches^69^. To generate hepatocyte-specific *Trmt6*, *Trmt61a*, or *Trmt6*/*Trmt61a *double knockouts, *Trmt6*^flox/flox^, *Trmt61a*^flox/flox^, or *Trmt6*^flox/flox^/*Trmt61a*^flox/flox ^mice were crossed to albumin-Cre (Alb-Cre) transgenic mice (B6.Cg^*Tg(Alb-cre)21Mgn/*^J, Jackson Laboratory). H11-LSL-*Myc*transgenic mice(C57BL/6J*-Igs2*^*em1(CAG-LSL-Myc)Smo*^*)* were from Shanghai Model Organisms Center, Inc (China). To generate spontaneous liver cancer mouse models, H11-LSL-*Myc* mice were crossed with Alb*-*Cre transgenic mice and then generated aberrantly overexpressed of *myc* in liver cancer (*Myc*^OE^). Animals were killed when sick or when they developed tumors larger than 15 mm in their greater diameter or ulcerated lesions. We used littermates with the same age (8–12 weeks old) and gender for each group. We excluded the mice 5 g thinner than other littermates before any treatment or analysis. C57BL/6 mice and BALB/c mice were purchased from Charles River (China). Female NOD/SCID/g-chain knockout (NSG) mice were purchased from Biocytogen (China).

For adeno-associated viral vector (AAV) (Addgene #60231) infections, age-matched (8–10 weeks old) male mice were injected with 2.0 × 10^9^ p.f.u. virus encoded GFP or Pparδ by tail-vein injection unless otherwise specified. Mice were euthanized 8 days later after a 6-h fast. At the time of euthanization, liver tissue and blood was collected by cardiac puncture and immediately frozen in liquid nitrogen and stored at −80 °C. Liver tissue was processed for isolation of RNA, protein or fixation for histological staining as above. All mice were maintained in pathogen-free conditions at ambient temperature 20–22 °C, humidity 50–60% under standard 12 h light–dark cycle, fed stand rodent chow and water, and were used for experiments at age of 8–10 weeks. Mouse experiments were approved by the Institutional Animal Care and Use Committees at the Institute of Biophysics, Chinese Academy of Sciences.

### Diethylnitrosamine (DEN) induced hepatocarcinogenesis

Male mice were injected intraperitoneally with 25 mg kg^−1^ of DEN at 15 days of age. The mice were observed for development of tumors and survival for 1 year.

### Gene knockout by CRISPR/Cas9-mediated genome editing in vivo

*Ppard* gene deletion mice were established by CRISPR/Cas9-mediated genome editing in vivo as described^70^. Briefly, we cloned sgRNA into adeno-associated virus (AAV) vector (Addgene #60231), and transfected into 293T cells along with pHelper vector (Biovector NTCC Inc) and pAnc80L65 vector (Addgene #68837) for 72 h. Then transfected cells were lysed by repeated unfreezing and AAV was purified for splenic injection into CRISPR/Cas9 knockin mice (Jackson Laboratory, Stock no: 024857), and gene deletion efficiency was examined by Western blot 1 week of post-injection. sgRNA sequences used for this study were listed in Supplementary Table 3.

### Genotyping

Genotyping of the recombined *Trmt6* and *Trmt61a* allele was confirmed with the following primer sequences: *Trmt6*^flox/flox^: up F (5′- AGTCAGGGTTTTCCTGCC-3′), up R (5′-CTATGAAAAGTGTGCTAAT-3′), down F (5′-ATGGTGCTGGCAGGCTTC-3′), down R (5′-CAATCTTCTTGTCCCGAGT-3′); *Trmt61a*^flox/flox^: up F (5′-GAAGAACACAGCCGAAGAGAC-3′), up R (5′-GCACTTCCGGTGCTTTTTGA-3′), down F (5′-TGGTCTGTGAATCAGGTGAG-3′), down R (5′-GTTCAGCGCACAACTGCAGGA-3′); and the following cycling conditions: 1 cycle at 95 °C for 5 min; 35 cycles at 95 °C for 30 s, 55 °C for 30 s, 72 °C for 30 s; and 1 cycle at 72 °C for 8 min; hold at 4 °C. Genotyping of the Alb-cre allele was confirmed with the following primer sequences: Alb-cre-WT (TGCAAACATCACATGCACAC), Alb-cre-common (TTGGCCCCTTACCATAACTG), Alb-mut (GAAGCAGAAGCTTAGGAAGATGG), and the following cycling conditions: 1 cycle at 95 °C for 5 min; 30 cycles at 95 °C for 30 s, 58 °C for 30 s, 72 °C for 45 s; and 1 cycle at 72 °C for 10 min; hold at 4 °C. All mouse genotypes were verified by a PCR-based method and DNA sequencing.

### RNA extraction and RNA-seq

The TRIzol method was used for the RNA extraction from the liver tissues. RNA degradation and contamination were monitored on 1% agarose gels. Different RNA fractions were isolated from total RNA using MEGA clear Kit (Thermo). Large RNA (≥200 nt) was eluted from the column and small RNA (<200 nt) was recovered from the flow-through fraction by ethanol precipitation. RNA purity was assessed using a Nano Photometer spectrophotometer (Thermo). RNA concentration was measured using a Qubit RNA Assay Kit and a Qubit 2.0 Fluorometer (Life Technologies). RNA integrity was assessed using an RNA Nano 6000 Assay Kit and a Bioanalyzer 2100 system (Agilent Technologies). For liver CSCs and CSCs sorting, cocktail PE-conjugated anti-human CD133 and FITC-conjugated anti-human CD13 antibodies were incubated with HCC primary cells, followed by sorting with FACS Aria III (BD Immunocytometry Systems, San Jose, CA, USA). Cells were collected for total RNA extraction with Trizol reagent.

Genome wide expression profiling assay of cells with sg*PPARD* as well as sgCtrl was carried out in custom designed RNA-seq analysis (BGISEQ-500 Platform). The sequencing data was filtered with SOAPnuke (v1.5.2) by (1) Removing reads containing sequencing adapter; (2) Removing reads whose low-quality base ratio (base quality less than or equal to 5) is more than 20%; (3) Removing reads whose unknown base (“N” base) ratio is more than 5%, afterwards clean reads were obtained and stored in FASTQ format. The clean reads were mapped to the reference genome using HISAT2 (v2.0.4). Bowtie2 (v2.2.5) was applied to align the clean reads to the reference coding gene set, then expression level of gene was calculated by RSEM (v1.2.12). The heatmap was drawn by pheatmap (v1.0.8) according to the gene expression in different samples. Essentially, differential expression analysis was performed using the DESeq2 (v1.4.5) with *Q* value ≤0.05. To take insight to the change of phenotype, GO (http://www.geneontology.org/) and KEGG (https://www.kegg.jp/) enrichment analysis of annotated different expressed gene was performed by Phyper (https://en.wikipedia.org/wiki/Hypergeometric_distribution) based on Hypergeometric test. The significant levels of terms and pathways were corrected by *Q* value with a rigorous threshold (*Q* value ≤ 0.05) by Bonferroni.

### Dot blot

Total RNA was extracted by TRIZOL (Invitrogen). Purified RNA was quantified and diluted in 10 mM Tris-EDTA buffer. Equivalent amounts of RNA was denatured at 65 °C for 5 min and loaded to positively charged nylon membrane (GE Amersham). The membrane was UV-crosslinked with 2400 J/cm^2^ twice and then probed with anti-m^1^A antibody, followed with HRP-conjugated secondary antibody staining and ECL detection.

### Quantification of m^1^A levels by LC-MS/MS

Hundred nanogram isolated RNA was digested into nucleosides by 0.5 U nuclease P1 (Sigma) in 20 μL buffer containing 10 mM ammonium acetate, pH 5.3 at 42 °C for 6 h, followed by the addition of 2.5 μL 0.5 M MES buffer, pH 6.5 and 0.5 μL rSAP (NEB). The mixture was incubated at 37 °C for another 6 h and diluted to 100 μL. Five microliter of the solution was injected into LC-MS/MS. The nucleosides were separated by ultra-performance liquid chromatography with a C18 column, and then detected by triple-quadrupole mass spectrometer (AB SCIEX QTRAP 5500) in the positive ion multiple reaction-monitoring (MRM) mode. The mass transitions of *m*/*z* 282.0 to 150.1 (m^1^A), *m*/*z* 282.0 to 150.1 (m^6^A), *m*/*z* 268.0 to 136.0 (A) were monitored and recorded. Concentrations of nucleosides in RNA samples were deduced by fitting the signal intensities into the stand curves.

### Quantitative tRNA m^1^A-seq

Two hundred nanogram small RNA fraction (<200 nt; mainly composed of mature tRNA) was purified from total RNA using MEGA clear Kit (Thermo). Purified small RNA was deacylated by incubating in 0.1 M Tris-HCl, pH 9 at 37 °C for 45 min. Half of the deacylated small RNA fraction was subjected to demethylation treatment by using AlkB in vivo reaction. The total 20 µL mixture for AlkB-mediated demethylation includes 0.8 pmol purified AlkB protein, 50 mM MES (pH 6.5), 300 μM 2-ketoglutarate, 283 μM (NH_4_)_2_Fe(SO_4_)_2_·6H_2_O, 2 mM l-ascorbic acid and 0.4 U/μL RNase inhibitor. After 2 h incubation in 37 °C, 5 mM EDTA was added to quench the demethylation reaction, and the demethylated RNA was purified using phenol chloroform extraction and subsequent ethanol precipitation.

The demethylated and untreated RNA was together sent for library construction as previously described^9^. Specifically, the 3′ ends of RNA samples were first dephosphorylated using PNK under an 37 °C incubation for 1 h, and then heat-inactivated under 65 °C for 20 min, followed by ethanol precipitation. A 3′ RNA linker (5′rAPP-AGATCGGAAGAGCGTCGTG-3SpC3) was ligated to the dephosphorylated RNA using T4 RNA ligase2, truncated KQ (NEB) under 25 °C for 2 h. The excess RNA linker was digested by adding 1 µL 5′ Deadenylase (NEB) and 30 °C incubation for 1 h, and subsequently adding 1 μL RecJf (NEB) and at 37 °C incubation for 1 h. The reaction was then heat-inactivated at 70 °C for 20 min, the 3′ end ligated RNA was purified by ethanol precipitation. Ten microliter H_2_O was used to dissolve the recovered RNA pellet and 1 µL RT primer (ACACGACGCTCTTCCGATCT; 2 µM) was added. The RNA-primer mixture was denatured by a 2 min 80 °C incubation and then immediately chilled on ice. RT reaction buffer was prepared according to the following formula: a) 50 mM Tri–HCl (pH 8.3), b) 75 mM KCl, c) 3 mM MgCl_2_, d) 1 mM dNTPs, e) 5 mM DTT, f) 1 U/μL RNase Inhibitor and g) 1 μL TGIRT (InGex).The reaction buffer was well-mixed and then added to the denatured RNA-primer mixture, and incubated at 57 °C for 2 h for RT reaction. One microliter Exonuclease I (NEB) was added and incubated at 37 °C for 30 min to digest the excess RT primer. The resulted cDNA was purified with silane beads (Invitrogen), and then subjected to 5′ adapter ligation (5Phos- NNNNNNNNNNAGATCGGAAGAGCACACGTCTG-3SpC3) by a 25 °C overnight reaction after adding 1 μL high concentration T4 RNA ligase 1 (NEB). The resulted cDNA was again purified with silane beads. PCR amplification was carried out using the following primers: 5′-AATGATACGGCGACCACCGAGATCTACACTCTTTCCCTACACGACGCTCTTCCGATCT-3′, 5′-CAAGCAGAAGACGGCATACGAGATXXXXXXGTGACTGGAGTTCAGACGTGTGCTCTTCCGATC-3′ (XXXXXX represents index sequence). The library was purified using 8% TBE gel, and then subjected to sequencing on Illumina Hiseq X10 platform (PE 150).

### tRNA m^1^A-seq data analysis

The preprocessing of tRNA m^1^A-seq sequencing data was the same as previously described^9^. Briefly, Read 2 of sequencing data was first subjected to quality control and adapter removal using Trim_galore (http://www.bioinformatics.babraham.ac.uk/projects/trim_galore/), with a minimum quality of 20. The first 10 nt random barcode at the 5′ end was further removed. Processed reads were then mapped to tRNA sequences, which was downloaded from UCSC Table Browser, using BWA-MEM (version 0.7.17-r1188) with default parameters. The reads that aligned to an identical position and meanwhile with the same 10 nt random barcodes were regarded as PCR duplications, and only one of these reads was kept for further analysis. The mismatch information for each position was extracted from the de-duplicated alignment files. The m^1^A modification at position 58 for each tRNA was first identified by comparing the mismatch rates with and without demethylation treatment. The m^1^A58 modification stoichiometry for each tRNA was represented by the misincorporation rate of the corresponding position. The methylation level change for tRNA m^1^A58 between CSC and non-CSC cell lines was evaluated.

### Ribo-seq data analysis

The reads from Ribo-seq were first subjected to adapter trimming and quality control (Q20) using trim_galore. The trimmed reads with a length between 25–33 nt were considered qualified ribosome footprint and kept for further analysis. The processed reads were mapped to human transcriptome (Refseq annotation, downloaded from UCSC Table Browser) using BWA-MEM (version 0.7.17-r1188) with default parameters. To confirm the quality of the Ribo-seq data, the 12 nt offset analysis between 5′ end of the ribosome footprint and the P-site codons at translation initiation site was carried out^30^. RPKM (Reads Per Kilobase per Million mapped reads) of the CDS of each transcript was calculated. Translation efficiency (TE) was defined as the ratio of the RPKM in Ribo-seq to the corresponding RPKM in RNA-seq. The transcripts with a fold change of 4 in TE were defined as differentially translated genes.

### Dual-luciferase reporter system

To validate the tRNA m^1^A58 mediated translational alteration, a dual-luciferase reporter assay was employed. The dual-luciferase construct was based on pmirGlo vector (Promega), which subsequently includes a firefly luciferase (F-luc) and a Renilla luciferase (R-luc). The R-luc provided a control to adjust for the transfection levels and hence allowed a fair comparison to evaluate the effect of tRNA m^1^A on codon content. We constructed two plasmids with six-repeated GCU and GAG inserted before the F-luc, respectively.

We first performed overexpression for TRMT6 and TRMT61A (250 ng each) in non-CSC cells in a 6-well plate. To further illustrate the tRNA m^1^A58 dependent translational alteration, we also performed overexpression for catalytic mutant of TRMT6 (R377L) and TRMT61A (D181A). After 24 h overexpression of TRMT6/61A, we performed the transfection of the constructed dual-luciferase plasmids (500 ng). After another 24 h, the cells were resuspended with 100 µL PBS, with 10 μL of each transferred to a 96-well plate. The luciferase intensity was measured using Dual-Glo Luciferase Assay System (Promega).

### Proteomics analysis

Protein extraction and digestion: The cell samples were disrupted by the ultrasonic processor on ice in lysis buffer (8 M urea/0.1 M Tris-HCl, pH 8.0) containing 1× Protease Inhibitor Cocktail (Roche). After centrifugation, the extracted proteins were reduced with 10 mM DTT for 2 h at room temperature followed by alkylation with 20 mM iodoacetamide for 30 min in the dark. The protein solution was diluted 1:5 with 50 mM triethylammonium bicarbonate (TEAB) and digested with trypsin (1:50) at 37 °C overnight. The digestion was desalted on OASIS HLB column and peptides eluted with 60% acetonitrile were lyophilized via vacuum centrifugation.

Peptides labeled with TMT: The dried peptides were dissolved with 100 mM TEAB buffer prior to label with Tandem Mass Tags (TMT). 100 µg of protein from each biological replicate of different experimental conditions was labeled with TMT six-plex® (Thermo Scientific) according to the manufacturer’s instructions. The cell samples were labeled as follows: TMT-126/-127/-128 was used for PLC/PRF/5 shCtrl CSCs and TMT-129/-130/-131 for PLC/PRF/5 shTRMT6/shTRMT61A CSCs. High pH reversed phase HPLC fractionation: Before nanoLC-MS/MS analysis, samples were fractionated using a Waters XBridge BEH130 C18 5 μm 4.6 × 250 mm column on an L-3000 HPLC System (Rigol) operating at 0.7 mL/min. All fractions were collected at 90 s intervals and concatenated into 12 post-fractions.

LC-MS/MS analysis: All nanoLC-MS/MS experiments were performed on a Q Exactive (Thermo Scientific) equipped with an Easy n-LC 1000 HPLC system (Thermo Scientific). The labeled peptides were loaded onto a 100 μm id × 2 cm fused silica trap column packed in-house with reversed phase silica (Reprosil-Pur C18 AQ, 5 μm, Dr. Maisch GmbH) and then separated on an a 75 μm id × 20 cm C18 column packed with reversed phase silica (Reprosil-Pur C18 AQ, 3 μm, Dr. Maisch GmbH). The peptides bounded on the column were eluted with a 78-min linear gradient. The solvent A consisted of 0.1% formic acid in water solution and the solvent B consisted of 0.1% formic acid in acetonitrile solution. The segmented gradient was 5–8% B, 8 min; 8–22% B, 50 min; 22–32% B, 12 min; 32-95% B, 1 min; 95% B, 7 min at a flow rate of 310 nL/min.

The MS analysis was performed with Q Exactive mass spectrometer (Thermo Scientific). With the data-dependent acquisition mode, the MS data were acquired at a high resolution 70,000 (*m*/*z* 200) across the mass range of 300–1600 *m*/*z*. The target value was 3.00E+06 with a maximum injection time of 60 ms. The top 20 precursor ions were selected from each MS full scan with isolation width of 2 *m*/*z* for fragmentation in the HCD collision cell with normalized collision energy of 32%. Subsequently, MS/MS spectra were acquired at resolution 17,500 at *m*/*z* 200. The target value was 5.00E+04 with a maximum injection time of 80 ms. The dynamic exclusion time was 40 s. For nano electrospray ion source setting, the spray voltage was 2.0 kV; no sheath gas flow; the heated capillary temperature was 320 °C.

Protein Identification and quantification analysis: The raw data from Q Exactive were analyzed with Proteome Discovery version 2.2.0.388 using Sequest HT search engine for protein identification and Percolator for FDR (false discovery rate) analysis. The Uniprot human protein database (updated on 10-2017) was used for searching the data from cell samples. Some important searching parameters were set as following: trypsin was selected as enzyme and two missed cleavages were allowed for searching; the mass tolerance of precursor was set as 10 ppm and the product ions tolerance was 0.02 Da.; TMT 6plex (lysine and N-terminus of peptides) and cysteine carbamidomethylation were specified as fixed modifications; The methionine oxidation was chosen as variable modifications. FDR analysis was performed with Percolator and FDR <1% was set for protein identification. The peptides confidence was set as high for peptides filter. Proteins quantification was also performed on Proteome Discovery 2.2.0.388 using the ratio of the intensity of reporter ions from the MS/MS spectra. Only unique and razor peptides of proteins were selected for protein relative quantification. The co-isolation threshold was specified as 50% and average reporters *S*/*N* value should be above 10. The normalization mode was selected as total peptide amount to corrected experimental bias.

### Patient-derived xenograft (PDX) liver cancer model and treatment

Patient-derived samples were obtained from patients who had given informed consent. Human hepatocytes were isolated by a collagenase digestion method. Briefly, the liver cancer tissues were sheared into 1 mm^3^ small pieces. Subsequently, the small pieces were further digested using collagenase IV (0.1%) and dispase (4 mg mL^−1^; Invitrogen) at 37 °C for 1 h. The liver was then dissociated in suspension buffer and filtered with 70 μm cell strainer. Hepatocytes were collected by centrifugation at 160 × *g* for  8 min. Passage-1 PDXs were then orthotopically transplanted into NSG mice via injection with 2 × 10^5^ hepatocytes. Drug treatments were started 10 days after transplantation. Thiram (1.6 mg kg^−1^) and/or GSK3787 (5 mg kg^−1^) were intraperitoneally injected on days every other day. Tumor growth was detected at day 7, 14, 21, 28, 35, 42 after treatment.

### IVIS in vivo imaging

Detection of luciferase activity was performed in an IVIS-100 imaging system. Five minutes before the procedure, mice were injected intraperitoneally with D-luciferin, bioluminescence substrate (Sigma) according to the manufacturer’s instructions. Living Image 4.3 software (Perkin Elmer) was used for analysis of the images after acquisition.

### Xenograft growth in nude mice

For subcutaneous injection models, different dilutions of control and treated cells were implanted into mice (male BALB/c nude mice), aged 6–8 weeks, with a matrigel scaffold (BD matrigel matrix, BD biosciences) into two sides of the same nude mouse at the posterior dorsal flank region (*n* = 6–12 per group). Tumors were measured every other day. All experimental procedures were approved by the Committee on the Use of Live Animals in Teaching and Research of the Institute of Biophysics, Chinese Academy of Sciences (CAS). The mice were maintained under standard conditions according to the institutional guidelines for animal care.

### Untargeted lipid metabolomics assay and analysis

Firstly, the sample is subjected to metabolite extraction by organic reagent precipitation protein method, and quality control (QC) samples are prepared at the same time (mixing the prepared experimental samples), and the extracted samples are tested on the machine. A QC sample is used to balance the instrument (monitoring the state of the instrument during liquid chromatography-mass spectrometry), and then a QC sample is interspersed per ten test samples in general (if the number of samples is small, the number of interspersed QC samples is appropriately increased), the last three QC samples ended the experiment. Here many QC designs can evaluate the quality of sample data acquisition. All samples were acquired by the LC-MS system followed machine orders. Firstly, all chromatographic separations were performed using an ultra-performance liquid chromatography (UPLC) system (Waters, UK). An ACQUITY UPLC CSH C18 column (100 mm × 2.1 mm, 1.7 μm, Waters, UK) was used for the separation. The column oven was maintained at 55 °C. The flow rate was 0.4 mL/min and the mobile phase consisted of solvent A (ACN: H2O = 60:40, 0.1% formate acidand 10 mM ammonium formate) and solvent B (IPA: ACN = 90:10, 0.1% formate acid and 10 mM ammonium formate). Gradient elution conditions were set as follows: 0–2 min, 40–43% phase B; 2–7 min, 50–54% phase B; 7.1–13 min, 70–99% phase B; 13.1–15 min, 40% phase B. The injection volume for each sample was 5 μL. A high-resolution tandem mass spectrometer Xevo G2 XS QTOF (Waters, UK) was used to detect metabolites eluted form the column. The Q-TOF was operated in both positive and negative ion modes. For Positive ion mode, the capillary and sampling cone voltages were set at 3.0 kV and 40.0 V, respectively. For Negative ion mode, the capillary and sampling cone voltages were set at 2 kV and 40 V, respectively. The mass spectrometry data were acquired in Centroid MSE mode. The TOF mass range was from 100 to 2000 Da in positive mode and 50 to 2000 Da in negative mode. And the survey scan time was 0.2 s. For the MS/MS detection, all precursors were fragmented using 19–45 eV, and the scan time was 0.2 s. During the acquisition, the LE signal was acquired every 3 s to calibrate the mass accuracy. Furthermore, in order to evaluate the stability of the LC-MS during the whole acquisition, a quality control sample (Pool of all samples) was acquired after every ten samples.

The raw data from mass spectrometer was imported into commercial software Progenesis QI (version 2.2, hereinafter referred to as QI) for peak picking. In results we get information of metabolites such as mass over charge, retention time and ion area. The QI workflow consists of the following steps: peak alignment, peak picking, and peak identification. Data preprocessing was performed using metaX, the steps include: Filtering out low quality ions (first removed ions in QC sample that contain over 50% missing value, then removed ions in actual samples that contain over 80% missing value); Using KNN method for filling the missing values; Using pqn method for data normalization; Using QC-RSC (Quality control-based robust LOESS signal correction) method for batch effect correction; Filling missing value again; Filtering out ions in all QC samples which are RSD > 30% (the ions with RSD > 30% are fluctuate greatly in the experiment and will not be included in downstream statistical analysis).

### Immunoblotting assay

Cells were lysed in RIPA lysis buffer supplemented with cocktail protease inhibitor (Roche). Five-to-twelve percent Bis–Tris protein gels were equally loaded with 30 μg proteins, electrophoresed at 120 V, and transferred onto polyvinylidene difluoride (PVDF) membrane (GE Healthcare). The PVDF membranes were incubated with primary antibodies, followed by incubation with secondary antibodies coupled to horseradish peroxidase (R&D systems). Signals were visualized with a chemiluminescence system.

### Immunohistochemistry and TMA staining

Tumor samples were fixed in 4% formaldehyde for 24 h at room temperature, moved into 70% ethanol for 12 h, and then embedded in paraffin. After cutting (Leica RM2235) and baking at 60 °C for 20 min for de-paraffinization, slides were treated for antigen unmasking. For immunohistochemical staining, endogenous peroxidases were inactivated by 3% hydrogen peroxide at room temperature (RT) for 15 min. Non-specific signals were blocked with 5% BSA and 5% goat serum for 1 h. Tissues were stained with primary antibodies for 12 h at 4 °C. After washing with PBS-T, tissues were stained with secondary antibodies against mouse, rabbit, or goat for 1 h, RT. For immunofluorescence, secondary antibodies conjugated to Alexa594 or Alexa488 (Molecular Probes) were used. Images were captured with Olympus confocal microscope.

Five TMA chips containing 90 HCC tumor and paired non-tumor liver tissues were purchased from Shanghai Outdo Biotech Co., LTD (China).The staining extent score ranged from 0 to 3 based on the percentage of immunoreactive tumor cells (Score 0, 0–5% positive cells/section; Score 1, 5–15%; Score 2, 15–35%; Score 3, >35%). The staining intensity was scored as negative (score = 0), weak positive (score = 1)or strong positive (score = 2 or 3).

### Quantitative real time PCR (qRT-PCR) assay

Total RNA was isolated using TRIzol (Invitrogen) and an RNeasy kit (QIAGEN) with DNase I digestion according to the manufacturers’ instructions. cDNA was synthesized from total RNA using M-MLV reverse transcriptase (Promega) and random primers (Promega). qRT-PCR was performed on an ABI7300 Real-Time PCR System(ABI 7300, Applied Biosystems). Data were normalized to 18 S rRNA or β-actin or to control samples. Primers sequences for the detected genes were listed in Supplementary Table 2.

### Cholesterol assay

Cellular total cholesterol levels were measured using a Total Cholesterol Assay Kit (Cell Biolabs). In brief, cellular lipids were extracted using chloroform: 2-propanol:NP-40 (7:11:0.1) in a micro-homogenizer, and the levels of total cholesterol and free cholesterol were determined according to the manufacturer’s instructions. The amount of cholesterol ester was calculated by subtracting the amount of free cholesterol from the amount of total cholesterol.

### Drug-target mapping

Drug targets with FDA-approved drugs including 1600 compounds were extracted from the Drug Bank database. Only targets with known pharmacological action and significant blocking effect on TRMT6/TRMT61A protein interaction were selected as candidates for m^1^A inhibitor.

### Statistical analysis

Data were analyzed with a double-sided Student’s *t*-test using the SPSS 13.0 software and GraphPad Prism 8. *P* value for the Kaplan–Meier curve was determined using a log-rank test. Tumourigenic cell frequency was calculated based on extreme limiting dilution analysis (ELDA) (http://bioinf.wehi.edu.au/software/elda/45). *P* values less than 0.05 were considered statistically significant.

### Reporting summary

Further information on research design is available in the Nature Research Reporting Summary linked to this article.

## Supplementary information

Supplementary Information

Reporting Summary

## Data Availability

All data supporting the findings of this study are available within the paper. Raw and processed data from the m^1^A RNA sequencing and ribosome profiling data of samples have been deposited to the NCBI Gene Expression Omnibus (GEO) under accession number GSE147840. Proteomics data have been deposited in the PRIDE database with accession number PXD019576. Untargeted lipid metabolomics assay can be obtained from the Metabo Lights database with the identifier MTBLS1781. Gene expression profiles by RNA-seq can be obtained from Gene Expression Omnibus with accession number GSE152108. Source data of gels and dot blots are provided with this paper. All relevant data are available from the corresponding author upon reasonable request. Source data are provided with this paper.

## Source data

Source Data
