# Supplementary material for: N1-methyladenosine methylation in tRNA drives liver tumourigenesis by regulating cholesterol metabolism
Source: Nat Commun. 2021 Nov 2;12:6314. doi: 10.1038/s41467-021-26718-6 (PMC8563902; doi:10.1038/s41467-021-26718-6)
Supplement: Supplementary file 1 — Supplementary Information [file 41467_2021_26718_MOESM1_ESM.pdf]

# Supplementary Information

## Supplementary Figure 1-7 and figure legends

## Supplementary Table 1-3

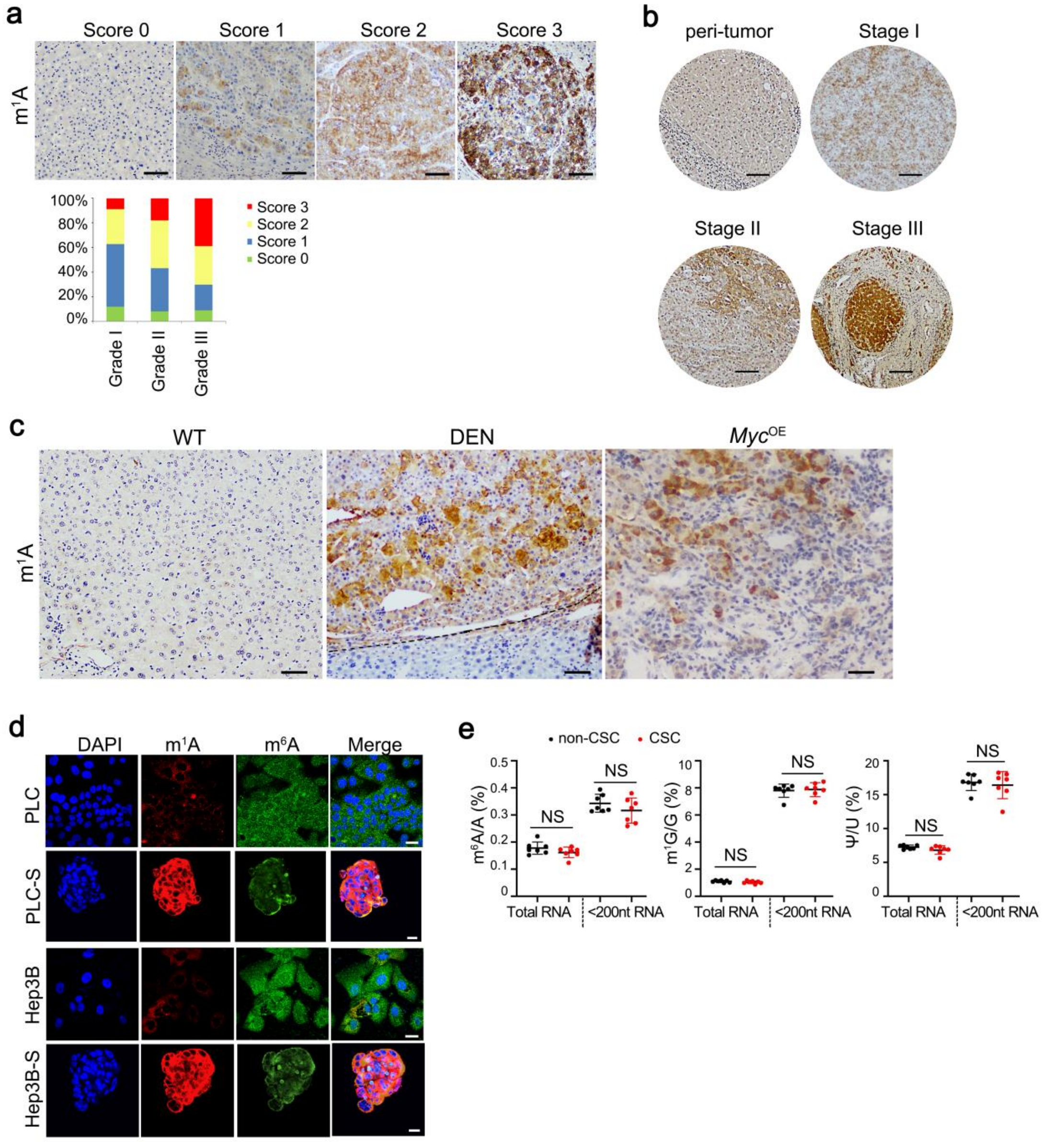


**Supplementary Figure 1. m$^1$A modifications in RNA is high in liver CSCs and HCC tissues.** (a) Representative immunohistochemistry images of m$^1$A levels (brown) in a panel of 191 clinically defined HCC samples. n=3 independent samples. Score 0 (0~5% positive cells/tissue section) represents m$^1$A staining that was considered negative, score 1 (5~15% positive cells/tissue section) represents m$^1$A staining that was considered week positive, whereas scores 2 (15~35% positive cells/tissue section) and 3 (>35% positive cells/tissue section) represent m$^1$A staining that was considered positive. Grade I (*n* = 56), Grade II (*n* = 87), Grade III (*n* = 48). Scale bar, 100 μm. (b) Representative immunohistochemistry images of m$^1$A signals in human HCC tissue microarray (TMA). Scale bar, 100 μm. n=2 independent samples. (c) Representative immunohistochemistry

images of $m^1A$ signals in two kinds of mouse liver cancer models including DEN-induced liver cancer and *Myc*$^{OE}$ liver cancer tissues. Scale bar, 100 μm. n=5 biologically independent mice. (d) Immunofluorescence staining of $m^1A$ and $m^6A$ signals in HCC cell lines and oncospheres. Scale bar, 10 μm. n=4 biologically independent samples. (e) LC-MS/MS quantification of $m^6A/A$, $m^1G/G$ and Ψ/U ratios in total RNAs and small RNA fraction (<200 nt) purified from liver cancer stem cells (CSCs). Data are means ± SD. n=7 biologically independent samples. Exact *P* values from left to right: 0.12, 0.15, 0.078, 0.11, 0.058, 0.062. **P* < 0.05; ***P* < 0.01; ****P* < 0.001, and NS, not significant (*P* > 0.05) by two-tailed Student's *t* test.

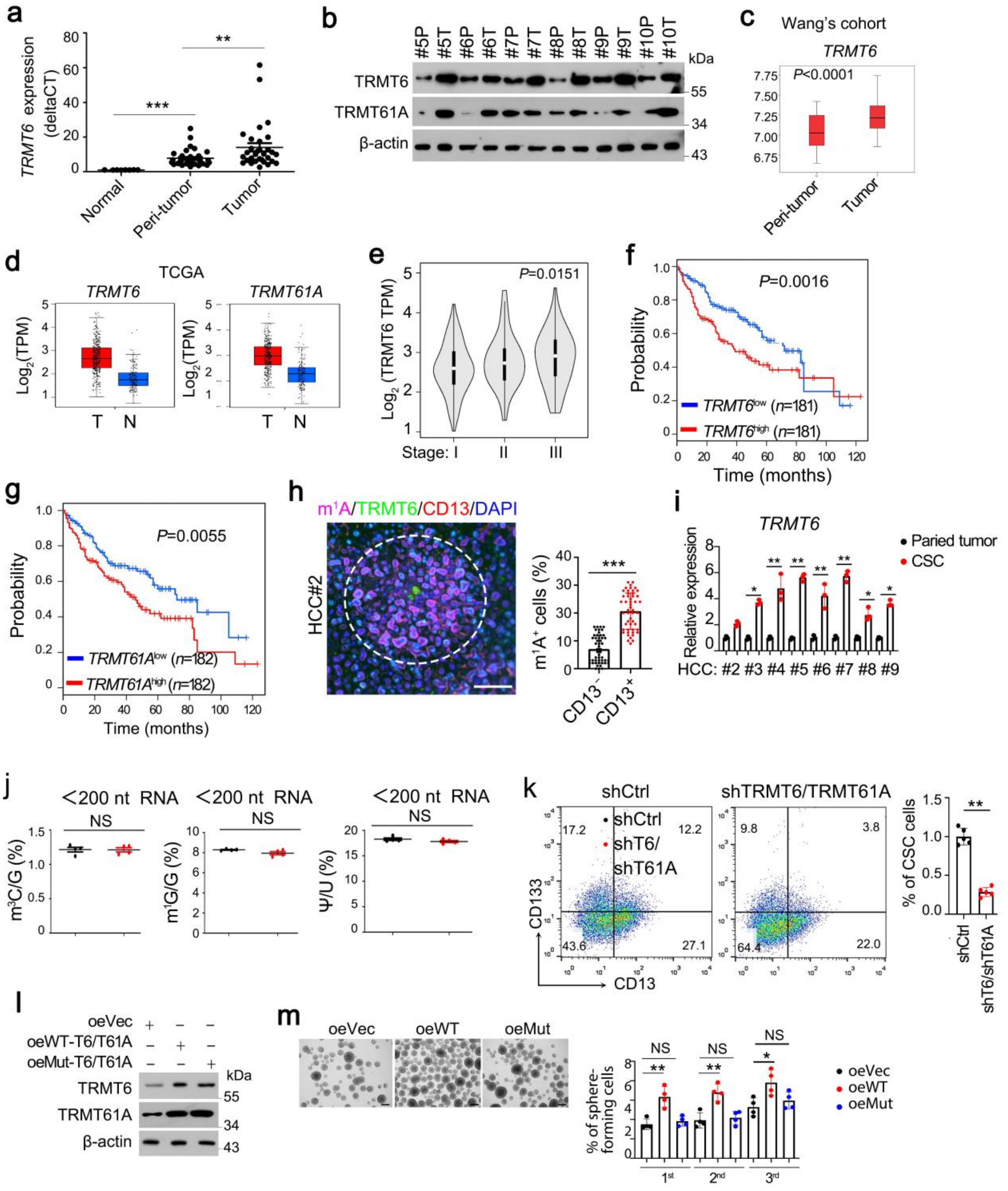


**Supplementary Figure 2. TRMT6/TRMT61A components are highly expressed in liver CSCs and HCC tissues.** (a) *TRMT6* mRNA was highly expressed in our liver cancer tissue cohort including tumors (*n*=30) and paired per-tumors (*n*=30) by quantitative PCR with reverse transcription (qRT-PCR) analyses. Data were normalized to endogenous 18S rRNA expression and 8 normal liver tissues were assigned with a value of 1. Data are means ± SD. Exact *P* values from left to right: 0.00013, 0.0055. (b) Western blot of TRMT6 protein levels in a panel of peri-tumor (P) and tumor (T) tissues. n=3 biologically independent samples. (c-e) Expression analyses of *TRMT6* and *TRMT61A* in Wang's and

TCGA cohorts. Expression values were $log_2$-transformed and mean centered. Data were shown as box plots. Centre, lower and upper lines correspond to median, first and third quartiles, respectively; whiskers extend to 1.5 × interquartile range. Exact *P* value for (c): 0.000025. (f, g) Kaplan–Meier plots of overall survival for *TRMT6* and *TRMT61A* in TCGA cohort. *P* values for Kaplan-Meier curve were determined using a two-sided log-rank test. (h) $m^1A$ signals were highly expressed in $CD13^+$ cells in liver tissues by immunofluorescence staining. Scale bar, 100 μm. Bar graph data are means ± SD. *n*=50 samples. Exact *P* value: 0.00039. (i) *TRMT6* was highly expressed in CSCs sorted from HCC tissues compared with paired tumor cells by qRT-PCR analyses. Data were normalized to endogenous 18S rRNA expression and paired tumor tissues were assigned with a value of 1. Data are means ± SD. n=8. Exact *P* values from left to right: 0.038, 0.0087, 0.0062, 0.0095, 0.0084, 0.023, 0.035. (j) LC-MS/MS quantification of $m^3C$, $m^1G$ and pseudouridine (Ψ) levels in tRNA (＜200 nt) purified from indicated liver CSCs, presented as percentage of unmodified G and U, respectively. Data are means ± SD. n=4. (k) $CD13^+CD133^+$ (CSC) subpopulations were detected in TRMT6/61A -depleted Huh7 cells by FACS analysis. Graph results are shown as means ± SD. Exact *P* value: 0.0056. n=5 biologically independent samples. (l, m) Effect of overexpression of wild type (WT)- or mutant (Mut)-TRMT6/TRMT61A cells on oncosphere formation. Scale bars, 100 μm. oe, overexpression. Exact significant *P* values from left to right: 0.0031, 0.0027, 0.019. n=4 biologically independent samples. **P* < 0.05; ***P* < 0.01, ****P* < 0.001 by two-tailed Student's *t* test.

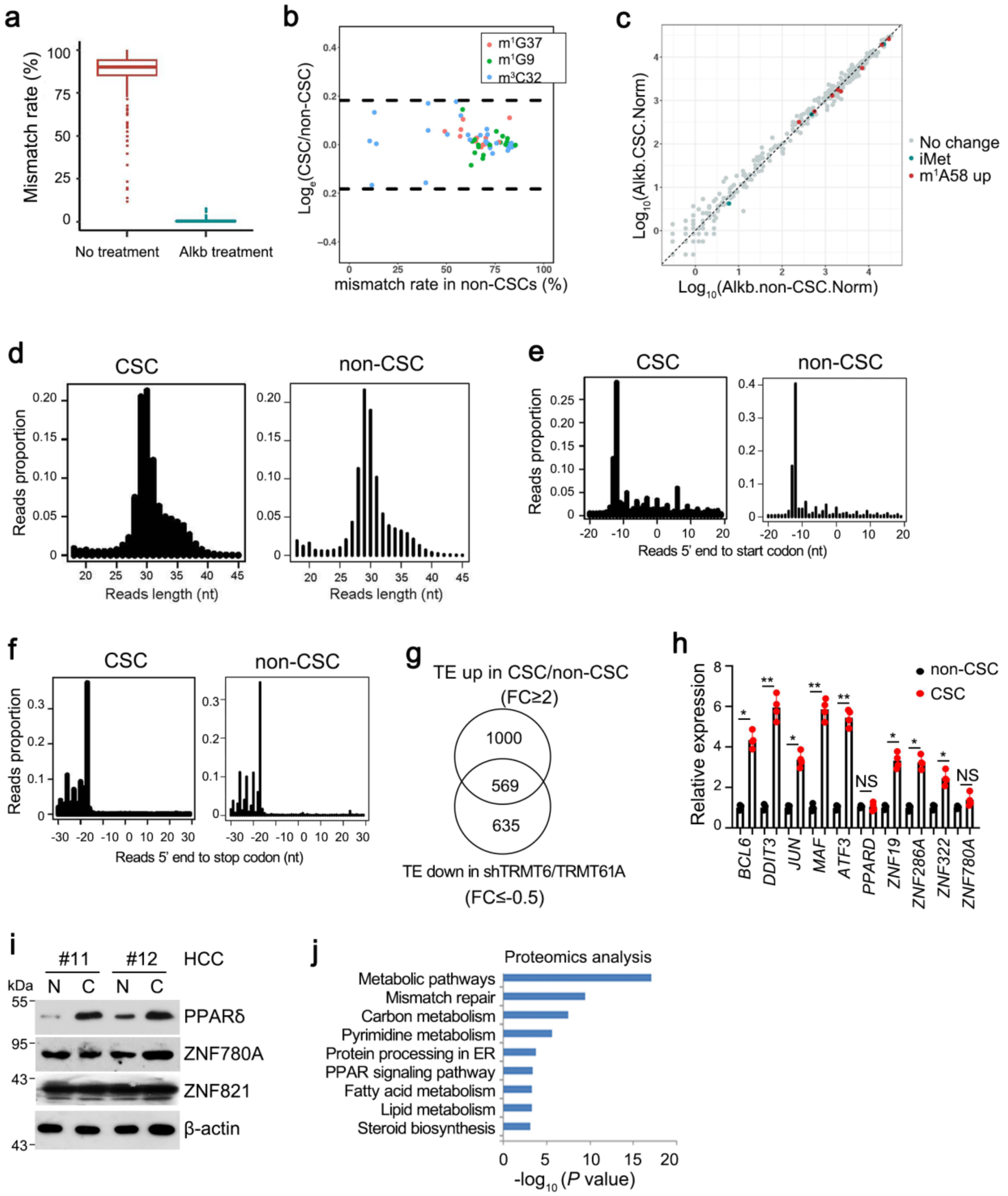


**Supplementary Figure 3. m$^1$A-seq and ribosome profiling sequencing (ribo-seq) analyses in liver CSCs.** (a) Misincorporation signals caused by m$^1$A58 in tRNAs dramatically decreased upon AlkB treatment. Data were shown as box plots. Centre, lower and upper lines correspond to median, first and third quartiles, respectively; whiskers extend to 1.5 × interquartile range. Points show outliers. (b) Other tRNA modifications including m$^1$G37, m$^1$G9, and m$^3$C32 between CSCs and non-CSCs. (c) The expression level of each tRNA between CSCs and non-CSCs. (d) Distance between 5' of ribosome footprint to the start codon in PLC/PRF/5 CSCs. (e) Distance between 5' of

ribosome footprint to the stop codon in PLC/PRF/5 CSCs. (f) Length distribution of mRNA-mapped reads in PLC/PRF/5 CSCs. (g) Differential genes of TE upregulated in CSCs and TE downregulated in TRMT6/TRMT61A depleted CSCs. (h) qRT-PCR analysis of 10 TFs in liver CSCs and non-CSCs from HCC patient samples. Data were normalized to endogenous 18S rRNA expression and non-CSCs were assigned with a value of 1. Data are means ± SD. n=4. *$P < 0.05$, ***$P < 0.001$, and NS, not significant ($P > 0.05$) by two-tailed Student's *t* test. Exact *P* values from left to right: 0.015, 0.0033, 0.032, 0.0051, 0.0069, 0.12, 0.011, 0.026, 0.038, 0.067, 0.031. (i) Immunoblotting analysis of candidate TFs in liver CSCs (C) and non-CSCs (N) from HCC samples. n=3 biologically independent samples. (j) Unbiased pathway analysis of proteins downregulated in TRMT6/TRMT61A depleted CSCs compared with control CSCs identified by proteomics analyses.

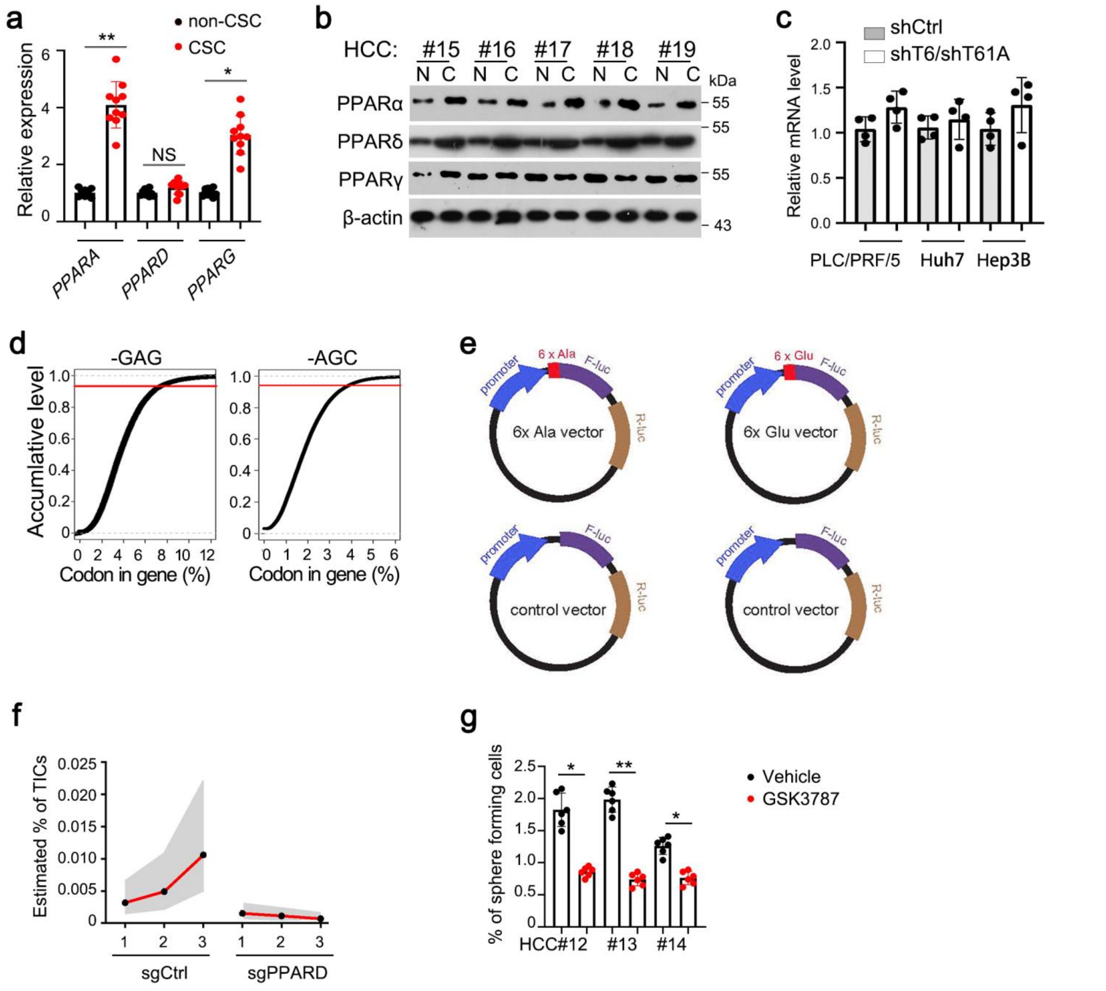


**Supplementary Figure 4. PPARδ promotes self-renewal of liver CSCs.** (a) *PPARA, PPARD* and *PPARG* mRNA expression in liver CSCs and non-CSCs. Data were normalized to endogenous 18S rRNA expression and non-CSCs were assigned with a value of 1. Data are means ± SD. n=10. Exact *P* values from left to right: 0.0067, 0.055, 0.011. (b) Western blotting confirmation of PPARα, PPARδ, PPARγ in liver CSCs. N: non-CSC. C: CSC. n=3 biologically independent samples. (c) *TRMT6/61A* depletion did not alter *PPARD* mRNA levels in liver CSCs. Data were normalized to endogenous 18S rRNA expression and shCtrls were assigned with a value of 1. Data are means ± SD. n=4. (d) Codon frequency analysis of *PPARD* mRNA among all genes. (e) Illustration of luciferase reporter plasmids. 6×GCU (Ala)-coding sequences (recognized by $tRNA^{Ala(AGC)}$) or 6×GAG (Glu)-coding sequences (recognized by $tRNA^{Glu(CTC)}$) were inserted before Firefly luciferase, respectively and Renilla luciferase was used as a transfection control. Control reporter without any insertion was used to normalize translation differences

among different cell lines. (f) Estimated frequency of TICs in PPARδ depleted and control HCC cells during serial transplantations. (g) Oncosphere formation capacity of HCC primary cells treated with PPARδ antagonist GSK3787 (20 μM) and DMSO (vehicle). Data are means ± SD. n=6. Exact *P* values from left to right: 0.032, 0.0066, 0.045. *$P < 0.05$; **$P < 0.01$ and NS, not significant ($P > 0.05$) by two-tailed Student's *t* test.

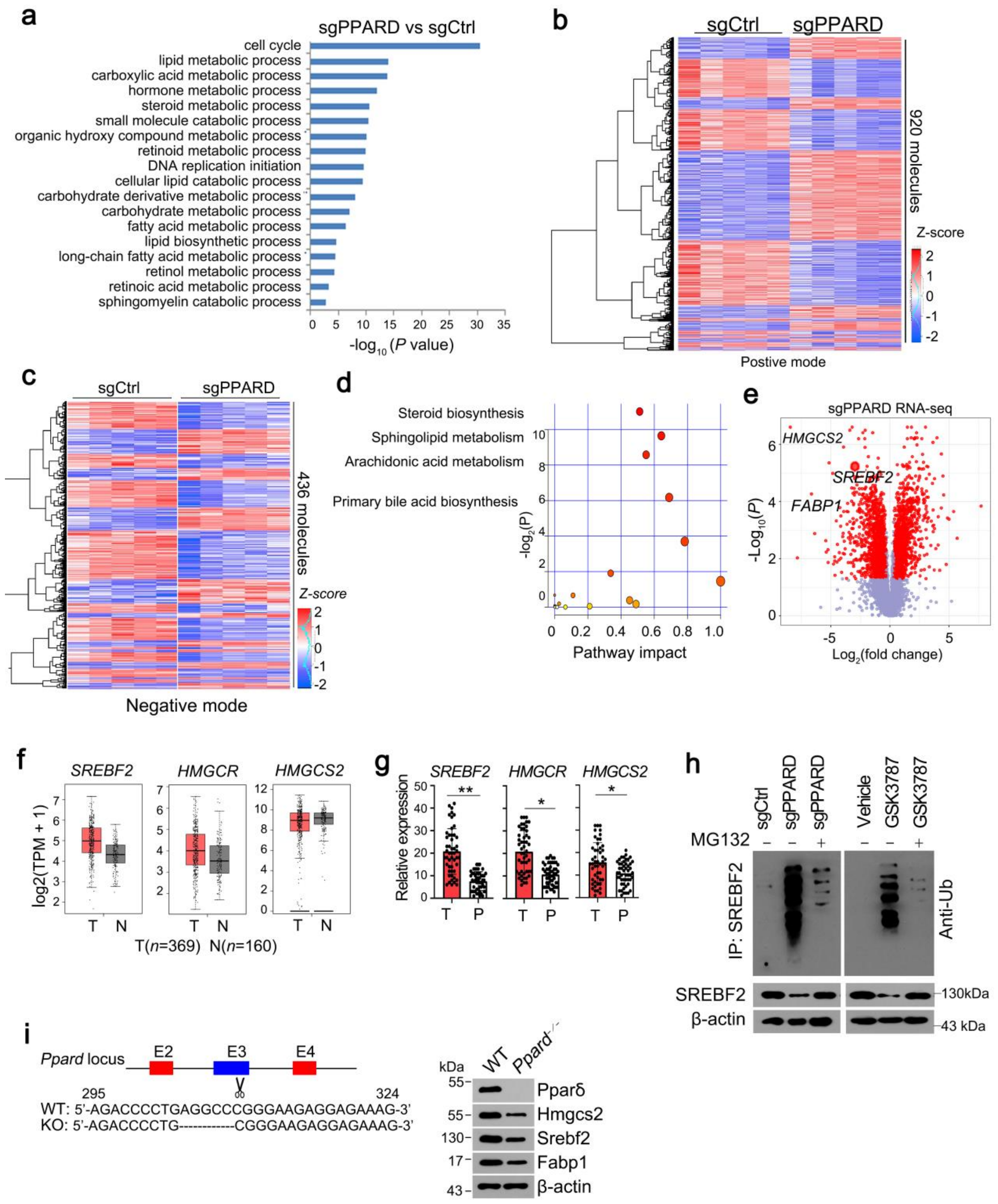


**Supplementary Figure 5. *PPARD* depletion reduces cholesterol synthesis in liver CSCs.** (a) Gene ontology (GO) analysis of genes downregulated in PPARδ depleted compared with control CSCs. (b) Heatmap analysis of lipidomics profiling under positive ion mode in PPARδ depletion compared with control CSCs. (c) Heatmap analysis of lipidomics profiling under negative ion mode in PPARδ depleted liver CSCs compared with control CSCs. (d) Pathway analysis of differential lipids regulated by PPARδ. (e) Volcano map analysis of PPARδ depleted compared with control CSCs. (f) Levels of cholesterol

synthesis related genes in HCC tumor and normal tissues from TCGA dataset. Data were shown as box plots. Centre, lower and upper lines correspond to median, first and third quartiles, respectively; whiskers extend to 1.5 × interquartile range. (g) qRT-PCR analysis of cholesterol synthesis related genes in our HCC dataset including tumor tissues paired with adjacent tumor tissues (*n*=50). Data were normalized to endogenous 18S rRNA expression and 7 normal liver tissues were assigned with a value of 1. Data are means ± SD. Exact *P* values from left to right: 0.0028, 0.019, 0.017. T, tumor. P, peri-tumor. (h) Western blots for SREBF2 ubiquitination. Samples are immunoprecipitated SREBF2 from PPARδ–silenced and GSK3787 treated CSCs and control CSCs. n=3 biologically independent samples. (i) Schematic diagram of *Ppard* KO mice using CRISPR/Cas9 knockin strategy. Frameshift mutations were identified by DNA sequencing. Right panel: Western blotting detection of Pparδ and cholesterol homeostasis-related proteins in *Ppard* knockout mouse livers. n=4 biologically independent mice.**P* < 0.05; ***P* < 0.01 by two-tailed Student's *t* test.

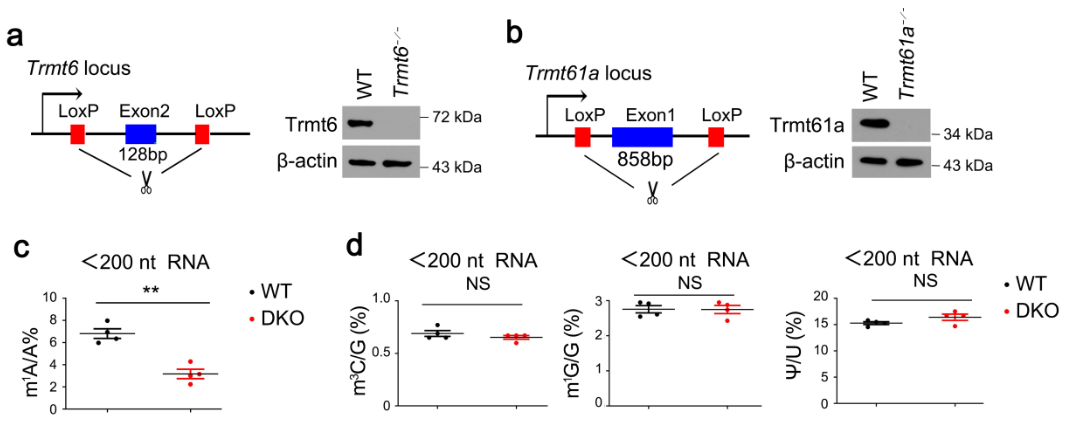


**Supplementary Figure 6. Trmt6/Trmt61a knockout strategy in mice.** (a, b) Diagram of generation strategies for *Trmt6*$^{flox/flox}$ and *Trmt61a*$^{flox/flox}$ using CRISPR-Cas9 technology. Gene knockout deficiencies in hepatocytes were confirmed by immunoblotting. n=5 biologically independent mice. (c, d) LC-MS/MS quantification of m$^1$A, m$^3$C, m$^1$G and Ψ levels in tRNA (＜200 nt) purified from indicated liver tissues, presented as percentage of unmodified A, G, G and U, respectively. Data are means ± SD. n=4. Exact *P* value for (c): 0.0081. ***P* < 0.01, and NS, not significant (*P* > 0.05) by two-tailed Student's *t* test.

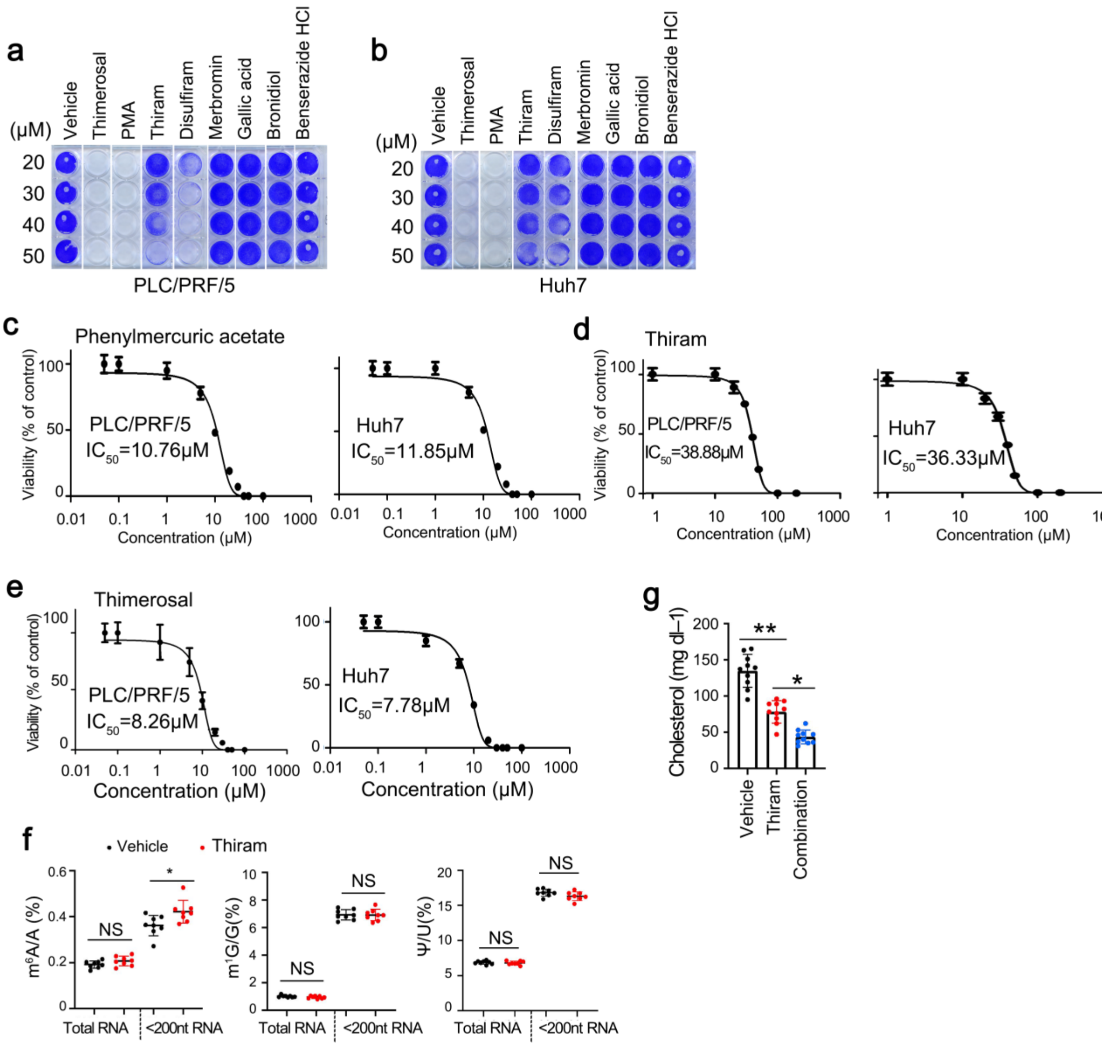


**Supplementary Figure 7. Inhibition of TRMT6/TRMT61A complex reduces liver CSC self-renewal and liver cancer development.** (a-b) Long-term colony formation assay was performed over 7–10 days on HCC cell lines PLC/PRF/5 and Huh7, treated with vehicle (DMSO) or indicated drugs of different concentrations. Cells were stained with crystal violet solution. (c-e) Dose–response curves of HCC cell lines to other candidate treatment, with an endpoint measurement at 120 h (means ± s.e.m., n = 5 biological repeats). (f) LC-MS/MS quantification of $m^6A/A$, $m^1G/G$ and $\Psi/U$ ratios in total RNAs and small RNA fraction (<200 nt) purified from liver CSCs treated with vehicle (DMSO) and thiram for 96 hr. Data are means ± SD. n=8. (g) Total serum cholesterol levels in PDC orthotopic engraft mouse models treated with vehicle, thiram, or combination. Exact *P* values from left to right: 0.0059, 0.023. **P* < 0.05, ***P* < 0.01; NS, not significant (*P* > 0.05) by two-tailed Student's *t* test.

**Supplementary Table 1. Clinical characteristics of HCC patients**

| Characteristics | Hepatocellular carcinoma (n=191) * |
|---|---|
| Sex | |
| Male | 117 (61) |
| Female | 74 (39) |
| Age (yr) | 57.2± 11.5 |
| ≤55 | 89 (47) |
| >55 | 102 (53) |
| HBV positive | 187 (98) |
| AFP (ug/L) | |
| ≤20 | 110 (58) |
| >20 | 81 (42) |
| Tumor size (cm) | |
| ≤5 | 99 (52) |
| >5 | 92 (48) |
| Cirrhosis | |
| With | 187 (98) |
| Without | 4 (2) |
| Differentiation | |
| Low | 48 (25) |
| Medium | 87 (46) |
| High | 56 (29) |

* Data are shown as means ± standard deviation (SD) or numbers (%).

**Supplementary Table 2. qRT-PCR primers used in this study**

| Gene | Forward | Reverse |
|---|---|---|
| *TRMT6* | 5'- TTGGGCGATTTTGAAGTGCG-3' | 5'- GGTGGAGAGGACTGGTTTCG -3' |
| *TRMT61A* | 5'-TGGCTAAAGAGCATTCTGCTAAG-3' | 5'-TGTGTAGTCGAAGCATGTTGTG-3' |
| *PPARD* | 5'-GATCCAGATTGATCGACCAGC-3' | 5'-GAGCCCATAGCGTCGTAGT-3' |
| *PPARA* | 5'-TGAAATCAGACTCCGACCAGA-3' | 5'-TGGCAAAGCAATGTCCATTAGTT-3' |
| *PPARG* | 5'-CTGCTCTACGACATGAACGG-3' | 5'-GAAGGTCCCTGATGTAGTCGAT-3' |
| 18S rRNA | 5'-AACCCGTTGAACCCCATT-3' | 5'-CCATCCAATCGGTAGTAGCG-3' |
| *ACTB* | 5'-CATGTACGTTGCTATCCAGGC-3' | 5'-CTCCTTAATGTCACGCACGAT-3' |
| *ABCA1* | 5'-ACCCACCCTATGAACAACATGA-3’ | 5’-GAGTCGGGTAACGGAAACAGG-3’ |
| *ABCG1* | 5'-ATTCAGGGACCTTTCCTATTCGG-3' | 5’-CTCACCACTATTGAACTTCCCG-3’ |
| *APOA1* | 5’-CCCTGGGATCGAGTGAAGGA-3’ | 5’-CTGGGACACATAGTCTCTGCC-3’ |
| *APOA2* | 5’-CTGTGCTACTCCTCACCATCT-3’ | 5’-CTCTCCACACATGGCTCCTTT-3’ |
| *APOA4* | 5’-CTCAAGGGACGCCTTACGC-3’ | 5’-GTCCTGAGCATAGGGAGCCA-3’ |
| *APOE* | 5’-GTTGCTGGTCACATTCCTGG-3’ | 5’-GCAGGTAATCCCAAAAGCGAC-3’ |
| *LDLR* | 5’-TCTGCAACATGGCTAGAGACT-3’ | 5’-TCCAAGCATTCGTTGGTCCC-3’ |
| *SCARB1* | 5’-CCTATCCCCTTCTATCTCTCCG-3’ | 5’-GGATGTTGGGCATGACGATGT-3’ |
| *OLR1* | 5’-ACCTTCCCCTCACACTCCTA-3’ | 5’-CTGTCTGTCTGTCTGTCCGT-3’ |
| *APOB* | 5’-TGCTCCACTCACTTTACCGTC-3’ | 5’-TAGCGTCCAGTGTGTACTGAC-3’ |
| *MYLIP* | 5’-GCAGGCGACTGGGAATCATAG-3’ | 5’-CGGTTTCTCAGGTTTAGCCAT-3’ |
| *MSR1* | 5’-GCAGTGGGATCACTTTCACAA-3’ | 5’-AGCTGTCATTGAGCGAGCATC-3’ |
| *VLDLR* | 5’-AGAAAAGCCAAATGTGAACCCT-3’ | 5’-CACTGCCGTCAACACAGTCT-3’ |
| *SREBF1* | 5’-CGGAACCATCTTGGCAACAGT-3’ | 5’-CGCTTCTCAATGGCGTTGT-3’ |
| *SREBF2* | 5’-CCTGGGAGACATCGACGAGAT-3’ | 5’-TGAATGACCGTTGCACTGAAG-3’ |
| *HMGCR* | 5’-TGATTGACCTTTCCAGAGCAAG-3’ | 5’-CTAAAATTGCCATTCCACGAGC-3’ |
| *HMGCS1* | 5’-GATGTGGGAATTGTTGCCCTT-3’ | 5’-ATTGTCTCTGTTCCAACTTCCAG-3’ |
| *HMGCS2* | 5’-GACTCCAGTGAAGCGCATTCT-3’ | 5’-CTGGGAAGTAGACCTCCAGG-3’ |
| *DHCR7* | 5’-GCTGCAAAATCGCAACCCAA-3’ | 5’-GCTCGCCAGTGAAAACCAGT-3’ |
| *CYP7A1* | 5'-GCAATTTGGTGCCAATCCTCT-3' | 5'-GCACAACACCTTATGGTATGACA-3' |
| *CYP7B1* | 5’-AAAACCACAAGTTGGGACACG-3’ | 5’-GAAGCTCAATGGGTATGTTGGAT-3’ |
| *CH25H* | 5’-ATCACCACATACGTGGGCTTT-3’ | 5’-GTCAGGGTGGATCTTGTAGCG-3’ |
| *CYP8B1* | 5’-GAAGCGCATGAGGACCAAG-3’ | 5’-TTGCATATTGCCCAAAGTCTAGT-3’ |
| *CYP27A1* | 5’-CGGCAACGGAGCTTAGAGG-3’ | 5’-GGCATAGCCTTGAACGAACAG-3’ |
| *CYP39A1* | 5’-CTTCAGCGGAAGAATTTGCGT-3’ | 5’-AAAGGTCATTCGGTTTCCCATAG-3’ |
| *CYP46A1* | 5’-TGTGTTTTTGGATTGGGCTAAGA-3’ | 5’-ACTCAGGACTCGTGACGATGA-3’ |
| *HSD3B1* | 5’-CACATGGCCCGCTCCATAC-3’ | 5’-GTGCCGCCGTTTTTCAGATTC-3’ |
| *HSD3B7* | 5’-CTGGTAGACGTGTTTGGCAGG-3’ | 5’-TGTTCCGGTCTGCACACAAG-3’ |
| *FABP1* | 5’-GTGTCGGAAATCGTGCAGAAT-3’ | 5’-GACTTTCTCCCCTGTCATTGTC-3’ |
| *NR1F1* | 5’-AGAACTATCAAAACAAGCGGGA-3’ | 5’- AAGGCACGGCACATTCTGATA-3’ |
| *NR1F3* | 5’- CGTCTGCAAGTCCTACAGGG-3’ | 5’- TCCACCACGTACTGAATGGC-3’ |
| *NR1H2* | 5’-AGAACTAATGATCCAGCAGTTGG-3’ | 5’-TTGCTTAGCGAAGTCCACGAT-3’ |
| *NR1H3* | 5’-TCTGGAGACATCTCGGAGGTA-3’ | 5’-GGCCCTGGAGAACTCGAAG-3’ |
| *NR1H4* | 5’-TGCAGATCAGACCGTGAATGA-3’ | 5’-TTGGTTGCCATTTCCGTCAAA-3’ |
| *GPR183* | 5’-GGGAAACTTACTAGCCTTGGTC-3’ | 5’-GCATCTCCGATTCTCCAGTCAA -3’ |

| | | |
|---|---|---|
| *CXCR2* | 5’- TACTGGCCTGCATCAGTGTG-3’ | 5’- CAGGCTGGGCTAACATTGGA-3’ |
| *ESR1* | 5’-CCCACTCAACAGCGTGTCTC-3’ | 5’-CGTCGATTATCTGAATTTGGCCT-3’ |
| *Gli1* | 5’-AGCGTGAGCCTGAATCTGTG-3’ | 5’-CAGCATGTACTGGGCTTTGAA-3’ |
| *Gli2* | 5’-CTGCCTCCGAGAAGCAAGAAG-3’ | 5’-GCATGGAATGGTGGCAAGAG-3’ |
| *Gli3* | 5’-TGGTTACATGGAGCCCCACTA-3’ | 5’-GAATCGGAGATGGATCGTAATGG-3’ |
| *PTCH1* | 5’-ACCCAGAAAGCAGACTACCC-3’ | 5’-ATCCTGAAGTCCCTGAAGCC-3’ |
| *HHIP* | 5’-TCTCAAAGCCTGTTCCACTCA-3’ | 5’-GCCTCGGCAAGTGTAAAAGAA-3’ |
| *BCL2* | 5’-GAGCTGGTGGTTGACTTTCTC-3’ | 5’-TCCATCTCCGATTCAGTCCCT-3’ |
| *MYC* | 5’-GGCTCCTGGCAAAAGGTCA-3’ | 5’-CTGCGTAGTTGTGCTGATGT-3’ |
| *TCF7* | 5’-CACGGGCAAACACTACGGT-3’ | 5’-TTGACCTTCGAGTGCTGATCC-3’ |
| *SOX4* | 5’-AGCGACAAGATCCCTTTCATTC-3’ | 5’-CGTTGCCGGACTTCACCTT-3’ |
| *CCND1* | 5’-CAATGACCCCGCACGATTTC-3’ | 5’-CATGGAGGGCGGATTGGAA-3’ |
| *HES1* | 5’-TCAACACGACACCGGATAAAC-3’ | 5’-GCCGCGAGCTATCTTTCTTCA-3’ |
| *HES6* | 5’-AGCAGGAGCCTGACTCAGTT-3’ | 5’-AGCTCCTGAACCATCTGCTC-3’ |
| *NRARP* | 5’-CACGGGGTGATCACTGCTAA-3’ | 5’-CGCTGGGCTACAGGTCAATA-3’ |
| *HEY1* | 5’-GTTCGGCTCTAGGTTCCATGT-3’ | 5’-CGTCGGCGCTTCTCAATTATTC-3’ |
| *mTrmt6* | 5’-GAGATTGCTGAACAAGCGGAT-3’ | 5’-AGGCTCTGAAGCTATCACAAGA-3’ |
| *mTrmt61a* | 5’-ATGAGTTTCGTGGCATACGAG-3’ | 5’-CCTCTGCTGCAAATCACCTT-3’ |
| *mPpard* | 5’-TCCATCGTCAACAAAGACGGG-3’ | 5’-ACTTGGGCTCAATGATGTCAC-3’ |
| *mActb* | 5’-TGTTACCAACTGGGACGACA-3’ | 5’--ACCAGAGGCATACAGGGACA3’ |

m: mouse

**Supplementary Table 3. ShRNAs, sgRNAs for human gene deficiency and guile RNAs for gene knockout in mice by CRISPR/Cas9 technology**

| Gene | Sequence (forward) | Sequence (reverse) |
|---|---|---|
| *TRMT6* shRNA#3 | 5'- GGGCACTGATAATCGAAAT -3' | |
| *TRMT6* shRNA#6 | 5'- GGTTCTACCTGGATAACGT -3' | |
| *TRMT61A* shRNA#1 | 5'- GGCACTCAGTTGACCTTAT -3' | |
| *TRMT61A* shRNA#2 | 5'- GCATACGAGGAGCTGATCAAG -3' | |
| *PPARD* sgRNA#1 | 5’- caccgGCTGCAAGATTCAGAAGAAC -3’ | 5'-aaacCCGACCAAAACGGATAGCTGc-3' |
| *SREBF2* sgRNA#3 | 5’-caccgGCTGCAATTTGTCAGTAAT-3’ | 5'-aaacAGTCAGGGAACTCTCCCACTTc-3' |
| | Guide RNA | |
| *Trmt6*$^{flox/flox}$-up | 5'- AACTAGAGAGAAGCAGGTTGGGG -3' | |
| *Trmt6*$^{flox/flox}$-down | 5'- ACGTCCGGCTTAGCTTCACAGGG -3' | |
| *Trmt61a*$^{flox/flox}$-up | 5'- GGAGCACGCAAAGGACAGCGCGG -3' | |
| *Trmt61a*$^{flox/flox}$-down | 5'- TGGTATGAGCTCAGATAAGACGG -3' | |
| *Ppard*$^{-/-}$ | 5'- GCCACAGGAGGAGACCCCTG -3' | |
