# Supplementary material for: N1-methyladenosine methylation in tRNA drives liver tumourigenesis by regulating cholesterol metabolism
Source: Nat Commun. 2021 Nov 2;12:6314. doi: 10.1038/s41467-021-26718-6 (PMC8563902; doi:10.1038/s41467-021-26718-6)
Supplement: Supplementary file 2 — Reporting Summary [file 41467_2021_26718_MOESM2_ESM.pdf]

nature research

Corresponding author(s): Zusen Fan
Chengqi Yi
Yong Tian
Yanying Wang

Last updated by author(s): Jul 18, 2021

Reporting Summary

Nature Research wishes to improve the reproducibility of the work that we publish. This form provides structure for consistency and transparency in reporting. For further information on Nature Research policies, see our Editorial Policies and the Editorial Policy Checklist.

Statistics

For all statistical analyses, confirm that the following items are present in the figure legend, table legend, main text, or Methods section.

n/a	Confirmed	
☐	☒	The exact sample size (n) for each experimental group/condition, given as a discrete number and unit of measurement
☐	☒	A statement on whether measurements were taken from distinct samples or whether the same sample was measured repeatedly
☐	☒	The statistical test(s) used AND whether they are one- or two-sided<br>*Only common tests should be described solely by name; describe more complex techniques in the Methods section.*
☒	☐	A description of all covariates tested
☒	☐	A description of any assumptions or corrections, such as tests of normality and adjustment for multiple comparisons
☐	☒	A full description of the statistical parameters including central tendency (e.g. means) or other basic estimates (e.g. regression coefficient) AND variation (e.g. standard deviation) or associated estimates of uncertainty (e.g. confidence intervals)
☐	☒	For null hypothesis testing, the test statistic (e.g. F, t, r) with confidence intervals, effect sizes, degrees of freedom and P value noted<br>*Give P values as exact values whenever suitable.*
☒	☐	For Bayesian analysis, information on the choice of priors and Markov chain Monte Carlo settings
☒	☐	For hierarchical and complex designs, identification of the appropriate level for tests and full reporting of outcomes
☒	☐	Estimates of effect sizes (e.g. Cohen's d, Pearson's r), indicating how they were calculated

Our web collection on statistics for biologists contains articles on many of the points above.

Software and code

Policy information about availability of computer code

Data collection	Custom codes were generated for the analysis of the ribosome profiling data, m1A-seq, RNA-seq, proteomics analysis, and untargeted lipid metabolomics assays.
Data analysis	Prism 8 software from GraphPad 8 (GraphPad Software Inc., La Jolla, CA), FlowJo software version 9.9, SOAPnuke (v1.5.2), HISAT2 (v2.0.4), Bowtie2 (v2.2.5), RSEM (v1.2.12), pheatmap (v1.0.8), DESeq2(v1.4.5), BWA-MEM (version 0.7.17-r1188),Q Exactive mass spectrometer (Thermo Scientific), Proteome Discovery version 2.2.0.388, Living Image 4.3 software (Perkin Elmer), Progenesis QI (version 2.2, hereinafter referred to as QI) , SPSS 13.0.

For manuscripts utilizing custom algorithms or software that are central to the research but not yet described in published literature, software must be made available to editors and reviewers. We strongly encourage code deposition in a community repository (e.g. GitHub). See the Nature Research guidelines for submitting code & software for further information.

Data

Policy information about availability of data

All manuscripts must include a data availability statement. This statement should provide the following information, where applicable:
- Accession codes, unique identifiers, or web links for publicly available datasets
- A list of figures that have associated raw data
- A description of any restrictions on data availability

All data supporting the findings of this study are available within the paper. Raw and processed data from the m1A RNA sequencing and ribosome profiling data of samples have been deposited to the NCBI Gene Expression Omnibus (GEO) under accession number GSE147840. Proteomics data have been deposited in the PRIDE database (www.ebi.ac.uk/pride/archive) with accession number PXD019576. Untargeted lipid metabolomics assay can be obtained from the MetaboLights database with the identifier MTBLS1781 (www.ebi.ac.uk/metabolights/MTBLS1781). Gene expression profiles by RNA-seq can be obtained from Gene Expression

Omnibus with accession number GSE152108.
A list of figures that have associated raw data
Fig.3B, 3D,3E,3F, 4A, 4B. Support data Fig. 3D, 3E,3F,3I,5A, 5B,5C, 5D, 5E.

Field-specific reporting

Please select the one below that is the best fit for your research. If you are not sure, read the appropriate sections before making your selection.

☒ Life sciences ☐ Behavioural & social sciences ☐ Ecological, evolutionary & environmental sciences

For a reference copy of the document with all sections, see nature.com/documents/nr-reporting-summary-flat.pdf

Life sciences study design

All studies must disclose on these points even when the disclosure is negative.

Sample size	For the m1A, TRMT6, and PPARD expression analysis of paired HCC tissues, the sample size were chosen similar to the TCGA project. For the PDX experiment, at least 5 biological replicates were performed. For the cell line validation experiments, at least 3 biological replicates were performed. No statistical method was used to pre-determine the sample size. 5-12 mice per group for xenografts or orthotopical transplantation. Sample size of mice was based on previous experience with the mouse models (Anirudh et al., 2012).
Data exclusions	No tissues/tumors/in vitro cell line samples were excluded from analysis. Animals were excluded if dying because of non-cancer related causes.
Replication	All the in vitro experiments (except RNA sequencing , untargeted lipid metabolomics assays, tissue mirroarray assays) were repeated in at least 3 independent experiments and showed comparable results between experiments. All in vivo data were repeated in at least 2 independent experiments. All details on biological and technical replicates are provided in the text and/or figure legends.
Randomization	The HCC paired tumor and adjacent peri-tumor tissue samples were collected from hospital and were set for m1A, RNA, and protein expression analysis successively, without randomization process. Samples were allocated into experimental groups as described in Methods section. Randomization was performed for mice. For orthotopic transplantation, after tumor establishment, mice were randomly assigned to treatment with vehicle or drugs.
Blinding	Assessment of IHC counts were performed by 2 or more independent researchers in a blinded fashion. Investigators were blinded while assessing outcomes during animal experiments.

Reporting for specific materials, systems and methods

We require information from authors about some types of materials, experimental systems and methods used in many studies. Here, indicate whether each material, system or method listed is relevant to your study. If you are not sure if a list item applies to your research, read the appropriate section before selecting a response.

Materials & experimental systems

n/a	Involved in the study	
☐	☒	Antibodies
☐	☒	Eukaryotic cell lines
☒	☐	Palaeontology and archaeology
☐	☒	Animals and other organisms
☐	☒	Human research participants
☒	☐	Clinical data
☒	☐	Dual use research of concern

Methods

n/a	Involved in the study	
☒	☐	ChIP-seq
☐	☒	Flow cytometry
☒	☐	MRI-based neuroimaging

Antibodies

Antibodies used	Anti-1-methyladenosine (m1A) (D345-3) antibody was purchased from MBL (Japan) (1:10000 dilution for IHC and 1:1500 dilution for dot blot). Anti-6-methyladenosine antibody (202 003) was from Synaptic Systems (Germany) (1:200 dilution). Phycoerythrin (PE)-anti-human CD133 (clone no. REA820, 130-112-195) was purchased from Miltenyi Biotec (Germany) (1:50 dilution). PE-anti-human IgG (12-4998-82) (1:400 dilution), fluorescein isothiocyanate (FITC)-anti-human CD13 (clone no. WM-15, 11-0138-42) (1:400 dilution), FITC-anti-human IgG (31529)(1:400 dilution) were purchased from eBioscience (San Diego, USA). Anti-TRMT6 (PA5-61409) (1:200 dilution for IHC and 1:1000 dilution for WB) for human IHC and immunoblotting detection, anti-PPARdelta(PA1-823A) (1:500 dilution), anti-SREBF2 (PA1-338) (1:500 dilution) antibodies were from Thermo Fisher Scientific (Waltham, USA). Anti-TRMT6 (16727-1-AP) for mouse IHC (1:200 dilution) and immunoblotting (1:500 dilution)detection, anti-FABP1 (13626-1-AP), anti-APOA2 (16845-1-AP) (1:1000 dilution), anti-PPARα (15540-1-AP) (1:500 dilution), anti-PPARγ (16643-1-AP) (1:1000 dilution) antibodies were from Proteintech (USA). Anti-ZNF780A (NBP1-79357) (1:1000 dilution), anti-ZNF821 (NBP2-82055) (1:2000 dilution), anti-HMGCR (NBP2-66888) (1:1000 dilution) were from Novus Biologicals (USA) . Anti-HMGCS2 antibody (#20940S) was from Cell Signaling Technology, Inc. (USA) (1:1000 dilution). Anti-TRMT61A (SAB2700607) (1:1000 dilution) and anti-β-actin (1:8000 dilution)antibodies

were from Sigma-Aldrich (USA). HRP-conjugated secondary antibodies were from ZSGB-BIO (Beijing, China) (1:5000 dilution). Secondary antibodies conjugated with Alexa-594, Alexa-488, or Alexa-649 were purchased from Molecular probes Inc (Eugene, USA) (1:500 dilution).

Validation

1, Anti-1-methyladenosine (m1A): Application statement in manufacturer's website as following: this antibody is validated for use in IHC, ICC/IF, RNA IP. http://www.mbl-chinawide.cn/search01?keyword=d345-3.
2, Anti-6-methyladenosine: Application statement in manufacturer's website as following: this antibody is validated for use in IHC, ICC/IF, IP. https://www.sysy.com/product/202003#list.
3, Phycoerythrin (PE)-anti-human CD133: Application statement in manufacturer's website as following: this antibody is validated for use in FC, MICS, IF, IHC. https://www.miltenyibiotec.com/CN-en/products/cd133-2-antibody-anti-human-reafinity-rea820.html#pe:100-tests-in-200-ul.
4, Fluorescein isothiocyanate (FITC)-anti-human CD13 : Application statement in manufacturer's website as following: this antibody is validated for use in FC. https://www.thermofisher.cn/cn/zh/antibody/product/CD13-Antibody-clone-WM-15-WM15-Monoclonal/11-0138-42.
5, Anti-TRMT6: Application statement in manufacturer's website as following: this antibody is validated for use in WB,IHC,ICC/IF. https://www.thermofisher.cn/cn/zh/antibody/product/TRMT6-Antibody-Polyclonal/PA5-61409.
6, Anti-PPAR delta : Application statement in manufacturer's website as following: this antibody is validated for use in WB,IHC,ICC/IF,IP,ChIP. https://www.thermofisher.cn/cn/zh/antibody/product/PPAR-delta-Antibody-Polyclonal/PA1-823A.
7, Anti-SREBF2: Application statement in manufacturer's website as following: this antibody is validated for use in WB,ICC/IF. https://www.thermofisher.cn/cn/zh/antibody/product/SREBP2-Antibody-Polyclonal/PA1-338
8, Anti-TRMT6: Application statement in manufacturer's website as following: this antibody is validated for use in WB,IHC. https://www.ptgcn.com/products/TRMT6-Antibody-16727-1-AP.html.
9, Anti-FABP1: Application statement in manufacturer's website as following: this antibody is validated for use in WB,IHC,ELISA. https://www.ptgcn.com/products/FABP1-Antibody-13626-1-AP.html
10, Anti-APOA2: Application statement in manufacturer's website as following: this antibody is validated for use in IF, IHC, IP, WB,ELISA. https://www.ptgcn.com/products/APOA2-Antibody-16845-1-AP.html.
11, Anti-ZNF780A: Application statement in manufacturer's website as following: this antibody is validated for use in WB. https://www.novusbio.com/products/znf780a-antibody_nbp1-79357?utm_source=linscott&utm_medium=referral&utm_campaign=product&utm_term=primaryantibodies#datasheet.
12, Anti-ZNF821: Application statement in manufacturer's website as following: this antibody is validated for use in WB, ELISA, ICC/IF. https://www.novusbio.com/products/znf821-antibody_nbp2-82055#datasheet
13, Anti-HMGCS2: Application statement in manufacturer's website as following: this antibody is validated for use in IP, WB. https://www.cellsignal.cn/products/primary-antibodies/hmgcs2-d3u1a-rabbit-mab/20940?site-search-type=Products&N=4294956287&Ntt=20940s&fromPage=plp&_requestid=1334528.
14, Anti-HMGCR: Application statement in manufacturer's website as following: this antibody is validated for use in WB. https://www.novusbio.com/products/hmg-coa-reductase-hmgcr-antibody-jf0981_nbp2-66888
15, Anti-TRMT61A: Application statement in manufacturer's website as following: this antibody is validated for use in WB. https://www.sigmaaldrich.cn/CN/zh/product/sigma/sab2700607?context=product.
16, Anti-PPARα: Application statement in manufacturer's website as following: this antibody is validated for use in WB. https://www.ptgcn.com/products/PPARA-Antibody-15540-1-AP.htm
17, Anti-PPARγ: Application statement in manufacturer's website as following: this antibody is validated for use in CHIP, CoIP, IF, IHC, WB. https://www.ptgcn.com/products/PPARG-Antibody-16643-1-AP.htm

Eukaryotic cell lines

Policy information about cell lines

Cell line source(s)	Cell lines source is stated in the Method section of the manuscript. Human hepatocellular carcinoma (HCC) cell lines Hep3B, Huh7, PLC/PRF/5 and Human 293T cells were obtained from ATCC
Authentication	The HCC cell lines were authenticated by applying short tandem-repeat (STR) DNA profiling.
Mycoplasma contamination	Mycoplasma contamination was excluded using PCR dectection. Only cells found negative for mycoplasma were used in this study.
Commonly misidentified lines (See ICLAC register)	No commonly misidentified lines were used in this study.

Animals and other organisms

Policy information about studies involving animals; ARRIVE guidelines recommended for reporting animal research

Laboratory animals	All mice were maintained in pathogen-free conditions at ambient temperature 20 to 22 °C, humidity 50-60% under standard 12 hr light-dark cycle, fed stand rodent chow and water, and were used for experiments at age of 8–10 weeks.
Wild animals	The study did not involve wild animals.
Field-collected samples	The study did not involve field-involved samples.
Ethics oversight	Mouse experiments were approved by the Institutional Animal Care and Use Committees at the Institute of Biophysics, Chinese Academy of Sciences.

Note that full information on the approval of the study protocol must also be provided in the manuscript.

Human research participants

Policy information about studies involving human research participants

Population characteristics	The paired HCC samples used in this study were obtained from the PLA General Hospital in China. There are no biases in the selection of patients. All samples are renamed with codes (such as LC (tumor) #1, #2, LN (peri-tumor) #1, #2, and so on) instead of the patient's name. Surgically resected primary tumor tissues and paired peri-tumor liver tissues were selected from 191 HCC patients who had not undergone prior chemotherapy or radiotherapy. Of these cases, 187 had an HBV-infection background. Clinical information—including gender, age, etiology, status of liver cirrhosis, clinical grade, tumor number, size, status of microscopic vascular invasion, serum AFP. Information on the patient cohort are provided in Supplemental Tables S1.
Recruitment	191 HCC patients who had not undergone prior chemotherapy or radiotherapy. Of these cases, 187 had an HBV-infection background. There are no biases on the selection of patients. Detailed information is provided in the Methods section.
Ethics oversight	All tissue samples were obtained from consenting patients and approved by Research Ethics Committee at the PLA hospital and the Institutional Review Board of the Institute of Biophysics, Chinese Academy of Sciences. Described in Methods.

Note that full information on the approval of the study protocol must also be provided in the manuscript.

Flow Cytometry

Plots

Confirm that:

- [x] The axis labels state the marker and fluorochrome used (e.g. CD4-FITC).
- [x] The axis scales are clearly visible. Include numbers along axes only for bottom left plot of group (a 'group' is an analysis of identical markers).
- [x] All plots are contour plots with outliers or pseudocolor plots.
- [x] A numerical value for number of cells or percentage (with statistics) is provided.

Methodology

Sample preparation	For liver CSCs sorting, cocktail PE-conjugated anti-human CD133 and FITC-conjugated anti-human CD13 antibodies or corresponding isotype control antibodies were incubated with HCC primary cells or cell lines.
Instrument	FACS Aria III (BD Immunocytometry Systems, San Jose, CA, USA).
Software	FlowJo, BD FACS software
Cell population abundance	The content of CSC (CD13+CD133+, double positive) and non-CSC (CD13-CD133-, double negative) was assessed relative to total alive hepatocytes. The purity of the samples were validated by confocal microscopy (Zeiss)
Gating strategy	Gating strategy is provided in the manuscript.

- [x] Tick this box to confirm that a figure exemplifying the gating strategy is provided in the Supplementary Information.
