# Supplementary figures and images for: N1-methyladenosine methylation in tRNA drives liver tumourigenesis by regulating cholesterol metabolism
Source: Nat Commun. 2021 Nov 2;12:6314. doi: 10.1038/s41467-021-26718-6 (PMC8563902; doi:10.1038/s41467-021-26718-6)

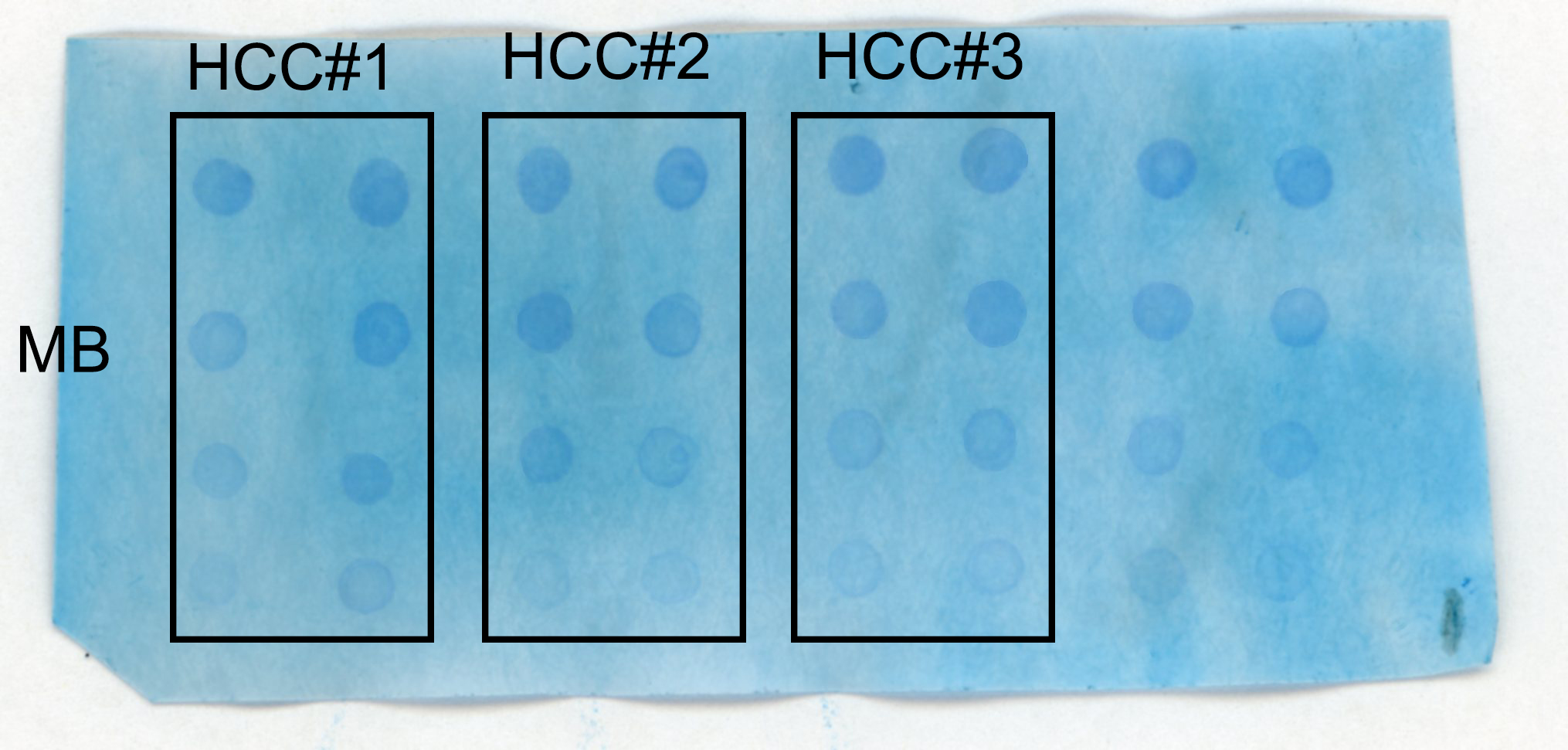

Supplement: Supplementary file 3 — Source Data [file 41467_2021_26718_MOESM3_ESM.zip › uncropped versions of any gels or blots/Fig.1F HCC1-3 MB.jpg]

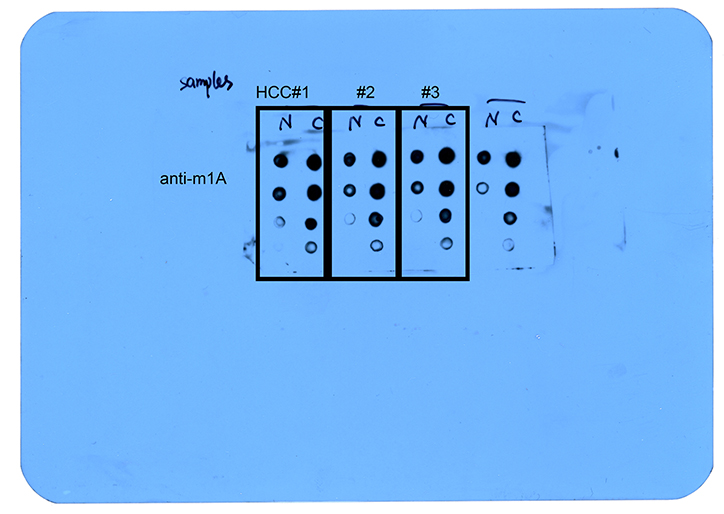

Supplement: Supplementary file 3 — Source Data [file 41467_2021_26718_MOESM3_ESM.zip › uncropped versions of any gels or blots/Fig.1F-HCC1-3 m1A.jpg]

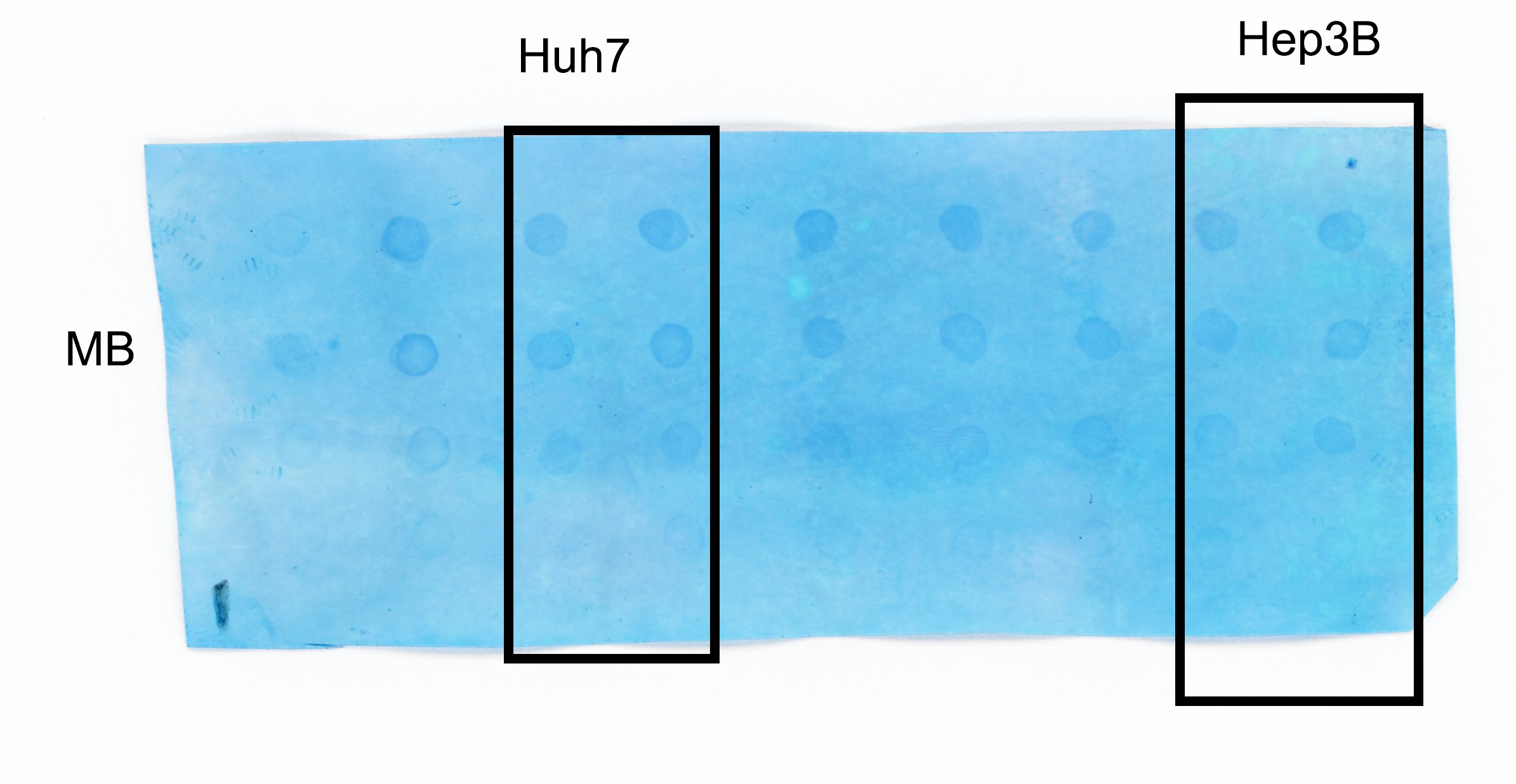

Supplement: Supplementary file 3 — Source Data [file 41467_2021_26718_MOESM3_ESM.zip › uncropped versions of any gels or blots/Fig.1F-Hep3B Huh7-MB.jpg]

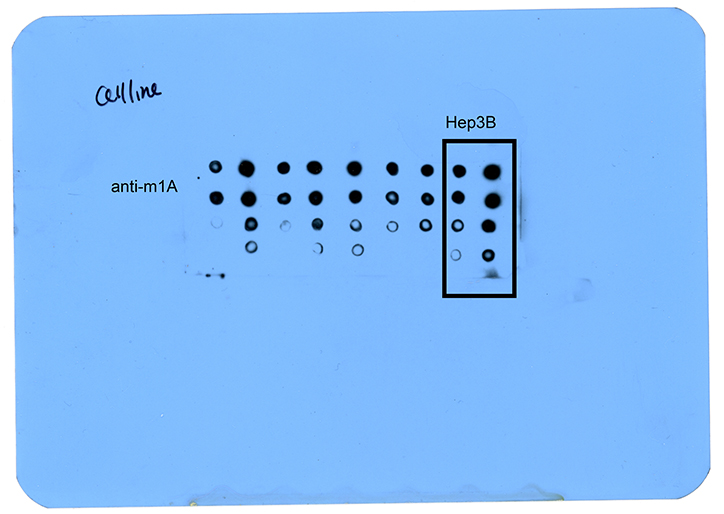

Supplement: Supplementary file 3 — Source Data [file 41467_2021_26718_MOESM3_ESM.zip › uncropped versions of any gels or blots/Fig.1F-Hep3B m1A.jpg]

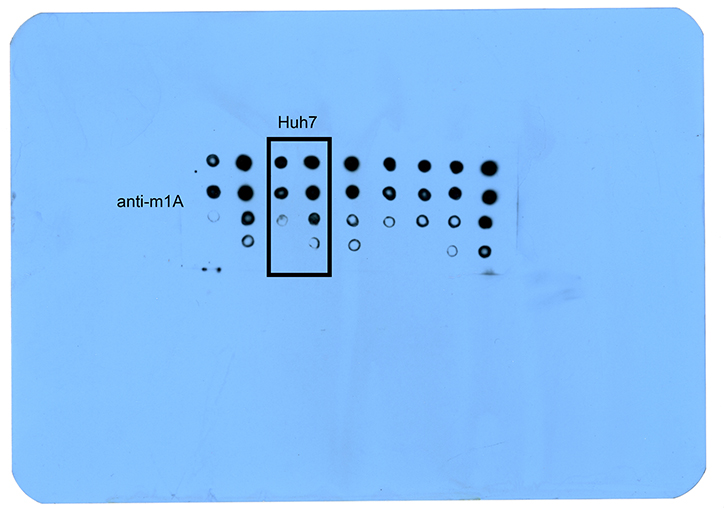

Supplement: Supplementary file 3 — Source Data [file 41467_2021_26718_MOESM3_ESM.zip › uncropped versions of any gels or blots/Fig.1F-huh7 m1A.jpg]

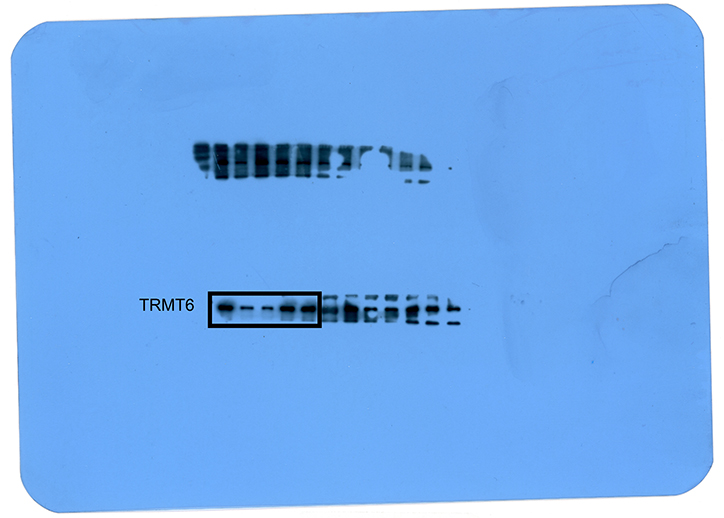

Supplement: Supplementary file 3 — Source Data [file 41467_2021_26718_MOESM3_ESM.zip › uncropped versions of any gels or blots/Fig.2B TRMT6.jpg]

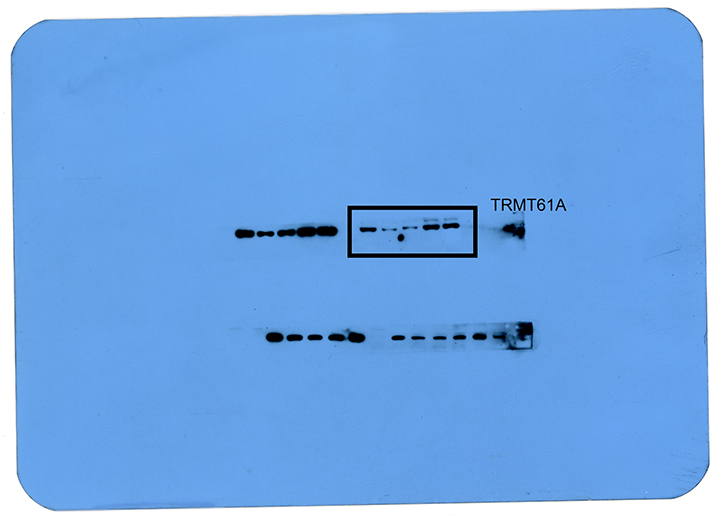

Supplement: Supplementary file 3 — Source Data [file 41467_2021_26718_MOESM3_ESM.zip › uncropped versions of any gels or blots/Fig.2B TRMT61A.jpg]

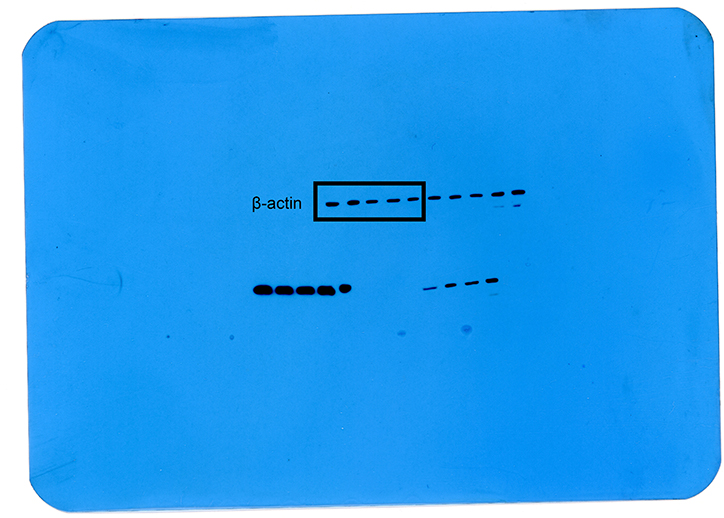

Supplement: Supplementary file 3 — Source Data [file 41467_2021_26718_MOESM3_ESM.zip › uncropped versions of any gels or blots/Fig.2B actin.jpg]

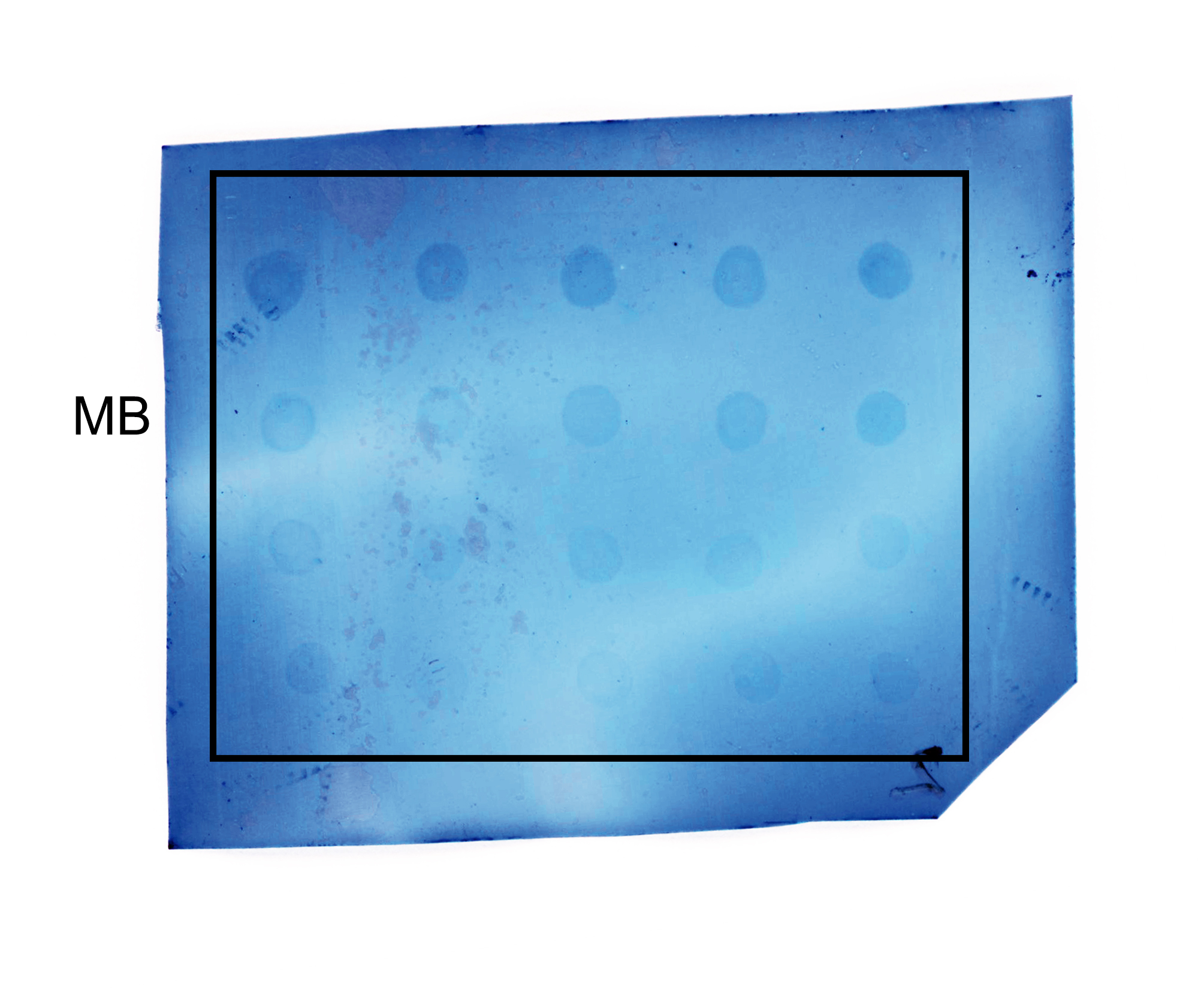

Supplement: Supplementary file 3 — Source Data [file 41467_2021_26718_MOESM3_ESM.zip › uncropped versions of any gels or blots/Fig.2C-MB.jpg]

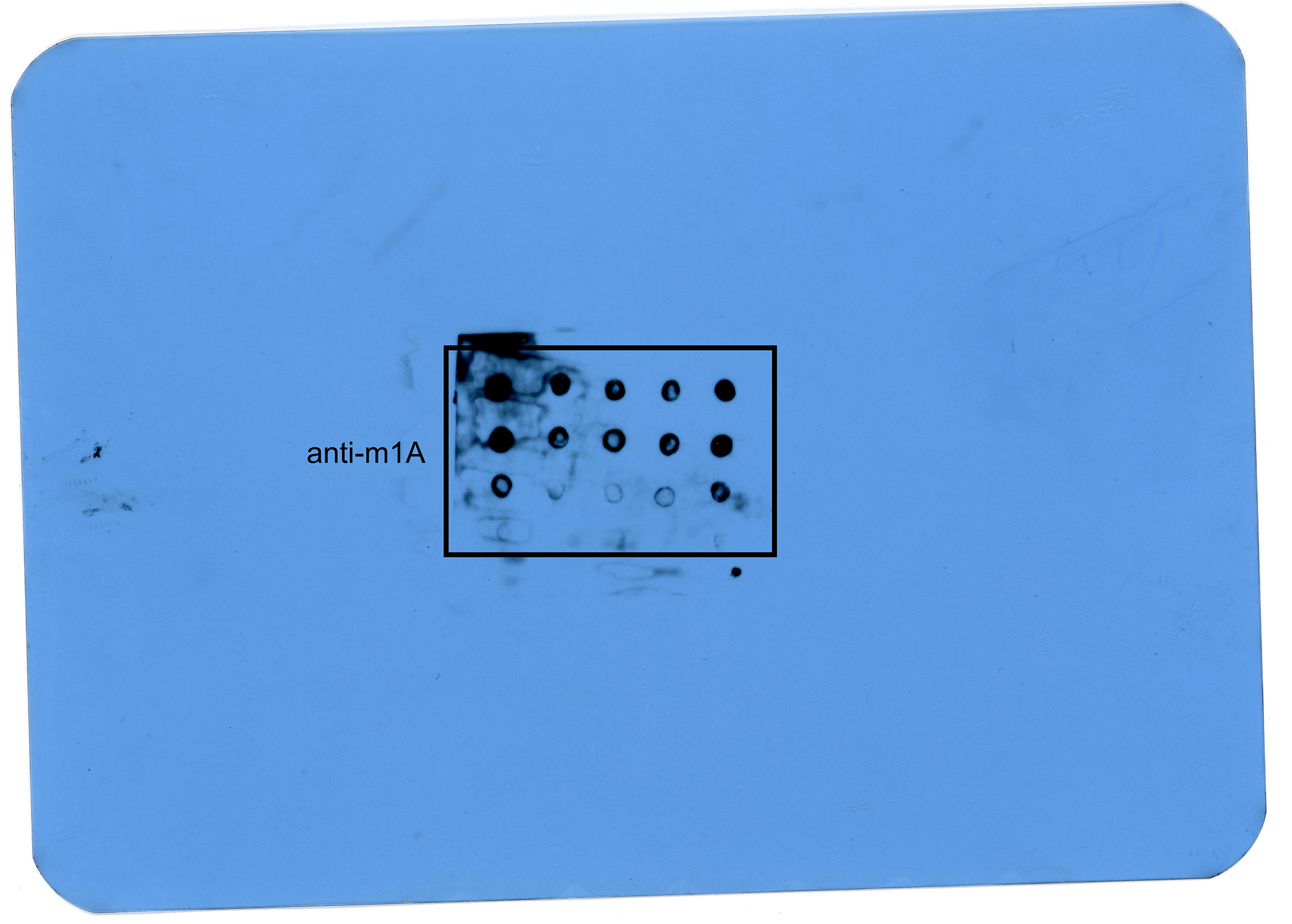

Supplement: Supplementary file 3 — Source Data [file 41467_2021_26718_MOESM3_ESM.zip › uncropped versions of any gels or blots/Fig.2C-m1A.jpg]

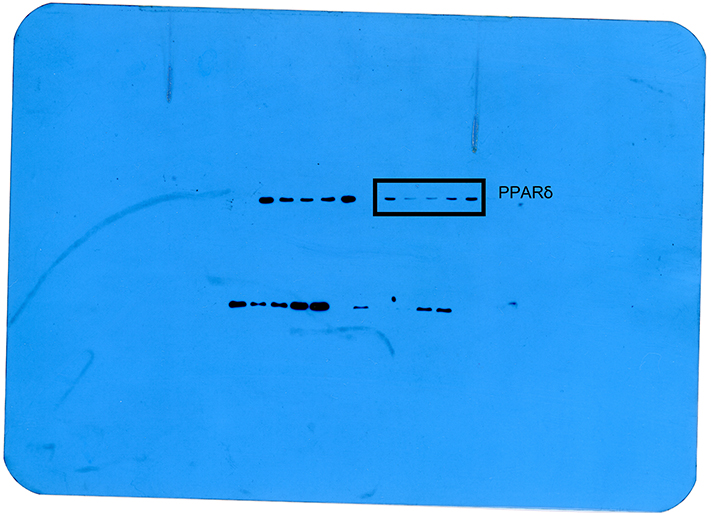

Supplement: Supplementary file 3 — Source Data [file 41467_2021_26718_MOESM3_ESM.zip › uncropped versions of any gels or blots/Fig.3F PPARẟ.jpg]

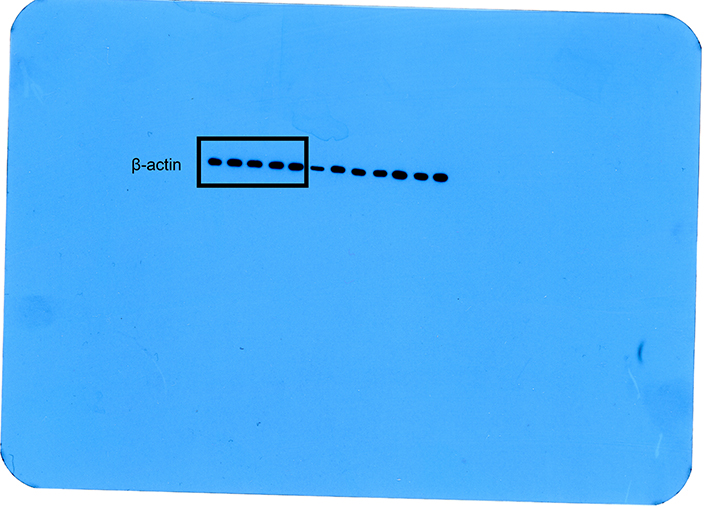

Supplement: Supplementary file 3 — Source Data [file 41467_2021_26718_MOESM3_ESM.zip › uncropped versions of any gels or blots/Fig.3F actin.jpg]

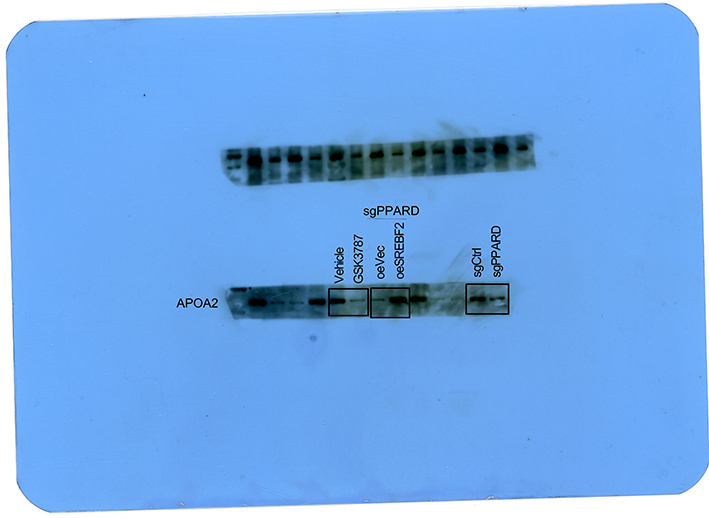

Supplement: Supplementary file 3 — Source Data [file 41467_2021_26718_MOESM3_ESM.zip › uncropped versions of any gels or blots/Fig.4D APOA2.jpg]

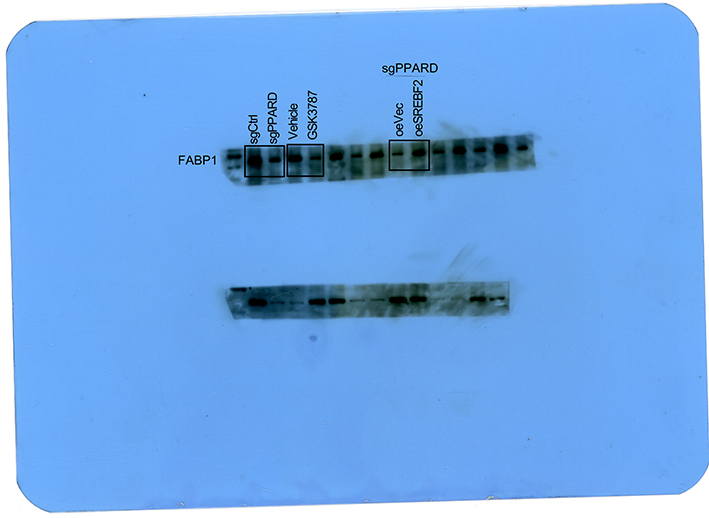

Supplement: Supplementary file 3 — Source Data [file 41467_2021_26718_MOESM3_ESM.zip › uncropped versions of any gels or blots/Fig.4D FABP1.jpg]

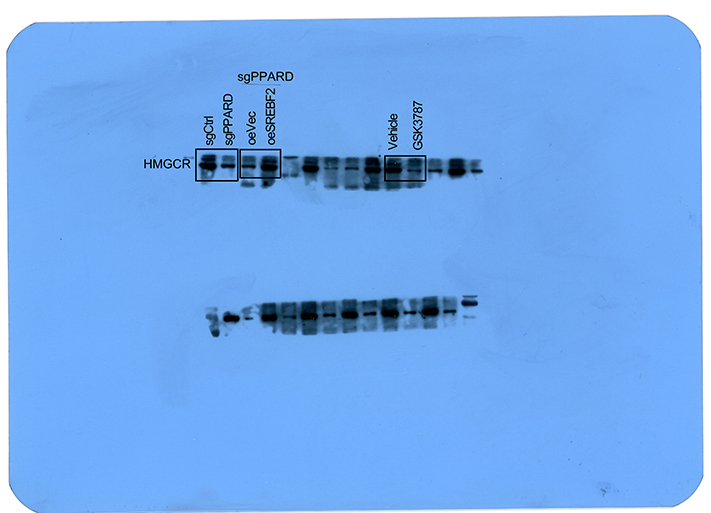

Supplement: Supplementary file 3 — Source Data [file 41467_2021_26718_MOESM3_ESM.zip › uncropped versions of any gels or blots/Fig.4D HMGCR.jpg]

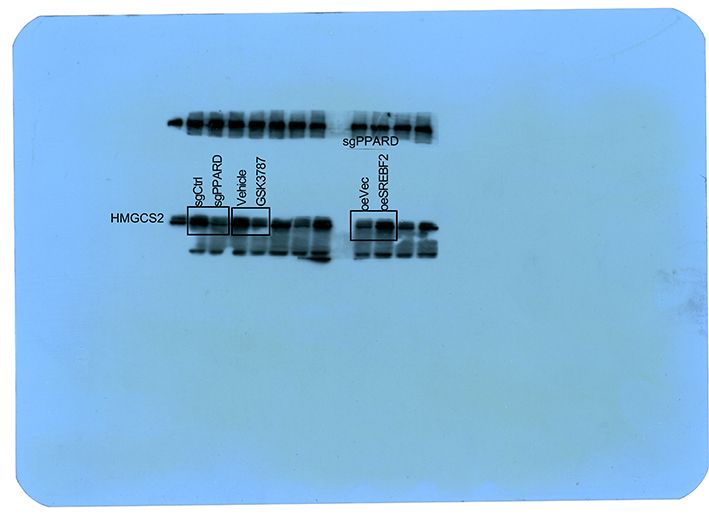

Supplement: Supplementary file 3 — Source Data [file 41467_2021_26718_MOESM3_ESM.zip › uncropped versions of any gels or blots/Fig.4D HMGCS2.jpg]

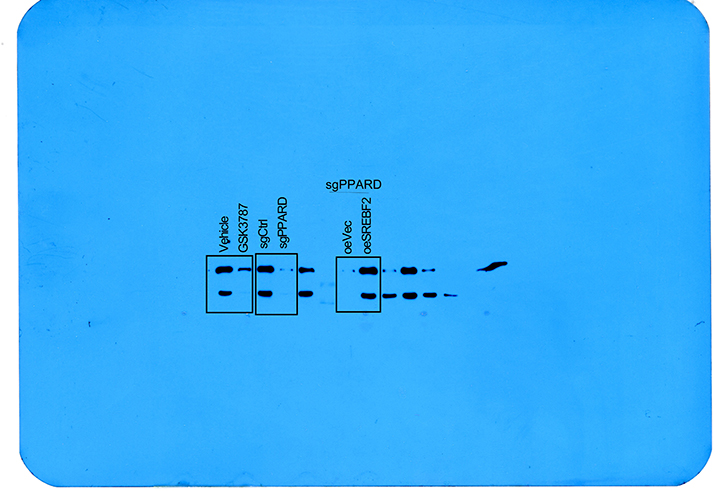

Supplement: Supplementary file 3 — Source Data [file 41467_2021_26718_MOESM3_ESM.zip › uncropped versions of any gels or blots/Fig.4D SREBF2.jpg]

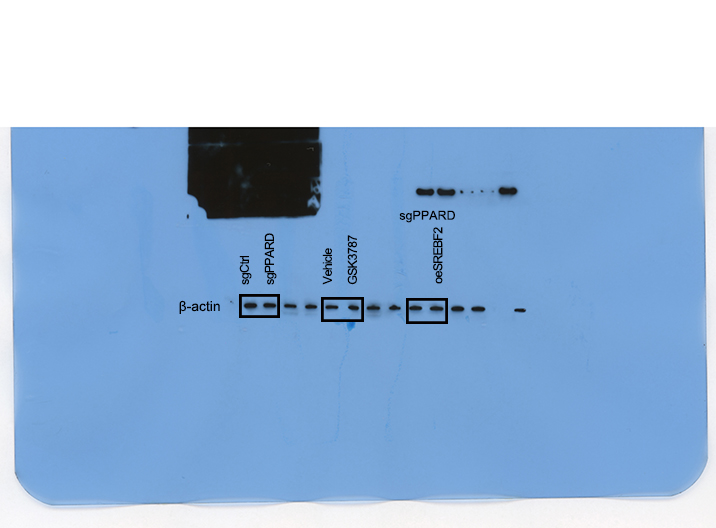

Supplement: Supplementary file 3 — Source Data [file 41467_2021_26718_MOESM3_ESM.zip › uncropped versions of any gels or blots/Fig.4D actin.jpg]

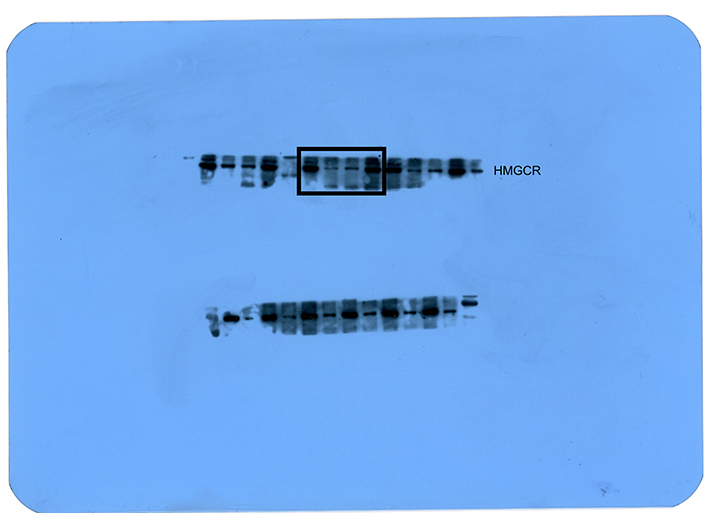

Supplement: Supplementary file 3 — Source Data [file 41467_2021_26718_MOESM3_ESM.zip › uncropped versions of any gels or blots/Fig.6D HMGCR.jpg]

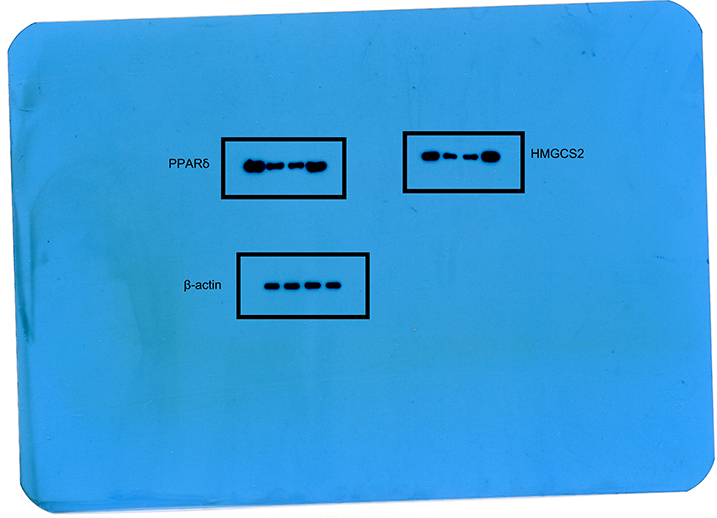

Supplement: Supplementary file 3 — Source Data [file 41467_2021_26718_MOESM3_ESM.zip › uncropped versions of any gels or blots/Fig.6D.jpg]

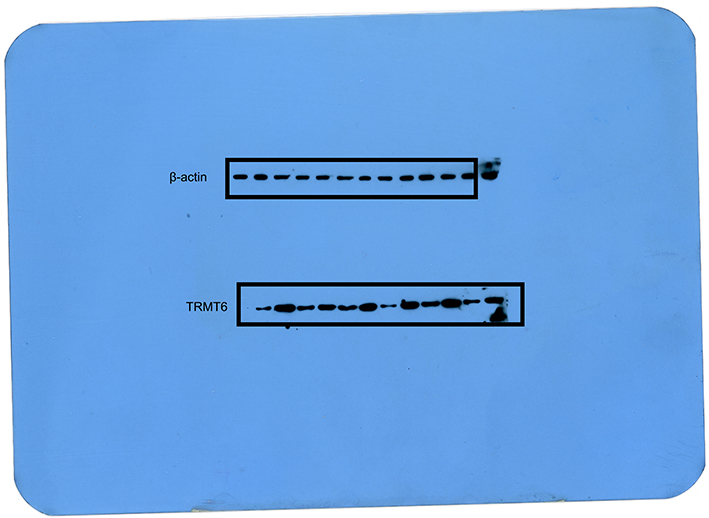

Supplement: Supplementary file 3 — Source Data [file 41467_2021_26718_MOESM3_ESM.zip › uncropped versions of any gels or blots/Fig.S2B TRMT6 actin.jpg]

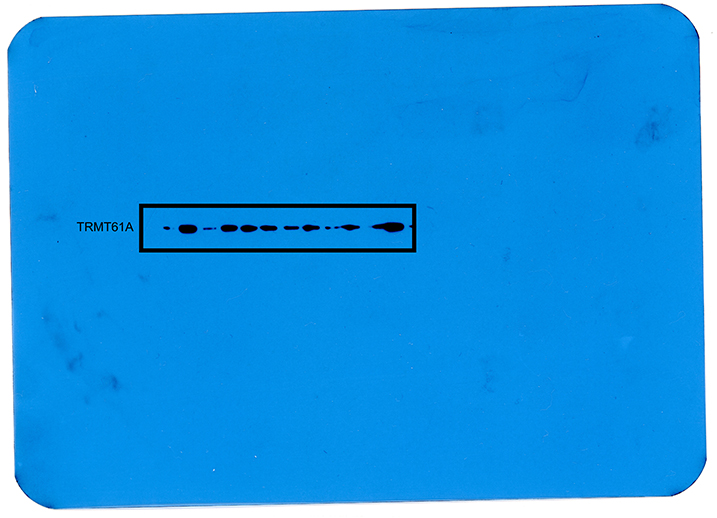

Supplement: Supplementary file 3 — Source Data [file 41467_2021_26718_MOESM3_ESM.zip › uncropped versions of any gels or blots/Fig.S2B TRMT61A.jpg]

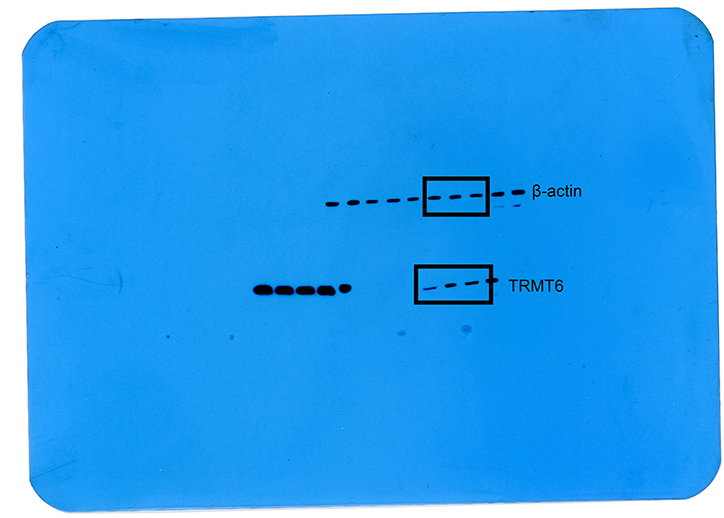

Supplement: Supplementary file 3 — Source Data [file 41467_2021_26718_MOESM3_ESM.zip › uncropped versions of any gels or blots/Fig.S2K TRMT6 actin.jpg]

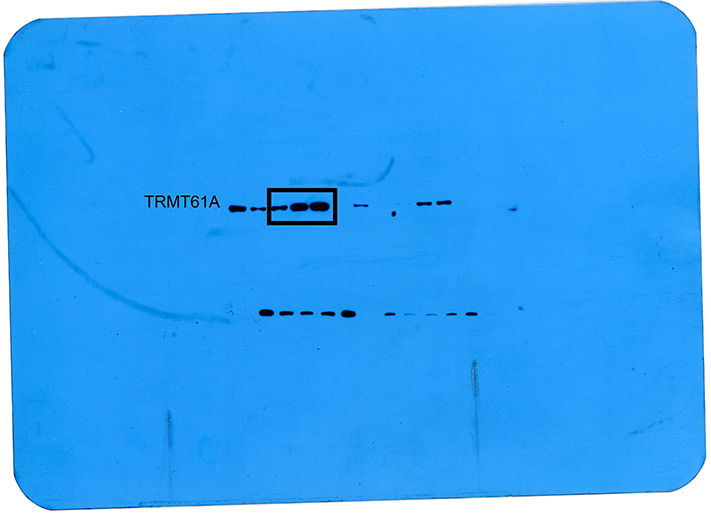

Supplement: Supplementary file 3 — Source Data [file 41467_2021_26718_MOESM3_ESM.zip › uncropped versions of any gels or blots/Fig.S2K TRMT61A.jpg]

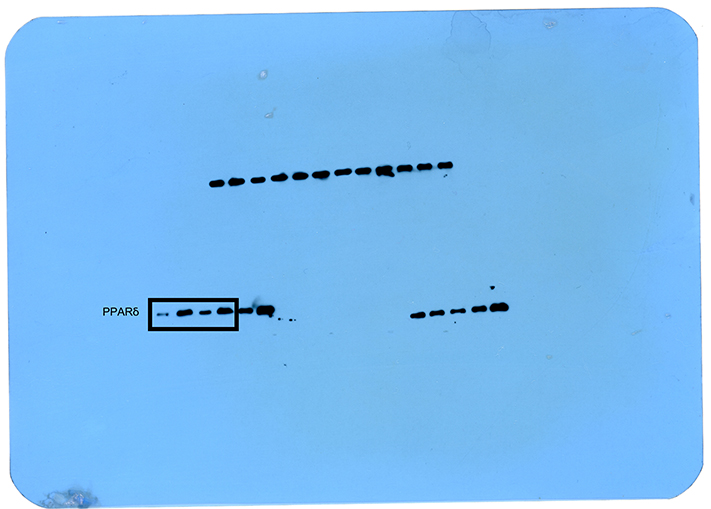

Supplement: Supplementary file 3 — Source Data [file 41467_2021_26718_MOESM3_ESM.zip › uncropped versions of any gels or blots/Fig.S3I PPARẟ.jpg]

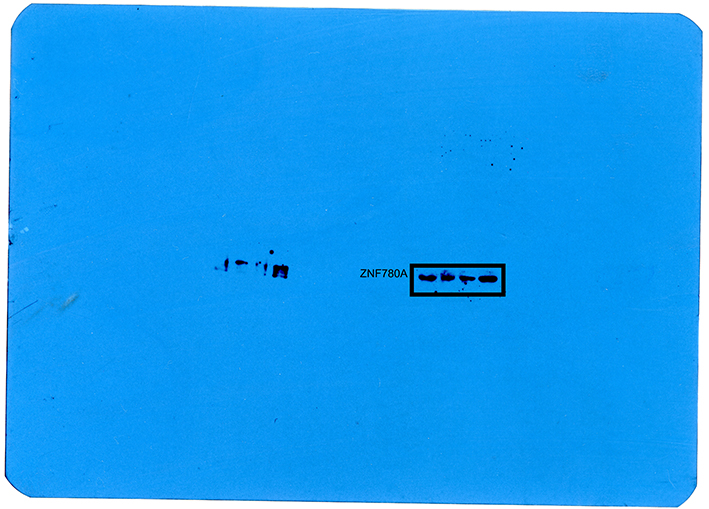

Supplement: Supplementary file 3 — Source Data [file 41467_2021_26718_MOESM3_ESM.zip › uncropped versions of any gels or blots/Fig.S3I ZNF780A.jpg]

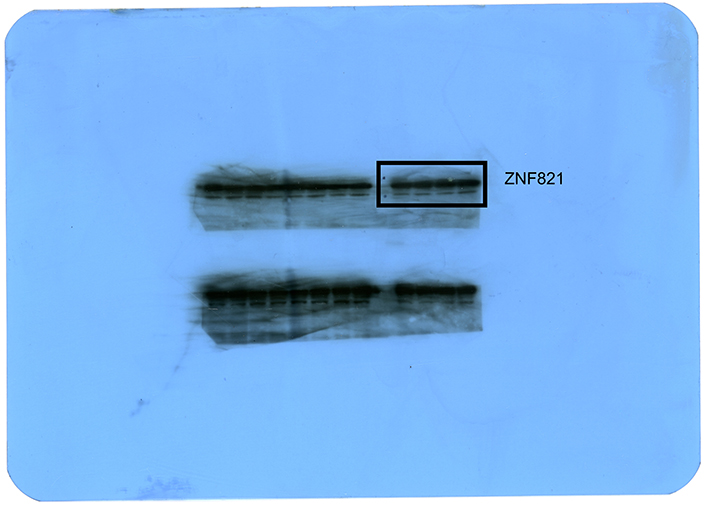

Supplement: Supplementary file 3 — Source Data [file 41467_2021_26718_MOESM3_ESM.zip › uncropped versions of any gels or blots/Fig.S3I ZNF821.jpg]

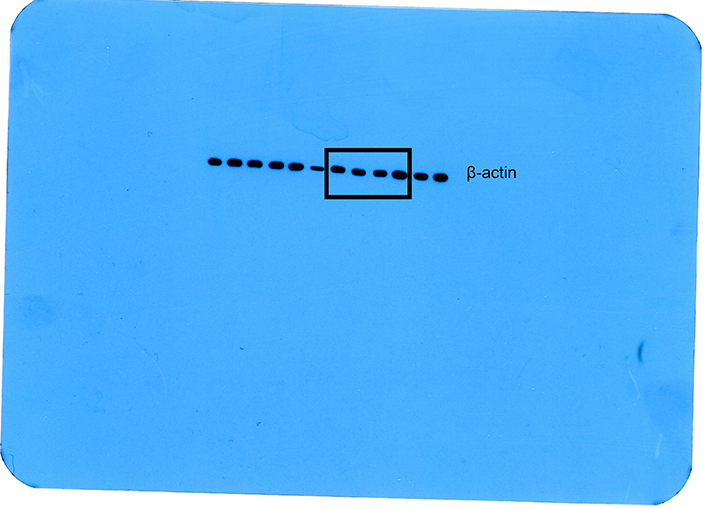

Supplement: Supplementary file 3 — Source Data [file 41467_2021_26718_MOESM3_ESM.zip › uncropped versions of any gels or blots/Fig.S3I actin.jpg]

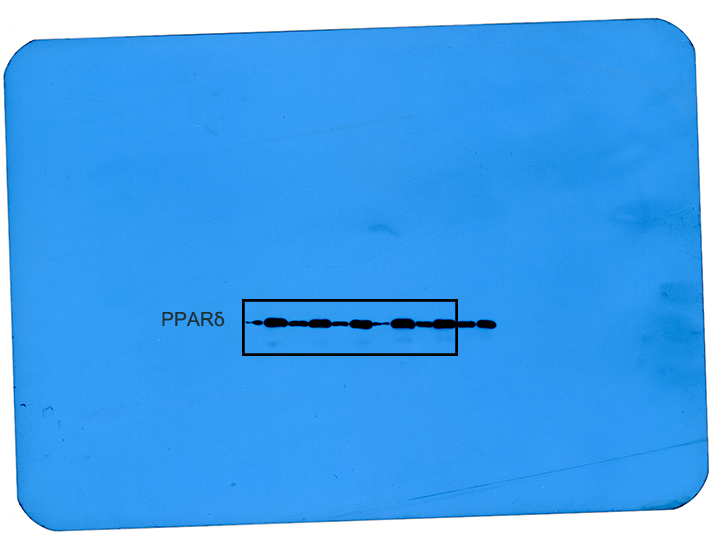

Supplement: Supplementary file 3 — Source Data [file 41467_2021_26718_MOESM3_ESM.zip › uncropped versions of any gels or blots/Fig.S4B PPARẟ.jpg]

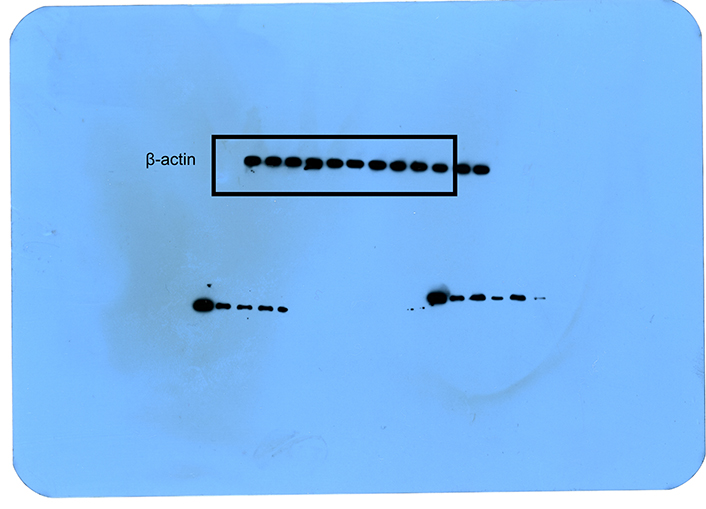

Supplement: Supplementary file 3 — Source Data [file 41467_2021_26718_MOESM3_ESM.zip › uncropped versions of any gels or blots/Fig.S4B actin.jpg]

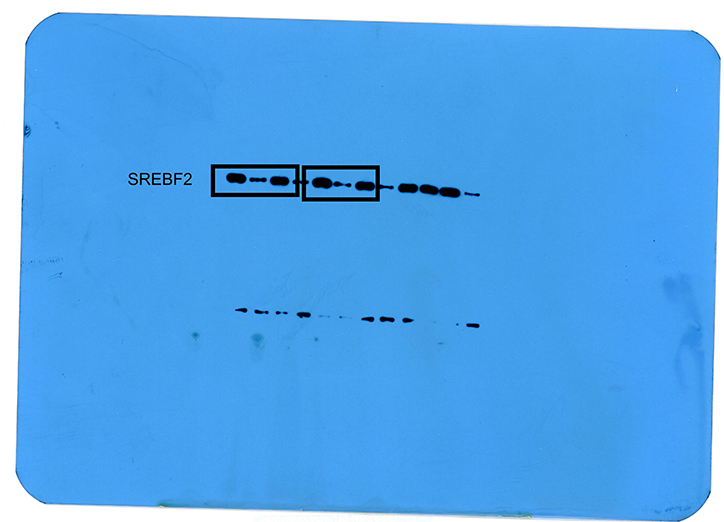

Supplement: Supplementary file 3 — Source Data [file 41467_2021_26718_MOESM3_ESM.zip › uncropped versions of any gels or blots/Fig.S5H SREBF2.jpg]

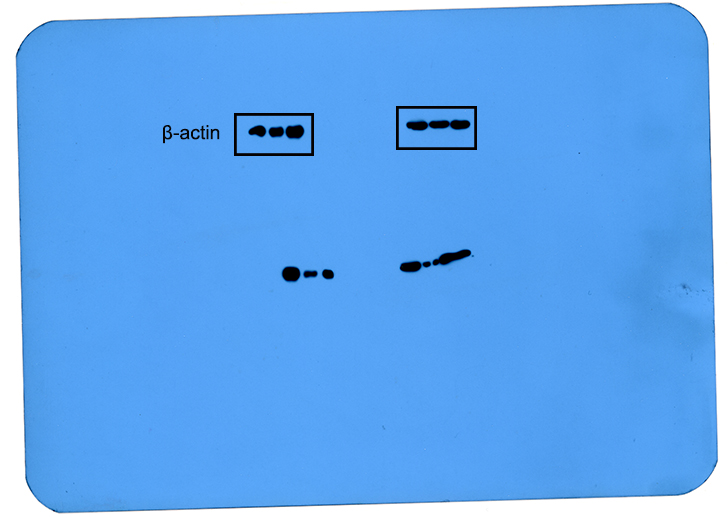

Supplement: Supplementary file 3 — Source Data [file 41467_2021_26718_MOESM3_ESM.zip › uncropped versions of any gels or blots/Fig.S5H actin.jpg]

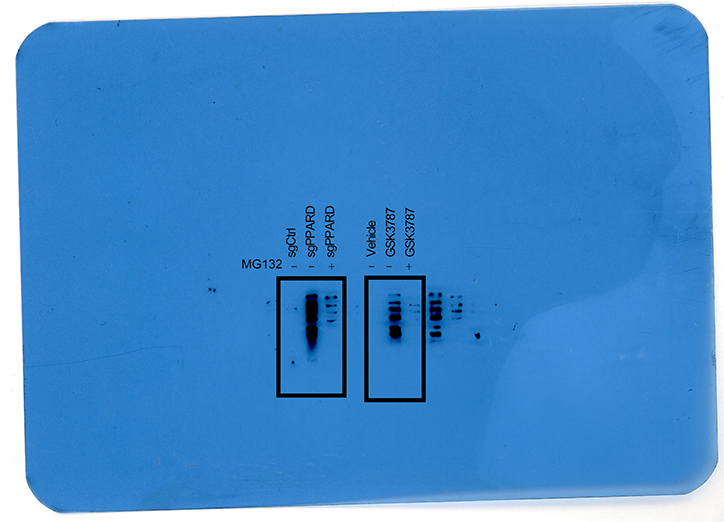

Supplement: Supplementary file 3 — Source Data [file 41467_2021_26718_MOESM3_ESM.zip › uncropped versions of any gels or blots/Fig.S5H ip SREBF2.jpg]

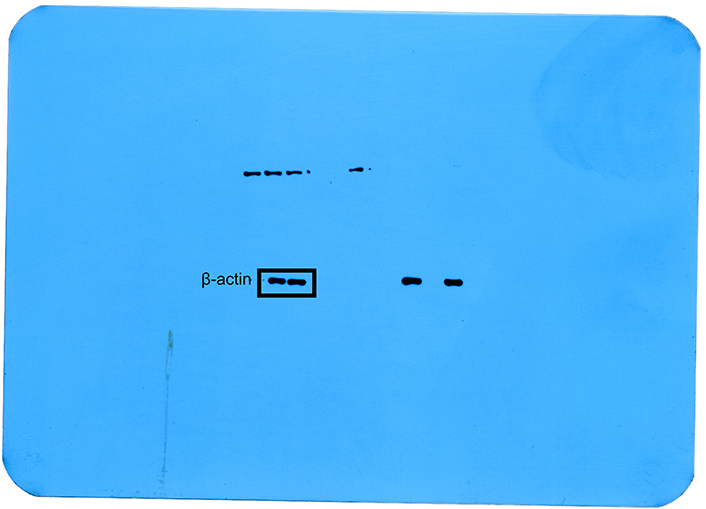

Supplement: Supplementary file 3 — Source Data [file 41467_2021_26718_MOESM3_ESM.zip › uncropped versions of any gels or blots/Fig.S5I actin.jpg]

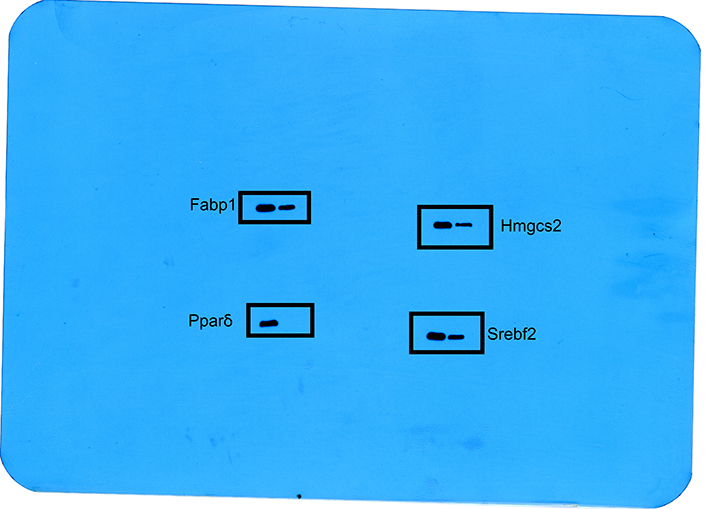

Supplement: Supplementary file 3 — Source Data [file 41467_2021_26718_MOESM3_ESM.zip › uncropped versions of any gels or blots/Fig.S5I.jpg]

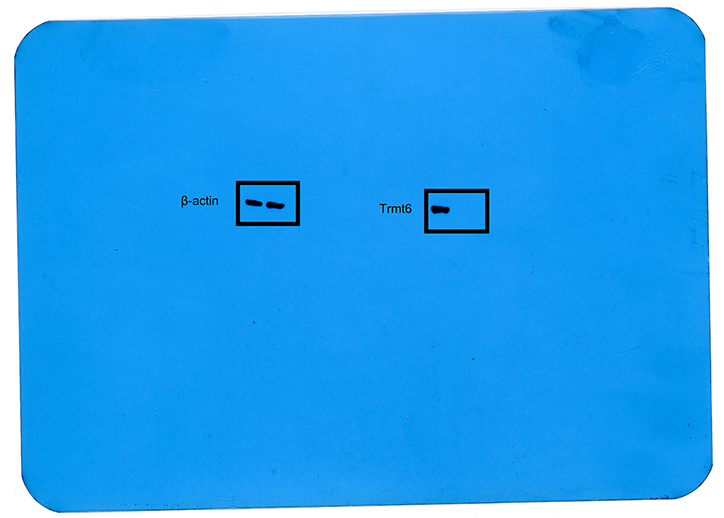

Supplement: Supplementary file 3 — Source Data [file 41467_2021_26718_MOESM3_ESM.zip › uncropped versions of any gels or blots/Fig.S6A.jpg]

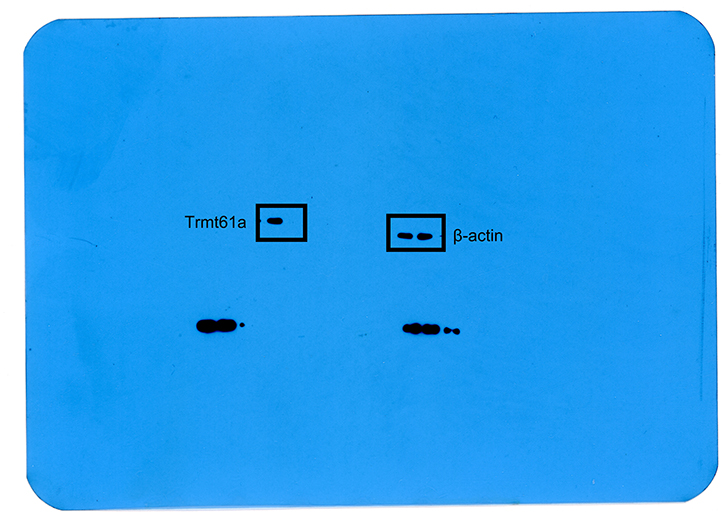

Supplement: Supplementary file 3 — Source Data [file 41467_2021_26718_MOESM3_ESM.zip › uncropped versions of any gels or blots/Fig.S6B.jpg]
